# An evolutionary genomics view on neuropeptide genes in Hydrozoa and Endocnidozoa (Myxozoa)

**DOI:** 10.1186/s12864-021-08091-2

**Published:** 2021-11-30

**Authors:** Thomas L. Koch, Frank Hauser, Cornelis J. P. Grimmelikhuijzen

**Affiliations:** Section for Cell and Neurobiology, Department of Biology, University of Copenhagen, Universitetsparken 15, DK-2100 Copenhagen, Denmark

**Keywords:** Genomics, Evolution, Neuropeptide, Nervous system, Cnidaria, Hydrozoa, Myxozoa, Fish parasite, Polypodium, Buddenbrockia

## Abstract

**Background:**

The animal phylum Cnidaria consists of six classes or subphyla: Hydrozoa, Scyphozoa, Cubozoa, Staurozoa, Anthozoa, and Endocnidozoa. Cnidarians have an early evolutionary origin, diverging before the emergence of the Bilateria. Extant members from this phylum, therefore, are important resources for understanding the evolution of the nervous system. Cnidarian nervous systems are strongly peptidergic. Using genomics, we have recently shown that three neuropeptide families (the X_1_PRX_2_amides, GRFamides, and GLWamides) are wide-spread in four (Scyphozoa, Cubozoa, Staurozoa, Anthozoa) out of six cnidarian classes or subphyla, suggesting that these three neuropeptide families emerged in the common cnidarian ancestor. In the current paper, we analyze the remaining cnidarian class, Hydrozoa, and the subphylum Endocnidozoa, to make firm conclusions about the evolution of neuropeptide genes in Cnidaria.

**Results:**

We analyzed sixteen hydrozoan species with a sequenced genome or transcriptome, using a recently developed software program for discovering neuropeptide genes. These species belonged to various hydrozoan subclasses and orders, among them the laboratory models *Hydra*, *Hydractinia*, and *Clytia*. We found that each species contained three to five neuropeptide families. A common feature for all hydrozoans was that they contained genes coding for (i) X_1_PRX_2_amide peptides, (ii) GRFamide peptides, and (iii) GLWamide peptides. These results support our previous conclusions that these three neuropeptide families evolved early in evolution. In addition to these three neuropeptide families, hydrozoans expressed up to two other neuropeptide gene families, which, however, were only occurring in certain animal groups. Endocnidozoa (Myxozoa) are microscopically small endoparasites, which are strongly reduced. For long, it was unknown to which phylum these parasites belonged, but recently they have been associated with cnidarians. We analyzed nine endocnidozoan species and found that two of them (*Polypodium hydriforme* and *Buddenbrockia plumatellae*) expressed neuropeptide genes. These genes coded for neuropeptides belonging to the GRFamide and GLWamide families with structures closely resembling them from hydrozoans.

**Conclusions:**

We found X_1_PRX_2_amide, GRFamide, and GLWamide peptides in all species belonging to the Hydrozoa, confirming that these peptides originated in the common cnidarian ancestor. In addition, we discovered GRFamide and GLWamide peptide genes in some members of the Endocnidozoa, thereby linking these parasites to Hydrozoa.

**Supplementary Information:**

The online version contains supplementary material available at 10.1186/s12864-021-08091-2.

## Background

About 700–800 million years ago, four phyla diverged from the main evolutionary lineage of animals that led to the Bilateria [1, 2]. These four animal phyla were Porifera (sponges), Ctenophora (comb jellyfishes), Placozoa (1-mm small, disk-like animals), and Cnidaria (animals like sea anemones, corals and jellyfishes). During that time period, many important genes had already evolved for organizing embryonic multicellular development and for creating the signaling pathways of early neuro-endocrine systems. Extant members of Porifera, Ctenophora, Placozoa, and Cnidaria, therefore, are invaluable resources for identifying ancestral building blocks needed for the functioning of the nervous and endocrine systems and many other processes important for multicellular animals.

From all four early-diverging taxa, nervous systems have only been demonstrated in Ctenophora [3–5] and Cnidaria [6, 7], while Porifera apparently don’t have a nervous or endocrine system and Placozoa only have endocrine cells scattered around the margins of the animal [8], but no nerve cells. The neurotransmitters in Ctenophora have not been identified yet, but both the endocrine systems in Placozoa [8–10] and the nervous systems in Cnidaria [6, 7] use neuropeptides for signal transmission, suggesting that neuropeptides must have played central roles in the evolution of early neuro-endocrine systems.

In our current paper, we want to focus on the evolution of cnidarian nervous systems. The anatomy of the cnidarian nervous system can be best described as a nerve net that on some locations has condensed to form nervous plexuses (for example around the mouth of polyps or medusae), or giant nerves (for example along the bell margins of hydromedusae) [11–18].

For long it had been a mystery, which neurotransmitters were used by the cnidarian nervous systems, but in the eighties, we discovered that cnidarian nervous systems were peptidergic. Using antibodies against the C-terminal peptide sequence RFamide, we found strongly stained nerve nets, sometimes combined with giant nerves, in the freshwater polyp *Hydra magnipapillata*, in the colonial polyp *Hydractinia echinata*, in the hydromedusa *Polyorchis penicillatus,* in sea anemones, and various other cnidarians [6, 7, 14–16, 18, 19]. Using a radioimmunoassay for the sequence RFamide, we subsequently isolated a neuropeptide from the sea anemone *Anthopleura elegantissima* and determined its structure as pQGRFamide (Antho-RFamide), the first cnidarian neuropeptide to be identified [20]. This discovery was followed by the isolation of Antho-RFamide from the octocoral *Renilla köllikeri* [21] and N-terminally elongated forms of Antho-RFamide from *H. magnipapillata*, *P. penicillatus*, and the scyphomedusa *Cyanea lamarckii* [22–25].

After this initial discovery of the cnidarian GRFamide neuropeptide family, our research group and several other laboratories isolated and sequenced various other neuropeptide family members, mainly from the sea anemone *A. elegantissima* and the freshwater polyp *H. magnipapillata* [6, 7, 26–36]. Subsequent physiological experiments showed that these neuropeptides were involved in smooth muscle contractions, larval motility, larval metamorphosis, neuronal stem cell differentiation, and sexual reproduction [6, 7, 18, 30–39].

We also cloned the cnidarian neuropeptide preprohormones [6, 7, 40–45]. These preprohormones are often characterized by a very high copy number of the immature neuropeptide in question, which can be up to thirty-seven neuropeptide copies per preprohormone [43]. Each immature neuropeptide copy is C-terminally flanked by the sequence GR, GRR, or GKR, which are established processing signals for prohormone convertase 1/3 (=PC1/3), that cleaves at the C-terminal sites of basic residues [46, 47]. The remaining C-terminal basic residues are subsequently removed by a carboxypeptidase specific for basic amino acid residues, after which the C-terminal Gly residues are converted into C-terminal amide groups by a peptidylglycine alpha-monooxygenase [48, 49]. After their release into the intercellular space, these C-terminal amide groups protect the neuropeptides against further degradation by unspecific carboxypeptidases, thereby increasing the stability of these signal molecules [48]. In addition, the C-terminal amide groups are essential for proper G protein-coupled receptor (GPCR) binding, since peptides with a free C-terminal carboxyl group or C-terminal extension with a Gly residue often lack biological activity [48].

At the N-termini of the immature neuropeptide copies are frequently Q residues, and sometimes XP, or XPP sequences. The Q residues are converted into pQ (= pyroglutaminyl) residues by glutaminyl cyclase [50]. Together with the XP, or XPP residues, these pQ groups protect released cnidarian neuropeptides against N-terminal degradation by unspecific aminopeptidases [50]. In Bilateria, the immature neuropeptide copies are flanked, both C- and N-terminally, by R, RR, or KR residues [46–48]. In cnidarians, however, these sites are only present at the C-termini of the immature peptide sequences, while at the N-termini, the Q, XP, and XPP residues are often preceded by acidic groups (E or D), and N, S, T, or several other amino acid residues [6]. We assume, therefore, that one or more unspecific and yet unknown aminopeptidases are involved in the N-terminal processing of immature cnidarian neuropeptides. It is interesting that we can observe the same phenomenon in the placozoan preprohormones published by Nikitin [9], suggesting that Placozoa and Cnidaria are phylogenetically closely related.

The phylum Cnidaria consists of six classes or subphyla: Anthozoa (sea anemones and corals), Hydrozoa (polyps like *Hydra* and *H. echinata*), Scyphozoa (true jellyfishes), Cubozoa (box jellyfishes), Staurozoa (stalked jellyfishes), and Endocnidozoa (microscopically small parasites, mostly parasitizing fish).

**Fig. 1:**
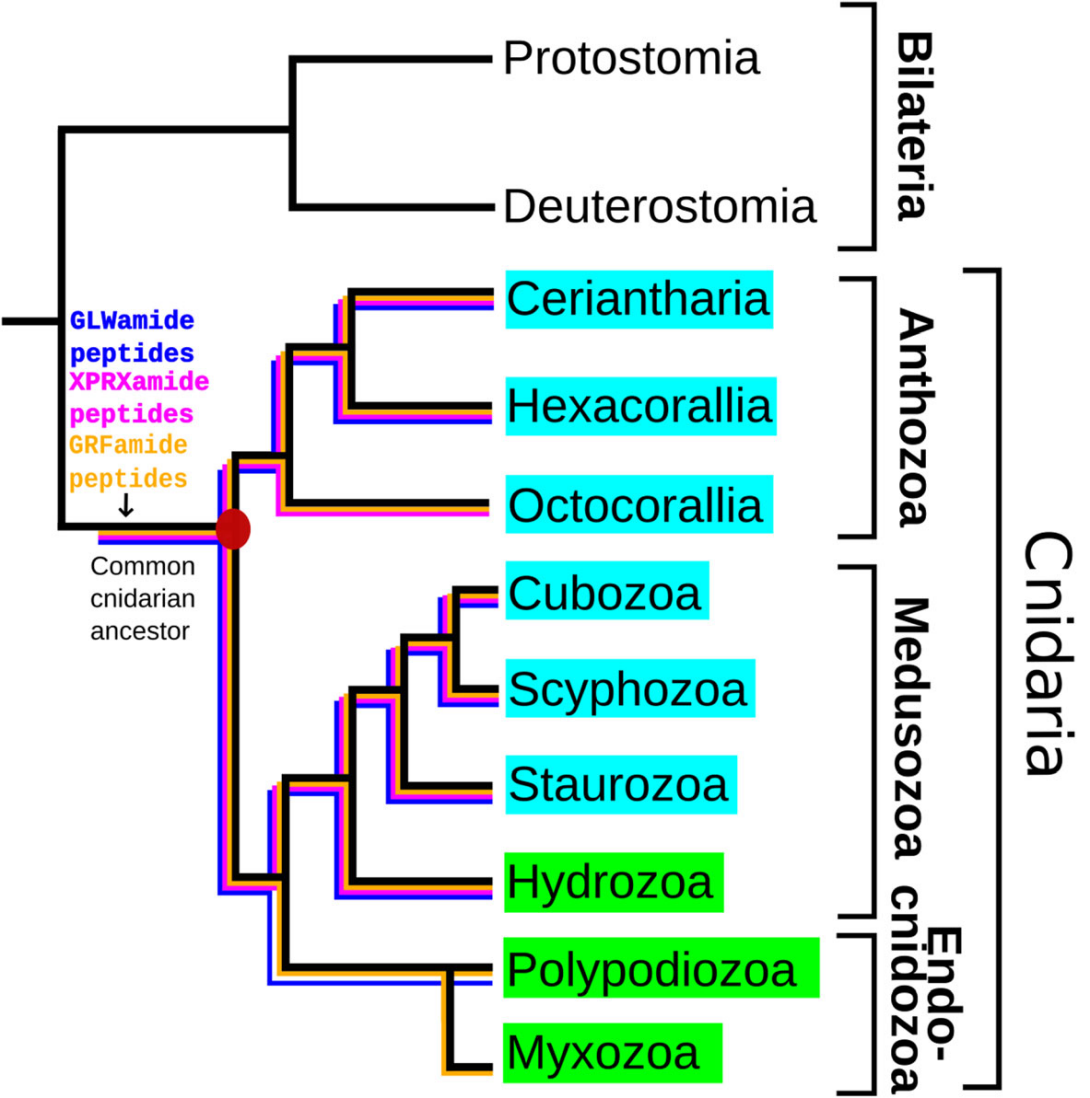
Schematic drawing showing the phylogenetic positions of the subclasses Ceriantharia, Hexacorallia and Octocorallia (class Anthozoa), the classes Hydrozoa, Cubozoa, Scyphozoa, Staurozoa, and the subphylum Endocnidozoa. Cnidarians are a sister group to Bilateria. The figure also shows that X_1_PRX_2_amide (highlighted in purple), GRFamide (highlighted in yellow), and GLWamide peptides (highlighted in blue) are present in all tested Ceriantharia, Hexacorallia, Cubozoa, Scyphozoa, and Staurozoa species. However, the Octocorallia have apparently lost their GLWamide genes [52]. In the current paper, we are investigating the presence of these three neuropeptide families in Hydrozoa and Endocnidozoa. We found that hydrozoans contain all three neuropeptide families (X_1_PRX_2_amides, GRFamides, GLWamides). The endocnidozoans contain GRFamides and GLWamides, but have apparently lost their X_1_PRX_2_amide genes. In this figure we have presented the Endcnidozoa as two sister taxa, the Polypodiozoa and Myxozoa, but their mutual phylogenetic relationship is unclear

Because most neuropeptides had been isolated from the anthozoan *A. elegantissima* and the hydrozoan *H. magnipapillata,* we were wondering whether all cnidarian classes had these same sets of neuropeptides. Furthermore, we also hoped that additional neuropeptides with important functions were to be discovered in other, so far unexplored, cnidarians. For these reasons, we recently started large-scale analyses of all cnidarians with a sequenced genome or transcriptome, using bioinformatics and a software program that we especially designed for discovering cnidarian preprohormone genes [51]. After having analyzed five cubozoan species, four scyphozoan species, six staurozoan species, seven species belonging to the Octocorallia (a subclass of Anthozoa), nineteen species belonging to Hexacorallia (a subclass of Anthozoa), and one Ceriantharia (a subclass of Anthozoa) species – thus analyzing altogether 80 genome or transcriptome databases - we found that three neuropeptide families turned out to be wide-spread in Cnidaria: The X_1_PRX_2_amide, GRFamide, and GLWamide families [52, 53]. Based on the phylogenetic positions of the analyzed cnidarian classes and subclasses, we concluded that these three neuropeptide families must have evolved in the common ancestor of Cnidaria, perhaps together with the emergence of the first nervous systems (Fig. 1). In addition to the three wide-spread neuropeptide families (Fig. 1), we also identified several neuropeptide genes that were confined to one cnidarian class or order. We assumed that these genes had evolved to serve class- or order-specific physiological processes.

Two cnidarian classes/subphyla, the Hydrozoa and Endocnidozoa (Myxozoa), remained to be analyzed (highlighted green in Fig. 1). Hydrozoans have a life cycle that often includes a swimming planula larva, a polyp, and a medusa stage. Several important laboratory models for animal development, regeneration, and aging belong to the Hydrozoa, such as *Hydra*, *Hydractinia*, and *Clytia* [54–56]. Endocnidozoa (Myxozoa) are microscopically small (10–300 μm long) endoparasites, often parasitizing fish, which make them a serious economic burden for aquaculture [57, 58]. These parasites are highly reduced, often lacking organs and tissues like a mouth, gut, muscles, or a nervous system. In some life stages of endocnidozoans, however, a nervous system could be observed, such as in *Polypodium hydriforme*, which in its free-living (bottom-dwelling) stage contains a nerve net, expressing GRFamide neuropeptides [59]. For long, the phylogenetic position of Endocnidozoa has been a mystery, but recently they have been linked as a sister group to the cnidarian subphylum Medusozoa (consisting of the classes Cubozoa, Scyphozoa, Staurozoa, and Hydrozoa; Fig. 1) [60].

In our current paper, we have investigated hydrozoans and endocnidozoans not only to obtain a more solid picture of the evolution of cnidarian neuropeptides (Fig. 1), but also to gain more knowledge about their biology. This knowledge might help us to better understand hydrozoan laboratory models, as well as a large group of economically important fish parasites.

## Results

### Mining of genomic and transcriptomic databases from hydrozoans for neuropeptide genes

We investigated the published genomes or transcriptomes from sixteen hydrozoan species, altogether comprising 21 databases (Table 1). The accession numbers of these databases are given in Table 1 except for the transcript data from *C. hemisphaerica* that we accessed through the Transcript Browser offered at http://marimba.obs-vlfr.fr/organism/Clytia/hemisphaerica. All databases were analyzed using a script that we specifically developed for identifying unknown cnidarian preprohormones that contained three or more neuropeptide copies [51]. In addition, we also applied TBLASTN, using a collection of known neuropeptide sequences as a query.

**Table 1:** Accession numbers for the different hydrozoan databases used

Species	Subclass	Order	Database type	Accession number and reference (if published)
*Dynamena pumila*	Hydroidolina	Anthoathecata	TSA	GHMC00000000.1
*Hydra magnipapillata*	Hydroidolina	Anthoathecata	TSA	GAOL00000000.1
*Hydra oligactis*	Hydroidolina	Anthoathecata	TSA WGS	GBFD00000000.1 PJUT00000000.1 [61];
*Hydra vulgaris*	Hydroidolina	Anthoathecata	TSA TSA WGS	GEVZ00000000.1 [62]; GGKH00000000.1 ACZU00000000.1 [63];
*Hydractinia symbiolongicarpus*	Hydroidolina	Anthoathecata	TSA	GAWH00000000.1 [64];
*Millepora alcicornis*	Hydroidolina	Anthoathecata	TSA	GFAS00000000.1
*Millepora squarrosa*	Hydroidolina	Anthoathecata	TSA	GFGU00000000.1
*Millepora complanata*	Hydroidolina	Anthoathecata	TSA	GFGT00000000.1
*Millepora* sp.	Hydroidolina	Anthoathecata	TSA	GFGV00000000.1
*Podocoryna carnea*	Hydroidolina	Anthoathecata	TSA TSA	GCHV00000000.1 GBEH00000000.1
*Porpita porpita*	Hydroidolina	Anthoathecata	TSA	GHBA00000000.1
*Turritopsis* sp.	Hydroidolina	Anthoathecata	TSA	HAAD00000000.1 [65];
*Velella velella*	Hydroidolina	Anthoathecata	TSA	GHAZ00000000.1
*Clytia hemisphaerica*	Hydroidolina	Leptothecata	WGS TSA	N/A^a^
*Physalia physalis*	Hydroidolina	Siphonophora	TSA	GHBB00000000.1
*Craspedacusta sowerbii*	Trachylinae	Limnomedusae	WGS	QQSS00000000.1

^a^downloaded from http://marimba.obs-vlfr.fr/organism/Clytia/hemisphaerica

### X_1_PRX_2_amide preprohormones in colonial hydrozoans

Table 2, neuropeptide family number-1 (first section), shows X_1_PRX_2_amide neuropeptide sequences discovered in eight colonial hydrozoan species, belonging to the order Anthoathecata.

**Table 2:**
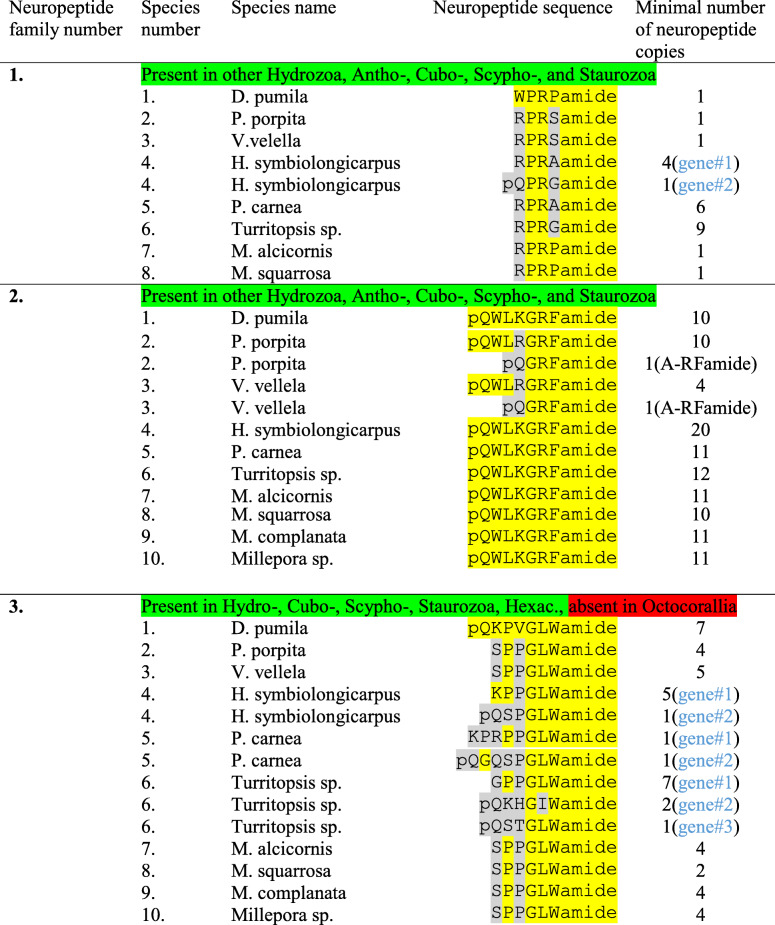
Three neuropeptide families (#1–3) identified in ten hydrozoan species, belonging to the order Anthoathecata: *Dynamena pumila*, *Porpita porpita*, *Velella velella*, *Hydractinia symbiolongicarpus*, *Podocoryna carnea*, *Turritopsis* sp., *Millepora alcicornis, Millepora squarrosa, Millepora complanata*, and *Millepora* sp. Only those neuropeptides that have multiple identical or similar copies on their preprohormones are listed and of these only those with the highest copy numbers are given. If more than one gene codes for the peptides, this is highlighted in blue in the last column. The amino acid sequences of the preprohormones are shown in Additional file 1 to Additional file 3. A-RFamide means Antho-RFamide [20]

In *Dynamena pumila* we identified one incomplete preprohormone with one copy of WPRPamide, one copy of FPRGamide and four other related peptides (Table 2, Additional file 1).

In *Porpita porpita*, we identified a gene, coding for a complete preprohormone with one copy of RPRSamide and one copy of another related peptide (Table 2, Additional file 1).

In *Velella velella*, we found a gene, encoding a complete preprohormone with one copy of RPRSamide and one other related N-terminally elongated peptide sequence (Table 2, Additional file 1).

In *Hydractinia symbiolongicarpus*, we identified two genes: Gene#1, coding for a complete preprohormone containing four copies of RPRAamide and one copy of SPRGamide; and gene#2, coding for a complete preprohormone with one single copy of pQPRGamide (Table 2, Additional file 1).

In *Podocoryna carnea*, we found one gene coding for a complete preprohormone containing six copies of RPRAamide and one copy of KPRGamide (Table 2, Additional file 1).

In *Turritopsis* sp., we identified an incomplete preprohormone coding for nine copies of RPRGamide and four pQLLRGamide sequences, the last ones not being genuine X_1_PRX_2_amides (Table 2, Additional file 1).

In *Millepora alcicornis*, we identified a complete preprohormone containing one copy of RPRPamide and one copy of IPRMamide. In *Millepora squarrosa*, we found a nearly identical preprohormone with one copy of RPRPamide and one copy of IPRLamide (Table 2, Additional file 1). In the transcriptomic databases from the other two *Millepora* species, *Millepora complanata* and *Millepora* sp. (Table 1), we were unable to identify X_1_PRX_2_amide sequences, perhaps due to the low qualities of these data sets.

In Table 3, we summarize the X_1_PRX_2_amide neuropeptides identified in three colonial species, each belonging to a separate hydrozoan order: Limnomedusae, Leptothecata, and Siponophora.

**Table 3:**
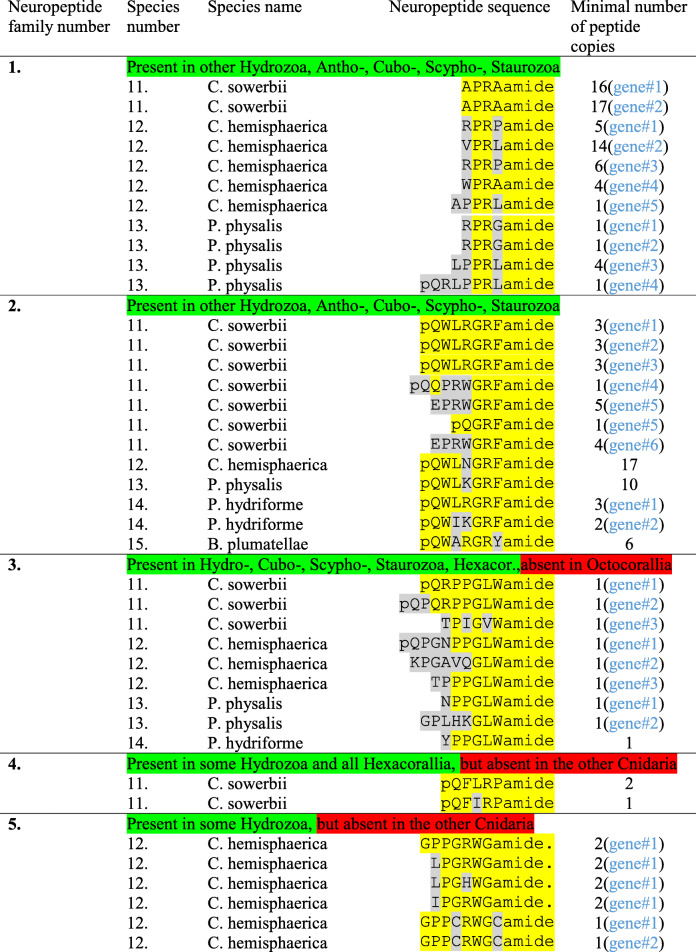
Neuropeptide families identified in three hydrozoan orders: *Craspedacusta sowerbii* (Limnomedusae), *Clytia hemisphaerica* (Leptothecata), and *Physalia physalis* (Siphonophora). We also show neuropeptide sequences for two parasitic cnidarians: *Polypodium hydriforme* (class Polypodiozoa; order Polypodiidea) and *Buddenbrockia plumatellae* (class Myxozoa; order Malacosporea). This table is presented in the same way as Table 2. The preprohormones are shown in Additional files 1-5

In the freshwater medusa *Craspedacusta sowerbii* (Limnomedusae), we identified two genes: Gene#1 codes for a complete preprohormone, containing sixteen copies of APRAamide and one additional related neuropeptide sequence; Gene#2 codes for a complete preprohormone having seventeen copies of APRAamide and one related neuropeptide copy. Both preprohormones resemble each other and their genes are likely to be paralogues (Table 3, Additional file 1).

In *Clytia hemisphaerica* (order Leptothecata), we identified five different X_1_PRX_2_amide preprohormone genes (Additional file 1). Gene#1 codes for a complete preprohormone with five copies of RPRPamide and two other X_1_PRX_2_amide peptides (Table 3; Additional file 1). Gene#2 codes for 14 copies of VPRLamide and one other X_1_PRX_2_amide peptide; Gene#3 for six copies of RPRPamide and six copies of WPRPamide; Gene#4 for four copies of WPRAamide and seven other X_1_PRX_2_amide peptides; Gene#5 for one copy of APPRLamide and one copy of WPPRLamide. The last two peptides are not sensu stricto X_1_PRX_2_amide peptides, but are closely related. The first four X_1_PRX_2_amide preprohormones (coded for by Gene#1 to Gene#4) have previously been discovered in *C. hemisphaerica* [35]. However, some of these preprohormones differ considerably from the ones that we have currently identified (explained in Additional file 1). Gene#5 has not been previously identified.

In the “Portuguese man o war”, *Physalia physalis* (Siphonophora), we found four genes: Gene#1, coding for a complete preprohormone with one copy of RPRGamide and another related peptide; Gene#2, coding for a complete preprohormone with one copy of RPRGamide and another related peptide; Gene#3, coding for a complete preprohormone with four copies of LPPRLamide and three other related peptides; and Gene#4, coding for an incomplete preprohormone with one copy of pQRLPPRLamide. The last two mentioned peptide sequences are not X_1_PRX_2_amides sensu stricto, but are related peptides (Table 3, Additional file 1).

### GRFamide preprohormones in colonial hydrozoans

In Table 2, middle section (named: Neuropeptide family number-2), we give the GRFamide neuropeptide sequences that we discovered in eight colonial hydrozoan species, belonging to the order Anthoathecata.

In *D. pumila*, we identified one gene, coding for an incomplete preprohormone with ten copies of the sequence pQWLKGRFamide and one copy of another neuropeptide (Table 2, Additional file 2).

In *P. porpita*, we identified one gene, coding for a complete preprohormone, containing ten copies of pQWLRGRFamide, one copy of pQGRFamide, and three other neuropeptide sequences (Table 2, Additional file 2). The presence of pQGRFamide is interesting, as this peptide (named Antho-RFamide) has, so far, only been found in anthozoans [20, 21, 40, 41, 52, 53].

In *V. velella* (Anthoathecata), we found one gene fragment, coding for an incomplete preprohormone, containing four copies of pQWLRGRFamide, and another fragment coding for two copies of pQWLRGRFamide, one copy of pQGRFamide (=Antho-RFamide), and another peptide sequence (Table 2, Additional file 2).

In *H. symbiolongicarpus*, we identified one gene, coding for a complete preprohormone, containing twenty copies of the neuropeptide pQWLKGRFamide and two other neuropeptide sequences (Table 2, Additional file 2).

In *P. carnea*, we found one gene, coding for a complete preprohormone, containing eleven copies of pQWLKGRFamide and no further neuropeptide sequences (Table 2, Additional file 2).

In *Turritopsis* sp., we identified one gene, coding for a complete preprohormone, containing twelve copies of pQWLKGRFamide and no other neuropeptide sequences (Table 2, Additional file 2).

In *M. alcicornis*, we identified one gene, coding for a complete preprohormone, containing eleven copies of pQWLKGRFamide and one copy of pQWHAGRFamide (Table 2; Additional file 2). In the transcriptome databases from *M. complanata* and *Millepora* sp. (Table 1), we found genes coding for preprohormones that were identical to the one from *M. alcicornis* (Additional file 2). We consider that these results might be due to *M. alcicornis/M. complanata/Millepora* sp. being the same or very closely related species. The preprohormone found in the dataset from *M. squarrosa* was somewhat different and contained a complete preprohormone sequence with ten copies of pQWLKGRFamide and one copy of pQWHAGRFamide (Table 2; Additional file 2).

The three hydrozoan species shown in Table 3 each belong to a separate hydrozoan order: Limnomedusae, Leptothecata, and Siponophora.

In the freshwater medusa *C. sowerbii* (Limnomedusae), we identified no less than six incomplete genes, coding for GRFamide peptides (Table 3, neuropeptide family number 2): Gene#1 codes for a preprohormone containing at least three copies of pQWLRGRFamide and one other peptide sequence; Genes#2 and #3 code for preprohormones that, although different in structure, contain the same number and same type of peptides. Because the three genes resemble each other so much (Additional file 2), they might be allelic variants; Gene#4 codes for one copy of pQQPRWGRFamide and five other related peptides; Gene#5 codes for five copies of EPRWGRFamide, one copy of Antho-RFamide (pQGRFamide) and ten other, related neuropeptides; Gene#6 codes for four copies of EPRWGRFamide and nine other peptide sequences (Table 3, Additional file 2).

In *C. hemisphaerica* (Leptothecata) we found one gene, coding for a complete preprohormone with seventeen copies of pQWLNGRFamide and one copy of pQLVSGRFamide (Table 3, Additional file 2).

In *P. physalis* (Siphonophora), we found one gene, coding for a complete preprohormone with ten copies of the neuropeptide sequence pQWLKGRFamide and no additional sequences (Table 3, Additional file 2).

### GLWamide preprohormones in colonial hydrozoans

Below, we summarize the GLWamide neuropeptide sequences that we identified in colonial hydrozoans, belonging to the order Anthoathecata.

In *D. pumila*, we found one gene coding for a preprohormone with seven copies of pQKPVGLWamide and nine other GLWamide peptide sequences (Table 2, Additional file 3).

In *P. porpita*, we identified one gene, coding for a complete preprohormone with four copies of SPPGLWamide and one additional GLWamide peptide (Table 2, Additional file 3).

In *V. vellela*, we identified one gene, coding for a complete preprohormone, containing five copies of SPPGLWamide and one further peptide sequence (Table 2, Additional file 3).

In *H. symbiolongicarpus*, we found two genes: Gene#1, coding for a complete preprohormone with five copies of KPPGLWamide and one other GLWamide peptide; and Gene#2, coding for a complete preprohormone, having only one copy of pQSPGLWamide and one other potential peptide sequence (Table 2, Additional file 3).

In *P. carnea*, we identified two genes: Gene#1, coding for a complete preprohormone with one copy of KPRPPGLWamide and two other related neuropeptide sequences; and Gene#2 with one copy of pQGQSPGLWamide and three other related neuropeptide sequences (Table 2, Additional file 3).

In *Turritopsis* sp., we found three genes: Gene#1, coding for a complete preprohormone, containing seven copies of GPPLWamide and one other GLWamide peptide; Gene#2, coding for a complete preprohormone with two copies of pQKHGIWamide and one other neuropeptide sequence; and Gene#3, coding for a complete preprohormone with one copy of pQSTGLWamide and one other peptide sequence (Table 2, Additional file 3).

For *M. alciconis*, *M. complanata*, and *Millepora* sp. we were confronted by the same phenomenon as for the GRFamide preprohormones (see above), namely that the three transcriptome databases (Table 1) yielded identical GLWamide preprohormones, suggesting that these three species might be the same. All three GLWamide preprohormones contained four copies of SPPGLWamide, one copy of NPPGVWamide, and one copy of RPPGVWamide (Table 2, Additional file 3). In *M. squarrosa*, however, we identified a clearly different GLWamide preprohormone, confirming (see above) that this is a separate species. This preprohormone contains two copies of SPPGLWamide, two copies of SPPGVWamide, one copy of NPPGVWamide, and one copy of GPPGLWamide (Table 2, Additional file 3).

The three hydrozoan species discussed below, each belong to a different hydrozoan order: Limnomedusae, Leptothecata, and Siponophora.

In *C. sowerbii* (Limnomedusae), we discovered three genes: Gene#1, coding for a complete preprohormone, containing one copy of pQRPPGLWamide and five other neuropeptide sequences; Gene#2, coding for a complete preprohormone, having one copy of pQPQRPPGLWamide and two other neuropeptide sequences; and Gene#3, coding for a complete preprohormone with one copy of TPIGVWamide and one other neuropeptide (Table 3, Additional file 3).

*C. hemisphaerica* (Leptothecata) has three genes, coding for GLWamide preprohormones (Table 3): Gene#1 codes for a preprohormone, containing one copy of pQPGNPPGLWamide and two other GLWamide peptides. This preprohormone was published recently and dubbed Che-pp11 [35] (Additional file 3). Gene#2 codes for a preprohormone, having one copy of pQNSPGALGLWamide and one other GLWamide peptide (Additional file 3). Also this preprohormone was identified previously and dubbed Che-pp2 [35]. We discovered a third preprohormone in *C. hemisphaerica* coded for by gene#3 that had interesting, mixed-type properties. It contained one copy of a GLWamide family member, TPPPGLWamide (Table 3), four copies of a novel peptide LPMKFamide, and two other peptide sequences (Additional file 3).

In *D. physalis* (Siphonophora), we found two genes: Gene#1 is coding for a complete preprohormone with one copy of NPPGLWamide and five other GLWamide neuropeptides; while Gene#2 is coding for a complete preprohormone with one copy of GPLHKGLWamide and eight copies of other GLWamide neuropeptides (Table 3, Additional file 3).

### LRPamide preprohormones in colonial hydrozoans

The freshwater medusa, *C. sowerbii* (Limnomedusae), is the only colonial hydrozoan tested in the current paper that has a gene, coding for an LPRamide preprohormone (Additional file 4). This preprohormone contains two copies of pQFLRPamide and one copy of pQFIRPamide (Table 3, neuropeptide family number 4).

### RWGamide preprohormones in colonial hydrozoans

The colonial hydrozoan *C. hemisphaerica* (Leptothecata) expresses genes coding for RWGamide preprohormones. One of these preprohormones was published previously [35] and contains two copies of the neuropeptide GPPGRWGamide, two copies of LPGRWGamide, two copies of LPGHWGamide, two copies of IPGRWGamide, and one copy of GPPCRWGCamide. This last sequence was not recognized by the authors of [35] as a neuropeptide, but it certainly is and has quite an interesting structure, because it becomes cyclic after the formation of a cystine bridge between the two C residues (Table 3, Additional file 5). We identified a second RWGamide preprohormone fragment in *C. hemisphaerica* that contains one copy of the probable cyclic neuropeptide GPPCRWGCamide and one copy of TPGRWSamide (Table 3, Additional file 5).

### X_1_PRX_2_amide preprohormones in *Hydra*

*Hydra* has a larger number and a larger diversity of neuropeptide preprohormones than the above-described hydrozoans. In addition, the analyses of the *Hydra* genomes and transcriptomes have been further complicated, because some databases have fused two of the *Hydra* species, *Hydra magnipapillata* and *Hydra vulgaris*, into one species, *Hydra vulgaris*, while other databases have preserved the traditional name *Hydra magnipapillata* (Table 1). The arguments used for fusing the two species, were apparently that in a phylogenomic analysis of a large number of *Hydra* species, *H. magnipapilla, H. vulgaris*, but also other *Hydra* species, grouped into one clade, while other species grouped in different clades [66]. These findings, however, might not be sufficient for assigning *H. magnipapilla* and *H. vulgaris* as one species. To avoid any confusion about the species that we analyzed, therefore, we have always followed the species name that was indicated by the database. Below, we will describe *H. magnipapillata*, *H. oligactis*, and *H. vulgaris*.

*H. magnipapillata* has two genes that code for X_1_PRX_2_amide peptides: Gene#1 codes for a complete preprohormone with one copy of RPRAamide and one copy of FPQSFLPRGamide (Table 4, neuropeptide family 1; Additional file 1). This second peptide, FPQSFLPRGamide, is not an X_1_PRX_2_amide peptide sensu stricto, but is N-terminally elongated. FPQSFLPRGamide has been isolated from extracts of *H. magnipapillata*, sequenced and dubbed Hym-355, so its existence has been established [33]. Also the cDNA from Gene#1 has been cloned previously, confirming its expression in *H. magnipapillata* [33]. Gene#2 is new and codes for a complete preprohormone with one copy of RPRPamide and another peptide sequence, pQDYAPRGamide. Again, the second peptide is not an X_1_PRX_2_amide sensu stricto, but N-terminally elongated.

**Table 4:**
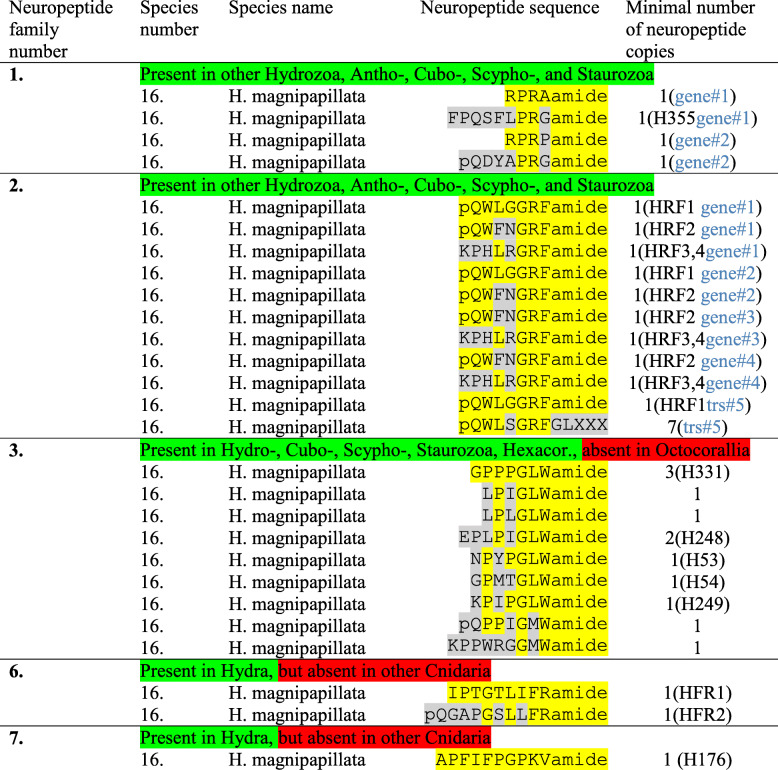
Neuropeptide families identified in *Hydra magnipapillata*

*Abbreviations*: H53/H54/H176/H248/H249/H331/H355 = the sequenced neuropeptides Hym-53/− 54/− 176/− 248/− 249/− 331/− 355 [31–33]; HFR1/HFR2 = the sequenced neuropeptides Hydra-FRamides-1 and -2 [34]; HRF1–4 = the sequenced neuropeptides Hydra-RFamides-1 to − 4 [22]; trs = transcript. If more than one gene or transcript codes for the peptides, this is highlighted in blue in the last column. The amino acid sequences of the preprohormones are shown in Additional files 1 to 3, 6, 7

*H. oligactis* has also two genes coding for X_1_PRX_2_amide peptides. Gene#1 is similar (but not identical) to gene#1 from *H. magnipapillata*, the difference mainly being in the signal sequence (Additional file 1). It codes for one copy of RPRAamide and one copy of FPQSFLPRGamide (Table 5; Additional file 1). Gene#2 is also similar to Gene#2 from *H. magnipapillata*, again the main difference being in the signal sequence (Additional file 1). As for *H. magnipapillata*, the Gene#2 from *H. oligactis* codes for one copy of RPRPamide and one copy of pQDYAPRGamide (Table 5; Additional file 1).

**Table 5:**
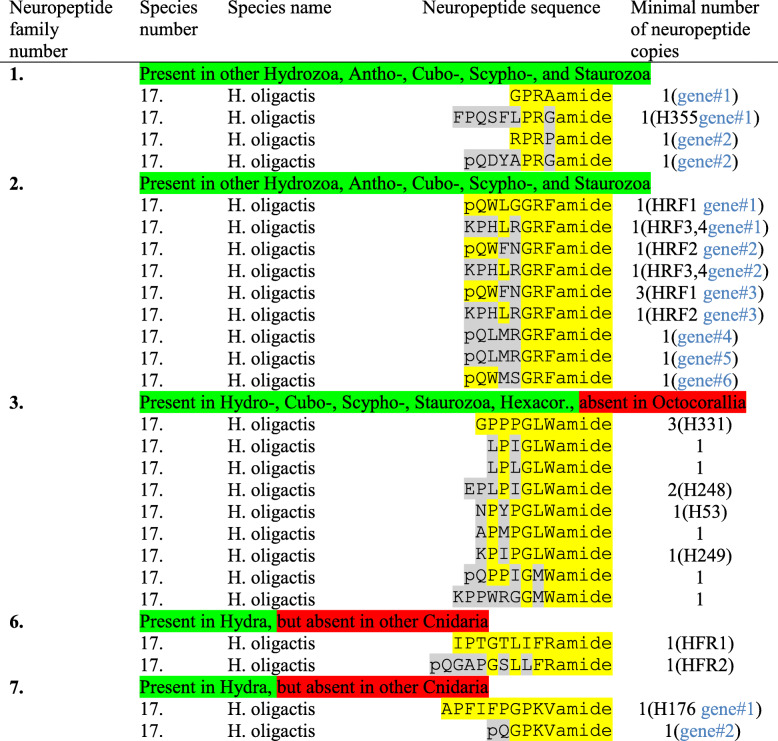
Neuropeptide families identified in *Hydra oligactis*

*Abbreviations*: H53/H176/H248/H249/H331/H355 = the sequenced neuropeptides Hym-53/176/− 248/− 249/− 331/− 355 [31–33]; HFR1/HFR2 = the sequenced neuropeptides Hydra-FRamides-1 and -2 [34]; HRF1–4 = the sequenced neuropeptides Hydra-RFamides-1 to − 4 [22]; trs = transcript. If more than one gene or transcript codes for the peptides, this is highlighted in blue in the last column. The amino acid sequences of the preprohormones are shown in Additional files 1 to 3, 6, 7

*H. vulgaris* has two X_1_PRX_2_amide preprohormone genes that are identical to the two genes from *H. magnipapillata* (Table 6; Additional file 1), suggesting that the two species may be identical.

**Table 6:**
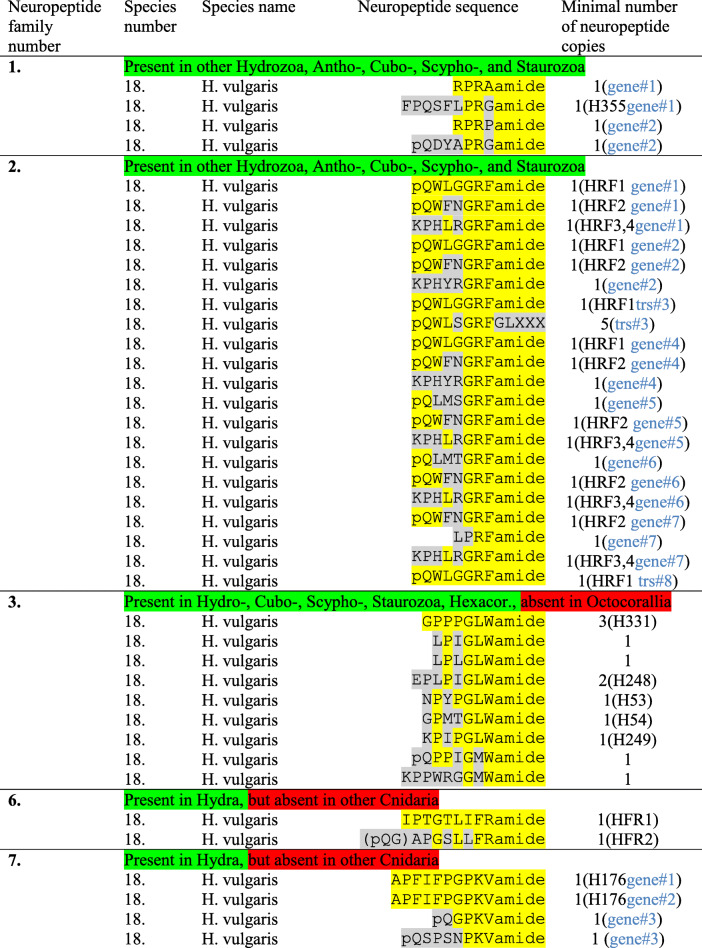
Neuropeptide families identified in *Hydra vulgaris*

*Abbreviations*: H53/H54/H176/H248/H249/H331/H355 = the sequenced neuropeptides Hym-53/− 54/− 176/− 248/− 249/− 331/− 355 [31–33]; HFR1/HFR2 = the sequenced neuropeptides Hydra-FRamides-1 and -2 [34]; HRF1–4 = the sequenced neuropeptides Hydra-RFamides-1 to − 4 [22]; trs = transcript. If more than one gene or transcript codes for the peptides, this is highlighted in blue in the last column. The amino acid sequences of the preprohormones are shown in Additional files 1 to 3, 6, 7

### GRFamide preprohormones in *Hydra*

We have previously isolated and sequenced four GRFamide peptides from extracts of *H. magnipapillata*: pQWLGGRFamide (dubbed Hydra-RFamide-1), pQWFNGRFamide (Hydra-RFamide-2), KPHLRGRFamide (Hydra-RFamide-3), and HLRGRFamide (Hydra-RFamide-4) [22]. Subsequently, we cloned the complete preprohormone for these four neuropeptides (dubbed preprohormone A), which contained one copy each of Hydra-RFamide-1, − 2, − 3, and two additional, novel Hydra-RFamide peptides that had not been sequenced before [45]. Hydra-RFamide-4 was found to be part of the Hydra-RFamide-3 sequence (there was no separate Hydra-RFamide-4 sequence on the preprohormone) and must have been generated by the removal of the N-terminal KP sequence, probably catalyzed by a dipeptidyl aminopeptidase [43, 45]. Inspection of the *H. magnipapillata* genome sequence revealed one preprohormone (Gene#1) that was identical to the cloned preprohormone A (Table 4, additional file 2). Here, we refer to Gene#1, although in the following paragraphs it became clear to us, that in some cases we needed to refer to transcripts, because some "genes" turned out to be splice variants.

We also previously cloned a second, complete GRFamide preprohormone from *H. magnipapillata* (dubbed preprohormone B) that was somewhat different from preprohormone A, but still contained one copy of Hydra-RFamide-1, one copy of Hydra-RFamide-2, one copy of KPHYRGRFamide (one amino acid residue different from Hydra-RFamide-3/4), and three additional novel Hydra-RFamide-like peptides [45]. Also, this preprohormone B could be detected in its identical form in the sequenced genome from *H. magnipapillata* (Gene#2, Table 4; Additional file 2).

We identified a novel GRFamide preprohormone in the sequenced genome from *H. magnipapillata*, coded for by Gene#3 (Additional file 2), that contained one copy of Hydra-RFamide-2, one copy of Hydra-RFamide 3/4, and one additional novel Hydra-RFamide sequence (Table 4).

Furthermore, we identified a Gene#4 in the genomic sequence from *H. magnipapillata* that coded for an additional, complete preprohormone sequence, containing one copy of Hydra-RFamide-2, one copy of Hydra-RFamide-3/4, and one other Hydra-RFamide peptide (Table 4; Additional file 2).

Previously, we also cloned a complete Hydra-RFamide preprohormone from *H. magnipapillata* that was quite different from the above-mentioned preprohormones [45]. This preprohormone, dubbed preprohormone C, contained five copies of the non-amidated sequence pQWLSGRFGLTNH, one copy of pQWLSGRFGLPNQ, one copy of pQWFSGRFGLTNQ, and one copy of Hydra-RFamide-1. In the transcriptome database from *H. magnipapillata,* we could identify a transcript (named transcript#5) that coded for a preprohormone identical to preprohormone C (Table 4; Additional file 2).

In *H. oligactis*, we identified six Hydra-RFamide preprohormone genes (Table 5; Additional file 2): Gene#1 codes for a preprohormone with one copy of Hydra-RFamide-1, one copy of Hydra-RFamide-3/4, and three additional novel Hydra-RFamide peptides; Gene#2 codes for a preprohormone with one copy of Hydra-RFamide-2, one copy of Hydra-RFamide-3/4 and one additional, novel Hydra-RFamide; Gene#3 codes for a preprohormone, containing three copies of Hydra-RFamide-1, one copy of Hydra-RFamide-2, and four other Hydra-RFamide-like neuropeptide copies; Gene#4 codes for a preprohormone with one copy of the novel neuropeptide pQLMRGRFamide and no further sequences; Gene#5 resembles very much gene#4 and also codes for one copy of pQLMRGRFamide with no additional neuropeptide sequences; Gene#6 codes for one copy of pQWMSGRFamide and no other sequences.

In *H. vulgaris*, we identified eight genes or cDNAs, coding for Hydra-RFamide preprohormones (Table 6, neuropeptide family #2). Gene#1 codes for a preprohormone that is identical to the preprohormone coded for by *H. magnipapillata* Gene#1, with the exception that seven amino residues have been exchanged (highlighted in red font in Additional file 1). These changes are not affecting the neuropeptide sequences contained in the preprohormones (Table 6). We assume that *H. vulgaris* and *H. magnipapillata* might possibly be a single species and that the two genes might be allelic variants.

**Fig. 2:**
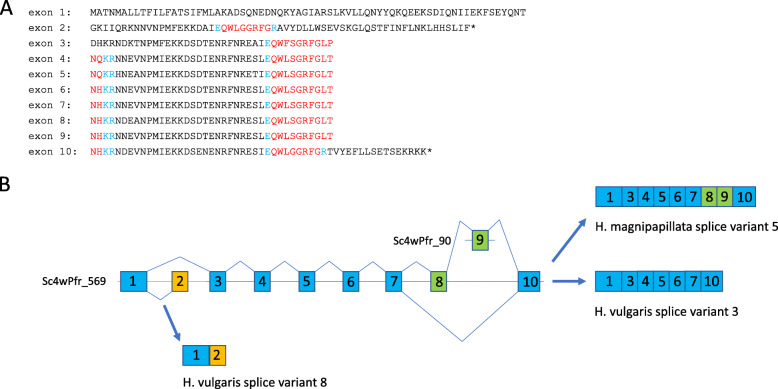
The proposed intron/exon organization of the gene coding for the GRFamide preprohormone-C from *H. magnipapillata* and *H. vulgaris*. **A.** Protein sequences encoded by exons one to ten. The immature neuropeptide sequences are highlighted in red, while the C- and N-terminal cleavage sites are highlighted in blue. Note that the N-terminal peptide cleavage sites are always at the C-terminus of a Glu residue. The gene has ten exons that each code for a fragment of the preprohormone. Exon one codes for the N-terminus of the protein, which also includes the signal sequence. Exons two codes for one copy of Hydra-RFamide-1. Exons three codes for a protein sequence that includes one copy of the non-amidated neuropeptide pQWFSGRFGLX sequence that combines with the XX sequence at the N-terminus of exon four to yield pQWFSGRFGLXXX. Exon four codes for a pQWLSGRFGLX sequence that combines with the XX sequence at the N-terminus of exon five to yield pQWLSGRFGLXXX. This sequence of events continues until exon ten. Exon ten codes for the C-terminus of the protein, which also includes an XX sequence and one copy of Hydra-RFamide-1. **B.** Intron/exon organization. This genomic organization of the preprohormone-C gene is based on the assumption that *H. magnipapillata* and *H. vulgaris* are one species. The genomic organization is supported by our identification of a contig sc4wPfr_569 in the genomic database from *H. magnipapillata*, containing exons one to eight plus exon ten. Exon nine was found on a different contig sc4wPfr_90. We assume that this was due to a technical problem in the genome sequencing or assembly. All exons are flanked by consensus donor and acceptor splice sites. The possibility of alternative splicing is supported by our findings that different transcripts exist of the preprohormone-C gene: *H. magnipapillata* transcript-5 contains exons one to ten. *H. vulgaris* transcript-3 contains exons one, three to seven and exon ten. *H. vulgaris* transcript-8 contains exon one and exon two. Exon two contains a stop codon, which explains the absence of other exon combinations involving this exon

For the other *H. vulgaris* GRFamide preprohormone sequences we also found that they either were identical to the *H. magnipapillata* sequences or that they were possible allelic variants, having 5–9% amino acid residue differences with the *H. magnipapillata* sequence (see Additional file 2 under *H. vulgaris*, where we have aligned the *H. vulgaris* and *H. magnipapillata* GRFamide preprohormone sequences). There were, however, two exceptions, both of them concerning the *H. vulgaris* sequences corresponding to *H. magnipapillata* transcript#5 (Additional file 2). In the first case, we found a large insertion in a *H. vulgaris* preprohormone (coded for by transcript#3) compared to the *H. magnipapillata* preprohormone encoded by transcript#5 (highlighted in red font color in Additional file 2 under *H. vulgaris* Gene#3). In the second case, we found that a *H. vulgaris* GRFamide preprohormone (coded for by transcript#8) lacked a large middle portion compared to the *H. magnipapillata* transcript#5 preprohormone (highlighted in red font color in Additional file 2 under *H. vulgaris* transcript#8). These large “en bloc” insertions and deletions suggested alternative splicing. We, therefore, inspected the genome, where we found that the *H. magnipapillata/H. vulgaris* gene contained ten exons (Fig. 2). Exon one coded for the N-terminus, including the signal sequence, exon two coded for one copy of Hydra-RFamide-1, while exon ten coded for the C-terminus of the preprohormone, including, again, one copy of Hydra-RFamide-1. The other seven exons coded for preprohormone fragments, each containing one copy of pQWLSGRFGLX at its C-terminus and an XX sequence at its N-terminus, which, when combined with its following fragment in the preprohormone, yields a complete pQWLSGRFGLXXX sequence (explained in Fig. 2A). *H. magnipapillata* transcript#5 contained exon one plus exons three to ten (Fig. 2B). *H. vulgaris* transcript#3 contained exon one, exons three to seven, and ten (Fig. 2B). *H. vulgaris* transcript#8 contained exons one and two (Fig. 2B). Exon two is interesting, because it contains a stop codon (Fig. 2A). Therefore, the splice variant containing exons one and two cannot be combined with one of the remaining exons to yield a longer translated protein. Another interesting aspect is that exon nine was found on a contig (Sc4wPfr_90) that was different from the contig (Sc4wPfr_569), where the other exons were located (Fig. 2B). We assume that this was due to a technical artefact that occurred during the genome assembly. As a conclusion, we found that the neuropeptide composition of the preprohormone C protein can be varied with the help of alternative splicing and there may exist many more splice variants than the three examples that we discovered in the present study.

### GLWamide preprohormones in *Hydra*

In 1997, several Japanese and German laboratories published the sequences of isolated GLWamide neuropeptides from *H. magnipapillata* [31]. These peptides were dubbed Hym-53, Hym-54, Hym-248, Hym-249, and Hym-331 (see Table 4 for their structures) [31]. At the same time of that year and independently from the Japanese project, we cloned a cDNA from *H. magnipapillata*, coding for three copies of Hym-331 (see Table 4, Neuropeptide family number 3), one copy of a novel neuropeptide, LPIGLWamide, one copy of a related novel neuropeptide, LPLGLWamide, two copies of Hym-248, one copy of Hym-53, one copy of Hym-54, one copy of Hym-249, one copy of the novel neuropeptide pQPPIGMWamide, and one copy of the novel neuropeptide KPPWRGGMWamide [44]. These last two peptides are not GLWamide peptides sensu stricto, but related to the GLWamide family members. The sequence of the cloned *H. magnipapillata* GLWamide preprohormone is shown in Additional file 3. We analyzed the genomic and transcriptomic databases from *H. magnipapillata* for additional GLWamide preprohormones, but were unable to find more.

*H. oligactis* has a GLWamide preprohormone with a similar overall structure as the preprohormone from *H. magnipapillata* (Additional file 3). Also the neuropeptide sequences are the same, occurring at the same positions in the preprohormone, with the exception of the *H. magnipapillata* GPMTGLWamide sequence, which has been replaced, at the same position with APMPGLWamide in the *H. oligactis* preprohormone (Additional file 3). The various GLWamide peptide sequences in *H. oligactis* are shown in Table 5. We did not find additional GLWamide preprohormones in the databases for *H. oligactis*.

Also *H. vulgaris* has a preprohormone that is similar to the *H. magnipapillata* GLWamide preprohormone (Additional file 3). It contains the same peptides and the same peptide copy numbers as in the *H. magnipapillata* preprohormone (Table 6).

### IFRamide preprohormones in *Hydra*

*Hydra* produces several neuropeptides that are not found in other hydrozoans or cnidarians. These peptides have been isolated from extracts of *H. magnipapillata* during the last three decades mainly by Japanese laboratories. Two such peptides have the sequence IPTGTLIFRamide (dubbed Hydra-FRamide-1) and APGSLLFRamide (dubbed Hydra-FRamide-2) [34]. One common preprohormone for these peptides has also been cloned and it contains one copy of Hydra-FRamide-1 and one copy of Hydra-FRamide-2 [34]. This preprohormone is shown in Additional file 6. Its sequence shows that it also could give rise to an N-terminally elongated form of Hydra-FRamide-2, pQGAPGSLLFRamide, which is N-terminally protected, but which was not isolated by the Japanese group [34] (Table 4). When we inspected the *H. magnipapillata* genome sequence, we could not find additional genes, coding for the Hydra-FRamides.

*H. oligactis* has a preprohormone that is similar to the *H. magnipapillata* preprohormone, but clearly shorter. It contains one copy of Hydra-FRamide-1 and one copy of N-terminally elongated Hydra-FRamide-2, pQGAPGSLLFRamide (Table 5; Additional file 6).

*H. vulgaris* contains a Hydra-FRamide preprohormone that is identical to the preprohormone from *H. magnipapillata* (Table 5; Additional file 6). We did not find additional Hydra-FRamide preprohormone sequences in the *H. vulgaris* database.

We were also unable to find Hydra-FRamide preprohormone sequences in other cnidarians, like hydrozoans, scyphozoans, staurozoans, cubomedusae, and anthozoans [52, 53].

### PKVamide preprohormones in *Hydra*

Another neuropeptide that is present in *Hydra*, but not in other cnidarians, has the sequence APFIFPGPKVamide (Table 4, neuropeptide family #7). It was isolated from extracts of *H. magnipapillata* and dubbed Hym-176 [32]. Its cDNA was also cloned, which showed that it coded for a preprohormone with a single copy of the Hym-176 [67]. The sequence of this Hym-176 preprohormone from *H. magnipapillata* is shown in Additional file 7. The researchers behind this work claimed that a second neuropeptide should be present in the C-terminal part of the Hym-176 preprohormone with the mature sequence KPAFLFKGYKPamide and the immature sequence KPAFLFKGYKPGD [67] (Additional file 7). However, the immature sequence does not have any of the canonical, C-terminal GKR, GRR, GK, or GR sequences needed in cnidarian preprohormone processing by PC1/3 [6, 7, 46, 47]. We, therefore, assume that this second neuropeptide does not exist.

*H. oligactis* has two genes coding for the Hym-176 preprohormone (Additional file 7): Gene#1 codes for a preprohormone, having one copy of Hym-176; Gene#2 codes for a novel neuropeptide with the sequence pQGPKVamide, which is a shorter, C-terminal version of Hym-176 (Table 5).

In *H. vulgaris*, we found three genes encoding a Hym-176 preprohormone: Gene#1 codes for a preprohormone that is identical to the *H. magnipapillata* preprohormone (Additional file 7). Gene#2 appears to be an allelic variation of Gene#1: Its preprohormone contains one amino acid insertion (highlighted in blue in Additional file 7) and seven amino acid exchanges (highlighted in red in Additional file 7), which, however, do not affect the sequence of Hym-176 neuropeptide. Gene#3, however, codes for a novel preprohormone, containing one copy of pQGPKVamide, which is identical to the short Hym-176 version found in *H. oligactis*, and another novel neuropeptide pQSPSNKVamide (Table 5, neuropeptide family 7; Additional file 7).

### Neuropeptide preprohormones in Endocnidozoa

Endocnidozoans are strongly reduced endoparasites that have recently been linked to the subphylum Medusozoa [60, 68] (Fig. 1). We tested the genomes and transcriptomes from nine endocnidozoan species (Table 7) for the presence of neuropeptide preprohormone genes and found that two species expressed these genes. In *P. hydriforme*, we discovered two genes, coding for GRFamide preprohormones. Gene#1 likely codes for two preprohormone fragments, one containing the N-terminus and the other one containing the C-terminus of an incomplete preprohormone (Additional file 2). These fragments contained altogether three copies of the neuropeptide pQWLRGRFamide and one incomplete pQWLRGR sequence (Table 3; Additional file 2). The three pQWLRGRFamide neuropeptide sequences are identical to other GRFamide sequences from hydrozoans such as those from *C. sowerbii*, *V. vellela*, and *P. porpita* (Tables 2 and 3). Gene#2 codes for the C-terminus of a preprohormone, containing two copies of pQWIKGRFamide (Additional file 2). These sequences are, again, strongly resembling the GRFamide sequences from other hydrozoans, for example those from *P. physalis* (86% amino acid identities between the two peptides, see Table 2). *P. hydriforme* also has a gene coding for a short, but complete preprohormone, containing one copy of YPPGLWamide (Table 3, Additional file 3). This peptide resembles other GLWamide peptides from other hydrozoans, such as those from *P. physalis* and *M. alcicornis* (83% amino acid residue identities; Table 2, Table 3).

**Table 7:** Accession numbers for the different endocnidozoan databases used

Species	Class	Subclass	Database type	Accession numbers and reference (if published)
*Buddenbrockia plumatellae*	Myxozoa	Malacosporea	EST	(ES599040.1 – ES599804.1) [69]
*Enteromyxum leei*	Myxozoa	Myxosporea	WGS	LDNA00000000.1 [60]
*Henneguya salminicola*	Myxozoa	Myxosporea	TSA WGS	GHBP00000000.1 SGJC00000000.1
*Kudoa iwatai*	Myxozoa	Myxosporea	TSA WGS	GBGI00000000.1 [60] JRUX00000000.1 [60]
*Myxobolus cerebralis*	Myxozoa	Myxosporea	TSA	GBKL00000000.1 [60]
*Myxobolus squamalis*	Myxozoa	Myxosporea	TSA WGS	GHBR00000000.1 QWKW00000000.1
*Sphaeromyxa zaharoni*	Myxozoa	Myxosporea	WGS	LSMZ00000000.1
*Thelohanellus kitauei*	Myxozoa	Myxosporea	WGS	JWZT00000000.1 [70]
*Polypodium hydriforme*	Polypodiozoa	Polypodiidea	TSA	GBGH00000000.1 [71]

The endocnidozoan *Buddenbrockia plumatellae* contains one gene, coding for an incomplete preprohormone with six copies of pQWARGRYamide (Additional file 2). These peptides are belonging to the GRFamide peptide family (see Table 3). The pQWARGRYamide preprohormone could only be detected in the *B. plumatellae* database and not in the databases from its hosts (bryozoans and trout), showing that our findings are not due to database contaminations.

## Discussion

One of the goals of the current study was to establish which neuropeptides were present in the common ancestor of cnidarians. Previously, we analyzed altogether fourty-two species with sequenced genomes and/or transcriptomes, belonging to the classes Cubozoa, Scyphozoa, Staurozoa, and Anthozoa, for the presence of any known and unknown neuropeptide preprohormones [51–53]. These bioinformatics analyses suggested that three neuropeptide families must have evolved early in cnidarian evolution: The X_1_PRX_2_amides, the GRFamides, and the GLWamide peptides (Fig. 1). However, one cnidarian class, the Hydrozoa and one subphylum, the Endocnidozoa, were lacking in our analyses and remained to be investigated. In our present study, therefore, we analyzed sixteen species, belonging to the Hydrozoa (Table 1) and nine species belonging to the Endocnidozoa (Table 7). These analyses showed that the X_1_PRX_2_amide, the GRFamide, and the GLWamide genes occurred in all hydrozoans. Some members of the Endocnidozoa also expressed GRFamide and GLWamide genes, but we were unable to find X_1_PRX_2_amide genes (Fig. 1). Endocnidozoa have some of the smallest genomes in the Animal Kingdom [60, 68], due to massive gene loss. This might explain the absence of X_1_PRX_2_amide genes in these animals. Based on the phylogenetic positions of the seven cnidarian classes or subphyla (Fig. 1), however, we can safely conclude that the X_1_PRX_2_amide, the GRFamide, and the GLWamide genes must have been present in the common cnidarian ancestor.

Placozoans are a sister phylum to cnidarians and also produce peptides from preprohormones that display the same unusual characteristics as cnidarian preprohormones, i.e., with classical basic prohormone convertase cleavage sites positioned at the C-termini of their immature peptide sequences, and unconventional “cnidarian-type” cleavage sites at their N-termini, which are often acidic residues [6, 9, 72]. These preprohormone characteristics are only known to occur in Placozoa and Cnidaria, which supports an earlier conclusion, based on phylogenomics, about the close phylogenetic relationships between these two phyla [73]. Our preprohormone data also indicate that “cnidarian-type” preprohormones must have been present in the common ancestor to cnidarians and placozoans. Interestingly, placozoans do not have a nervous system, but, instead, endocrine cells that are producing the various peptides [10, 72]. We do not know, whether the common ancestor of cnidarians and placozoans had nerve cells, endocrine cells, or both. Therefore, we cannot draw conclusions about the first peptidergic cells that arose during evolution.

Although placozoans produce “cnidarian-type” preprohormones, their mature peptide sequences do not resemble cnidarian neuropeptide sequences; for example none of them has sequences similar to the X_1_PRX_2_amide, GRFamide, and GLWamide neuropeptides. This finding is somewhat surprising, but could imply that the common cnidarian ancestor and the common ancestor for both cnidarians and placozoans were separated by a considerably long evolutionary distance. We know from our own work on protostome invertebrates that, during evolution, neuropeptide ligands may swiftly change their structures to such a degree, that it is hard to recognize evolutionary relationships [74, 75]. GPCRs, in contrast, are more conserved and knowing the neuropeptide GPCRs may help to resolve neuropeptide evolution in early evolved metazoans [74, 75].

Endocnidozoans are microscopically small endoparasites with a strongly simplified morphology, often consisting of just a handful of cells with polar capsules that resemble cnidarian nematocytes (stinging cells), which are used by the parasites for host adherence [60, 68, 76–78]. Most of these parasites have complex life cycles, where they alternate between an invertebrate and a vertebrate host, the last one frequently being a fish [60, 68, 76, 77]. For long, the phylogenetic position of endocnidozoans has been a mystery, but in the last few years it became obvious that they constitute a subphylum of the Cnidaria, clearly related to the cnidarian subphylum Medusozoa (Fig. 1) [60, 68, 78]. Endocnidozoans consist of two taxa: The highly diverse Myxozoa, comprising of about 2500 species, and its sister taxon Polypodiozoa that, so far, only consists of one species, *P. hydriforme* [60, 68, 71, 77, 78].

The life cycle of *P. hydriforme* starts as a binucleate cell that intracellularly parasitizes oocytes in the ovaries of sturgeons and other basal, ray-finned fishes [76]. Within this oocyte, the parasite undergoes its embryonic development, feeding on the yolk of its host cell, after which it becomes an intracellular planula larva and finally develops into a stolon (an elongated, tube-like structure) that is released into the water during spawning of its female fish host. Starting from this stage, *P. hydriforme* is free-living. Its stolon fragmentizes and produces numerous polyp-like individuals that develop tentacles and a mouth and that are able to move (“walk on their tentacles”) and feed [76]. These individuals multiply by longitudinal fission and later, during the summer, produce gonads that, packed in a kind of “gonadophore” and equipped with polar bodies, are released into the water. These “gonadophores” adhere to a new female fish host, enabling *P. hydriforme* to infect her and start its life cycle again [76]. *H. hydriforme* is an atypical endocnidozoan, because it has a long and active free-living period in its life-cycle, and does not use an invertebrate host.

There exists no genome, but only one transcriptome database from *P. hydriforme*, which was constructed from free-living stolons 24-h after their release from the oocytes (Table 7) [71]. In this stolon database we identified three incomplete cDNA fragments, coding for three GRFamide preprohormones fragments (Additional file 2). Two of these incomplete preprohormones probably constitute the N- and C-termini of a common preprohormone, while the third fragment represents a different GRFamide preprohormone (Additional file 2). All three preprohormone fragments show the typical characteristics of a “cnidarian-type” preprohormone, confirming again that *P. hydriforme* is a derived cnidarian [76]. The first two preprohormone fragments (encoded by gene#1) contain altogether three copies of the neuropeptide pQWLRGRFamide (Additional file 2, Table 3). These neuropeptides are identical to those found in several other hydrozoans: For example, *C. sowerbii* has four genes producing altogether nine copies of pQWLRGRFamide; *P. porpita* has one gene containing ten copies of this peptide; and in *V. vellela* we found two incomplete cDNAs coding for six copies of this peptide (Additional file 2). These findings would suggest a close relationship between *P. hydriforme* and Hydrozoa. However, other medusazoans, like cubozoans and scyphozoans, also produce preprohormones with very high copy numbers of pQWLRGRFamide, which can even be thirty-two copies per preprohormone, such as in the scyphozoan *Rhopilema esculentum* [52]. Staurozoans, octocorals, hexacorals, and ceriantharians produce different GRFamide peptides, which are often much shorter [52, 53]. Therefore, it seems that *P. hydriforme* is most closely related to a cnidarian clade formed by hydrozoans, scyphozoans and cubozoans (Fig. 1).

The existence of GRFamide preprohormone cDNA in a transcriptomic database of one-day old stolons from *P. hydriforme*, confirms a previous publication by Raikova et al. [59], which shows the presence of an ectodermal, FMRFamide-immunoreactive nerve net and its underlying muscle sheet in the free-living “walking” polyp stage of the parasite. The combined findings by us and Raikova [59], concerning these two different stages, suggest that GRFamides are expressed during the complete free-living period of *P. hydriforme*.

One day-old stolons from *P. hydriforme* also produce a GLWamide preprohormone (Additional file 3). This preprohormone contains one copy of the neuropeptide YPPGLWamide and is unusually short, but it has, again, all the characteristics of a “cnidarian-type” preprohormone (Table 3, Additional file 3). It would be worthwhile to raise antibodies against this novel *H. hydriforme* neuropeptide and determine where and when it is expressed. These studies together with antibody studies locating the pQWLRGRFamide neuropeptide from *P. hydriforme* could give important information on the anatomy of the nervous systems in the different stages of the parasite. They could also yield clues about the functions of these peptides, which would certainly help to better understand *P. hydriforme*.

We were unable to identify X_1_PRX_2_amide preprohormones in *P. hydriforme*. X_1_PRX_2_amides were recently identified in *C. hemisphaerica* as neuropeptides that induce oocyte maturation and spawning in sexually mature hydromedusae [35, 79, 80]. Thus, one explanation for our failure to find X_1_PRX_2_amides in *P. hydriforme* could be that one-day old stolons do not express these peptides and that we need to test sexually mature stages of this parasite.

The Myxozoa are subdivided into two subclasses: The strongly reduced Myxosporea and the anatomically less simplified Malacosporea. The myxosporeans are species-rich, while Malacosporea has only about 20 species, belonging to two genera *Tetracapsuloides* and *Buddenbrockia* [81]. *Buddenbrockia plumatellae* has been well-studied. This parasite has an active worm-like (vermiform) life stage, while parasitizing and developing within the body cavity of its invertebrate (bryozoan) host [69, 82]. The *Buddenbrockia* worm displays vigorous, sinuous writhing, but apparently lacks a nervous system, external sense organs, and a gut [69]. Also other more recent anatomical studies on the different vermiform stages of *B. plumatella* have revealed its muscular development in great detail, but no indications for the presence of a nervous system in this animal [83]. In our current study, however, we find that *B. plumatella* expresses a GRFamide preprohormone, which produces six copies of the neuropeptide pQWARGRYamide (Table 3; Additional file 2), indicating that the parasite must have a nervous system. It would be worthwhile to raise antibodies against pQWARGRYamide and use them for staining of the different vermiform stages of *B. plumatella* to uncover, for the first time, the neuroanatomy of a myxozoan. These experiments will probably also explain why the worm is able to contract its muscles. Several species belonging to the genera *Tetracapsuloides* and *Buddenbrockia* have vermiform stages, while parasitizing their invertebrate hosts [84, 85], and it is likely that all of them express pQWARGRYamide preprohormones. Furthermore, these malacosporeans also parasitize a wide variety of wild and aqua-cultured fish species, where they cause Proliferative Kidney Disease (PKD), which kills these fishes. Therefore, any new knowledge on these parasites might eventually lead to a method for preventing PKD.

The pQWARGRYamide preprohormone (Additional file 2) has all the characteristics of a “cnidarian-type” preprohormone, clearly linking *B. plumatella* to cnidarians. However, the pQWARGRYamide neuropeptide sequence itself is quite derived and does not closely resemble any of the other cnidarian GRFamide neuropeptide sequences. Yet, it has 72% amino acid residue identity with the GRFamide peptides from *C. sowerbii* (pQWLRGRFamide; Table 3), indicating that it still belongs to the hydrozoan/medusozoan GRFamide neuropeptide family. The derived structure of pQWARGRYamide probably reflects the long evolutionary distance between *B. plumatella* and its extant medusozoan relatives.

We were unable to find X_1_PRX_2_amide and GLWamide peptides in *B. plumatella.* The reasons for this failure may be manifold, but one reason could be that these peptides were not expressed during the specific vermiform stages, from which the EST database was constructed (Table 7) [69, 83].

The majority of the databases that we used for testing the remaining myxosporeans are whole genome sequence databases (Table 7). Here, we failed to find any neuropeptide genes. Provided, of course, that the genome databases are of sufficient qualities (Table 7), these results mean that the genomes from most myxosporeans have lost their neuropeptide genes, due to extreme genome reductions in connection with their parasitic life.

All hydrozoans have genes, coding for the three primordial neuropeptide families: The X_1_PRX_2_amides, GRFamides, and GLWamides (Fig. 1). In many cases, hydrozoan species contain single genes, each coding for one of the three neuropeptide families. Such situations give simple neuropeptide expression patterns, for example in *D. pumila*, *P. porpita*, *V. vellela*, *M. alcornis*, and *M. squarrosa* (Table 2). These findings suggest that the presence of one X_1_PRX_2_amide, one GRFamide, and one GLWamide gene is sufficient for a hydrozoan animal to develop, disperse, feed, and reproduce. Several other hydrozoans, however, have duplicated one or more of these basal genes and, thereby, extended their hydrozoan neuropeptide repertoire. One extreme example is *C. hemisphaerica*, which contains five genes, coding for X_1_PRX_2_amide peptides (Table 3; Additional file 1). X_1_PRX_2_amides in *C. hemisphaerica* are responsible for oocyte maturation and oocyte release [35, 79, 80], but it is unclear to us, why five different genes would be needed for this process. However, we have recently also identified X_1_PRX_2_amide peptides in a transcriptome from the cubomedusa *Tripedalia cystophora* and raised antibodies against them [51, 52, 86]. Immunocytochemical staining of non-sexual medusae from *T. cystophora*, using these antibodies, revealed a specific set of giant and sensory neurons in their rhopalia, which is at a location, where ovaria do not occur [86]. Therefore, X_1_PRX_2_amide genes must have other roles than the ones related to reproduction and this might also be the case in *C. hemisphaerica*.

Another extreme example is the freshwater hydromedusa *C. sowerbii*, which has multiplicated all of its primordial neuropeptide genes, thereby having: Two X_1_PRX_2_amide preprohormone genes, six GRFamide preprohormone genes, and three GLWamide preprohormone genes. Furthermore, it has created a novel gene coding for a preprohormone pQFLRPamide (two copies) and pQFIRPamide (one copy) (Table 3; Additional file 1 to Additional file 4). This abundant peptidergic signaling in *C. sowerbii* is surprising and we do not understand the reasons for it.

A third example is *H. vulgaris*, which has no less than eight different genes or transcripts coding for GRFamide preprohormones, two genes for X_1_PRX_2_amides, one gene for GLWamides, one gene for a LFPamide preprohormone, and three genes coding a PKVamide preprohormone (Table 6; Additional files 1-3, 6, and 7). Thus, *H. vulgaris* has not only strongly multiplicated its GRFamide genes, but also created novel genes, in addition to its primordial genes. Again, we do not understand why *H. vulgaris* and the other *Hydra* species, which all have simple behavioral repertoires, need to have such complex neuropeptide signaling. Yet, neuropeptide signaling might also be involved in other biological processes, such as development and water homeostasis [30, 33, 38, 39, 87].

Another interesting phenomenon that we observed in *Hydra* was alternative splicing of the *Hydra* preprohormone-C gene, giving rise to at least three different GRFamide transcripts (Fig. 2). This alternative splicing of a neuropeptide gene transcript further increases the complexity of neuronal signaling in *Hydra*.

In conclusion, we have found that all hydrozoans express at least one copy of an X_1_PRX_2_amide, GRFamide, and GLWamide gene, which appears to be sufficient for a substantial number of hydrozoans to live a normal hydrozoan life. In the course of evolution, however, some hydrozoans have multiplied these primordial neuropeptide genes and, in addition, developed new genes and alternative splicing of neuropeptide gene transcripts. These new developments have increased the complexity of neuronal signaling, a process, which must be related to a more complex behavior or a better regulated physiology of these animals.

## Methods

### Sequence data

Table 1 and Table 7 give an overview of the databases used in this paper, including the accession numbers used for downloading them from GenBank (https://www.ncbi.nlm.nih.gov/genbank/). For the analyses we downloaded all hydrozoan and endocnidozoan genomes and TSAs available from NCBI in September 2020**.** For the TSAs we used the following search terms: ‘tsa-master [key] hydrozoa’ and retrieved the corresponding FASTA-files. The data for *C. hemisphaerica* were downloaded from http://marimba.obs-vlfr.fr/organism/Clytia/hemisphaerica in September 2020.

### Identification of neuropeptide preprohormones

We used a dual approach with TBLASTN and a script based on preprohormone characteristics for identifying preprohormones. The script has been described and tested in [51–53, 72]. It was based on the identification of genes coding for proteins containing both a signal sequence and multiple prohormone processing motifs (GKK, GKR, GR). The program takes as inputs FASTA files from genomes and TSAs after they are translated and split into open reading frames (ORFs). Each of these ORFs are then searched for neuropeptide processing sites (GKR, GKK and GR). The ORFs with three or more of these processing sites are then further analyzed. Cnidarian neuropeptide preprohormones are generally repetitive with highly similar mature peptides, in particular the C-terminal segment of the peptides. In the downstream analysis, the five amino acid residues preceding the processing sites are retrieved and only those ORFs, where at least two peptides share four out of five amino acids, are kept for subsequent analysis. The python script is available on https://github.com/Thomaslundkoch/neuropeptide/blob/master/neuropeptide_finder.py, where the following parameters were used: motifs: [‘GR’, ‘GKR’, ‘GKK’]; proccessing_site_threshold: 3; peptide_length: 5; identity_threshold: 80. At this stage, the presence of a signal sequence was assessed by Signalp 5.0 (http://www.cbs.dtu.dk/services/SignalP/) and only the sequences with a signal sequence were considered as putative neuropeptide precursors. These candidates were manually curated based on the presence of cnidarian neuropeptide preprohormone hallmarks: Signal sequence, canonical C-terminal processing sites, a high level of similarity among the mature peptides, and the presence of N-terminal protection groups in the mature peptides (pGlu, XP, or XPP sequences).

As our script relies on the presence of multiple processing sites in the preprohormone, it will not be able to identify precursors with only one or two processing sites (the setting of minimally three processing sites in the programs was chosen to reduce the number of false positives). To overcome this limitation, we also used online TBLASTN with a very large collection of known bilaterian preprohormones as queries (downloaded from uniprot with the following search terms: ‘goa:(“neuropeptide hormone activity [5184]”) AND reviewed:yes’ on September 2020 (Additional file 8). We also used published cnidarian and placozoan preprohormones and cnidarian and placozoan neuropeptide sequences [6, 9, 20, 22–35, 40–45, 52, 53, 67] as queries. TBLASTN was performed with expected threshold 0.1 and word size 2. Thus, TBLASTN enabled us to identify neuropeptide preprohormones with just one or two neuropeptide copies.

## Supplementary Information

**Additional file 1.** Partial or complete amino acid sequences of the X_1_PRX_2_amide preprohormones in species belonging to the Hydrozoa.

**Additional file 2.** Partial or complete amino acid sequences of the GRFamide preprohormones in species belonging to the Hydrozoa (Part One) or the Endocnidozoa (Part Two).

**Additional file 3.** Partial or complete amino acid sequences of the GLWamide preprohormones in species belonging to the Hydrozoa (Part One) or the Endocnidozoa (Part Two).

**Additional file 4. **Partial amino acid sequence of the LRPamide preprohormone from the hydrozoan *Craspedacusta sowerbii* (neuropeptide family 4).

**Additional file 5. **Partial amino acid sequence of two RWGamide preprohormones from the hydrozoan *Clytia hemisphaerica* (neuropeptide family 5).

**Additional file 6. **Complete amino acid sequences of the LFRamide (neuropeptide family 6) preprohormones from three *Hydra* species.

**Additional file 7. **Complete amino acid sequences of the PKVamide neuropeptide (family 7) preprohormones from three *Hydra* species.

**Additional file 8.** FASTA files used for TBLASTN.

## Data Availability

All protein sequences from Additional files 1, 2, 3, 4, 5, 6, and 7 have been retrieved from publicly available genomic and transcriptomic databases (see Table 1 and Table 7). When publications were associated with these databases, they were given in Table 1 as [61–65], and in Table 7 as [60, 69–71].

## Abbreviations

A-RFamide: Antho-RFamide
GPCR: G protein-coupled receptor
HFR: Hydra-FRamide
HRF: Hydra-RFamide
H53: Hym-53
H54: Hym-54
H176: Hym-176
H248: Hym-248
H249: Hym-249
H331: Hym-331
H355: Hym-355
PC: Prohormone Convertase
PKD: Proliferative Kidney Disease
pQ: Pyroglutamate residue
trs: Transcript
