# Supplementary material for: An evolutionary genomics view on neuropeptide genes in Hydrozoa and Endocnidozoa (Myxozoa)
Source: BMC Genomics. 2021 Nov 30;22:862. doi: 10.1186/s12864-021-08091-2 (PMC8638164; doi:10.1186/s12864-021-08091-2)
Supplement: Supplementary file 1 — Additional file 1. Partial or complete amino acid sequences of the X1PRX2amide preprohormones in species belonging to the Hydrozoa. [file 12864_2021_8091_MOESM1_ESM.pdf]

Additional file 1. Partial or complete amino acid sequences of the X_1PRX_2amide preprohormones in species belonging to the Hydrozoa. For some species more than one preprohormone fragment was identified, which sometimes indicated the presence of more than one gene or the presence of splicing variants. Signal sequences are underlined. An asterisk indicates a stop codon. Neuropeptide sequences are highlighted in yellow; C-terminal processing sites are highlighted in green. The C-terminal Gly residues that are converted into C-terminal amide groups are highlighted in red.

Clytia hemisphaerica

Gene 1

>TCONS_00015710-protein
MLSSETTIRILCFFIAVGFAVGSSSPEEEGQLLHVKRETWLNPGFDSMLHRRESQELLNRPRPGRRELFDLMN
QDSILKKRALLHRPRPGRRELFRNPGLDSMLHKRGQEFLSGPRPGREIRPRPGRRERHPDSMLHRRSSDTDLD
YQHLLRNPRPGRRELFRPRPGRRELQHPDSLLHRRSEEMWSRPRPGKREVYYENDGRSEDEKLLRVLDELKRD
IIDELWDRFQN*

The below preprohormone was recently identified in Clytia hemisphaerica [35] and dubbed Che-pp8. It is similar to the above preprohormone coded for by gene#1, but has an insertion of about 25 amino acid residues (highlighted in red font color).
MLSSETTIRIFCFFIAVGFAVGSSSPEEEGQLLHVKRETWLNPGFDSMLHRRESQELLNRPRPGRRELFDLMN
QDSILKKRALLHRPRPGRRELFRNQGLDSMLHKRAMLNRPRPGRRELFRNPGLDSMLHKRGQEFLSGPRPGRE
IRPRPGRRERHPDSMLHRRSSDTDLDYQHLLRNPRPGRRELFRPRPGHRELQHLDSLLHRRSEEMWGRPRPGK
REVYYEDDGRSEDEKLLRVLDELKRDIIDELWDRFQN

Gene 2

>TCONS_00006521-protein
MNCVLVFLVLFLANNVYSASLTREEDALVTKLLDTIEKRDAVPRLGKREVPRLGREIEEVPRLGREIELVPRL
GREAEEVPRLGREVELVPRLGREVEVPRLGREVPRLGREMEEVPRLGREIDEVPRLGREVPRLGRELEVPRLG
REVPRLGKREIPRLGKREVPRLGREASTYDLKQLYNQLKREVSNDMIEAEIKEEKRVLKAFNLGTRGLILRRI
GDKLNKDGFSDQKRGMNEKESSRPFLHVKRTNLLSLIEKLTSEE*

The below preprohormone was identified in Clytia hemisphaerica and dubbed Che-pp3 [35]. It resembles the gene#2 gene product, but has several insertions (highlighted in blue font color), exchanges (highlighted in red) and deletions.
MNCVLIFLVLFLANNVYSASLTREEDALVTKLLDTIEKRDAVPRLGKREVPRLGREIEDVPRLGREIELVPRL
GREAEEVPRLGREAEEVPRLGREVELVPRLGREVELVPRLGREVEVPRLGREVPRLGRGIDEVPRLGREVPRL
GRELEVPRLGREVPRLGKREIPRLGKREVPRLGRREVPRLGREASTYDLKQLYNQLKREVSNDMIEAEIKEEK
RVLKAFDLGTRGLILRRIGDKLNKDGFSDQKRDMNEKESSRPFLHVKRTNLLSLIEKLTSEE

Gene 3

>TCONS_00068562-protein

MERKFLACLFLLIVLLNLNDGKNIAILIEPDDNLASELEWLGSDMTDSHSLNAGAWPRPGDARSSHDAWPRPG
KREFYGNEMFEKRPFPGQQMQFSWPRPGKKETKEDTWPRPGKRESYSEGDMDSRSGALRRSEEKETNEDEKLE
NAWPRPGKREFYASRKMDVRPRGGRDSKSHKISKRNSEAISYDEIDMMLREEAWPRPGKRDYHMLSVTRPRGG
KDARPRGGKDSLRPRGGKDAKPRGGKDSVRPRGGKKDSWPRPGKDAFVNEINGSRPRGGKDASKWPRPGKKDI
K*

The below preprohormone was recently published in [35] and dubbed Che-pp1. It resembles the gene#3 gene product, but has several amino acid exchanges (highlighted in red).

MERKILACLFLLIVLLNLNDGKNIAILIEPDDNLASELEWLGSDMTDSHSLNAGAWPRPGDARSSHDAWPRPG
KREFYGNEMFEKRPFPGQQMQFSWPRPGKKETKEDTWPRPGKRESYSEGDMDSRSGALRRSEEKETNEDEKLE
NAWPRPGKREFYASRKMDVRPRGGRDSKSHKISKRNSEAISNDEIDMMIREEAWPRPGKRDYHMLSATRPRGG
DARPRGGKDSSRPRGGKNAKPRGGKDSVRPRGGKKDSWPRPGKDAFVKEINGSRPRGGKDASKWPRPGKKDLK

Gene 4

>SC0002552

MNLLVSILVFICAIFLKLTESAPISNVRKIGNNELLLKLTISDLAKLLSRLQNVHEDGQKEALNKGSMQDTIV
DYLDEKQNKDRPRYGKDLKETFRPRYGKEMSEGENQNVIERLIEKLVNQSSESDTKDDGNIKSDGKVDNLVSL
LHGLDEEKEWPRPGKDWPRAGKDWPRAGKDWPRAGKDWPRAGKDWPRPGKDQPNGIARGGKRSLGEILSDLMS
KRPRYGKDESSTNGPSYQYTLEDMLTEIISGDRPRYGRKSEASRPRYGKESADKTLEEALSNLGTKSTKKDTS
SRSSLARGGKKRGVENLILEYIENHLDDKKDYESARKDTPK*

The preprohormone given below was published in [35] and dubbed Che-pp4. It resembles the gene#4 product. However, there are many amino acid residue exchanges, deletions and insertions in Che-pp4 compared to the gene#4 product.

MNLLVSIPVICAIVLKLTESAPISNVRKIGSNELLLKLTVSDLAKLLSRLQNVHEDGHKEDLNKVSVEGMIAD
YLDEKQYKDRPRYGKDLKEASRPRYGKEMSEGGNHNVIEQLIEKLVNQSSESDTKDDGNIKSDGKVDNLVSLL
HGLDEEKEWPRPGKDWPRAGKDWPRAGKDWPRAGKDWPRAGKDWPRAGKDWPRPGKDQPNGIARGGKRSLGEI
LSDLMSKRPRYGKDESSTNGPSYQYTLEDMMTEIISGDRPRYGRKSEASRPRYGKESADKTLEEALSNLGTKS
TKKDTSSRSSLARGGKKRGVEDLILEYIENHLDDKKDYESARKDTPK

Gene 5

This sequence has been identified in the current paper.

>TCONS_00015647-protein

MRLVNVVLTILTSKIALGEGRPPSRESDIDQQLIRLAHEIRREIQPGPFGNYRGYAPPRLGKRENIDGKKGVT
SQKRRTVEALDKIGQILLNHRKSLSSDSEVFKNGKKMMTDHLDTTSRKRTHHFIKAKNVPFWYFNKRGLSNQV
DGKKTLGDGNKPAFTHHSQPTFDKFADDVIEKLTTLNKQIKHDAINERDEKMKKRERVGMKSKWNFWPPRLGR
KRETTLNRNMEKAKHPYDRILDDKKGL*

Craspedacusta sowerbii

Gene 1

>QQSS01000183.1 Craspedacusta sowerbii isolate 2016 scaffold743_cov27, whole genome shotgun sequence

MNFIYAFVFIIVTLASLEDAYKLADLPGKEEHLPGSKLKIAFEEVLSNSVDESPTEEDSSINSVPQGEEYVVT
IEAIPVEETSTVGSNEVEEDLFDPEKENFETFEEEDGDEAKRRRSNGRSAEDYLREILVALENGEADAPRAGR
DSHAPRAGRKKSTDYKRTSHPWGPRQDGDSRFESAGLAFPAPRAGRELGPPLSGRRILGRAVDKERTAPRAGR
RSEAPRAGRETSAPRAGRALAPRAGRENKAPRAGRDIIEALGVSREMNAPRAGRRDLEAPRAGRGFLARRADR
RTITAPRAGRETLEAPRAGRETIEAPRAGRETIEAPRAGREILSAPRAGRELMDAPRAGRENLANLQRERRDN
NEKHEVAVSKVNSAAEAPRAGREDDFGKSASSSSEVARHEMRSSNLQAEKHREIAEAAASEEAAEELIDEEEG
EANEDR*

Gene 2

>QQSS01051208.1 Craspedacusta sowerbii isolate 2016 scaffold213654_cov52, whole genome shotgun sequence

MNCFYAVVCIFVTSASLEHAYKLADPPGKEEHFSGPKLKIAFEEVLSNSLDETPTGEDSSLNSVPQSEEYVVT
IEAIPVEDASAEGNNEVEEDLLDPERENFETFEEEDSDEAKRRRSAGRNSDDYLREILVALENGEADAPRAGR
DSYAPRAGRKKSADYKRTSHPWGPRQDSDNRFERVGLAFPAPRAGRELGPPLSGRRILGRAMDRERTAPRAGR
DVIAPRAGRRSEAPRAGRETSAPRAGRVVAPRAGRENKAPRAGRDIIEALRASREMNAPRAGRRDIGAPRAGR
DFFAPRAGRRAIRAPRAGREAIEAPRAGRETIEAPRAGREILAAPRAGREVIDAPRAGRENLADLGRERRDTK
KKHEVTVSKVDSAFDAPRAGREEEFGKSASVLSDVGLHELRSSNSQSEKHRELAEAATSEEAAEELIDEEEGE
AEADR*

Dynamena pumila

>GHMC01005391.1 TSA: DYNAMENA PUMILA ISOLATE DYNAMENA PMILA COLONY DYNAMENA5390, TRANSCRIBED RNA SEQUENCE

MFFQITGIVCILCCTNVLAGPTGGDDKLYKLLDLFQKRTADAPAFLRWPRPGREIDDEFPRGGKRELPVRVGK
RESPVRVGKRELPVRVGKRELPVRVGKRDTTDLSIDNLVNLYDQIKREAMNAVIERLVEKRADTQEAFLHKLK
DEEDIKSVEKRQSAFLHKKEKGDTLKELLAQLKNVR

Hydractinia symbiolongicarpus

Gene 1

>GCHW01018914.1 TSA: Hydractinia symbiolongicarpus Hydractinia_32215.0_Transcript_1/0_Confidence_1_Length_848 transcribed RNA sequence

MRALIIVVVIIGSTFLKGGDATSEGSYEKISSSLLKYLPDAFFEEASEYLKEEREATHMYNPLKLALSRPRGG
KRDELSNLLWYRPRAGRSLSEKSDNYASRPRAGKRESRYETKRNLYRPRAGKKDMLDERNKMSDFRPRAGKRF
VKVNSVESVEKKNLQHTQRSLGYNEYEDRRYSNEREADGSDFKNKNVGSRYYAD*

Gene 2

>GAWH01026881.1 TSA: Hydractinia symbiolongicarpus Hs_transcript_26883 transcribed RNA sequence

MRVLLHLLIILTCFYHVLQAYRRDVKTILDQDENGPAVEDELNYKTIRSLPDPQPRGGRGGRSGRRYRRGGGS
GGVCCVVDLTPGEIIGIIFGSVFGLAFLLTLLTYCCEYCKRRANRPTKLWHVGPTYSERMEMMKKRKQERQPL
ECS*

Hydra magnipapillata

The cDNA of Gene 1 has been cloned earlier see ref. [33].

Gene 1

>GAOL01004674.1selectiontranslationframe-3

MLSLTVATLLLITSIVMAMPNRDATDSNESDILNILDEYIVKVAEMTANEAKILNDVRNYYNDRSSKSLGEFP
QSFLPRGGKRDARPRAGK*

Gene 2

>GAOL01011830.1selectiontranslationframe+1

MMRTAVFGCFILFTIVLALPYRDAFNLFDRFDEYIEKVAKVTADEARLLRDVRNFYKLSKENFVSNADEDDFQ
DYAPRGGKRENRPRPGK*

Hydra oligactis

Gene 1

>TSA: Hydra oligactis contig23771 transcribed RNA sequence

MMFLTVTTFLLLTSLVVAMPSRDATDSNENDILNILDEYIVKVAEMTANEAKILNDVRNYYNDRSSKSLGEFP
QSFLPRGGKRDTRPRAGK*

Gene2

>TSA: Hydra oligactis contig27729 transcribed RNA sequence

MTHTAIFGFFILFTIVLALPQRDAYDLLDRFDEYIEKVAKVTLDEARLLRDVRNFYKLSKEENSVSNTDEDDF
QDYAPRGGKRENRPRPGK*

Hydra vulgaris

Gene 1

>Hydra vulgaris strain 105 HYDRAscaffold_37868_Cont37791, whole genome shotgun sequence

MLSLTVATLLLITSIVMAMPNRDATDSNESDILNILDEYIVKVAEMTANEAKILNDVRNYYNDRSSKSLGEFP
QSFLPRGGKRDARPRAGK*

Gene 2

>Hydra vulgaris strain 105 HYDRAscaffold_38933_Cont15348, whole genome shotgun sequence

MMRTAVFGCFILFTIVLALPYRDAFNLFDRFDEYIEKVAKVTADEARLLRDVRNFYKLSKENFVSNADEDDFQ
DYAPRGGKRENRPRPGK*

Millepora alcicornis

>GFAS01058816.1 TSA: Millepora alcicornis TRINITY_DN52382_c0_g1_i1 transcribed RNA sequenceMMAMLVVLVLMSFINAKLQASNVRSNDANTALQNSKRNVLSLRPRPGRSLELKSLSSKDRARWML
AELYDQIAQDLAKKVQSKRPLEKRRANFFDLLADKKESLLASDDIPRMGKRETKYNSYNWGSKNQRRSNSYLD
SLINNILDRSLSEKILQKRYLDDLMNAVDEDDEYFAKTLVK*

Millepora squarrosa

>GFGU01105351.1 TSA: Millepora squarrosa TRINITY_DN71008_c0_g1_i1 transcribedRNAsequence

MMAVLVVLVLMSFINAKLQASNVRSNDADTALQNVKRNVLSLRPRPGRSLELKSLSSKDRTRWMLAEIYDQIV QDLAKKVLPKRPIEKKRANFFDLLADKKESLLASDDIPRLGKRETKYNSYNWGSKNQRRSNSYLDSLINNILD RSLSEKILQKRYLDDLMNAVDEDDEYFAKTLVK*

Physalia physalis

Gene 1
>GHBB01025917.1 TSA: PHYSALIA PHYSALIS ISOLATE YOMITAN PHY_COMP26153_C0_SEQ1, TRANSCRIBED RNA SEQUENCE
MMAIHSIHALCIALQFLCYIGKGDSKSYNTGDLDFKSRDRNRMSDVRDSYYPINDNSYASKSFGRADSSKMDM LQSYDSNQDYYSTDVESEYKNANLLGRPRGGREILRDGDHSQYMLARPRGGKRRSIKDSERRRDMEMIDQDSR *

Gene 2
>gb|GHBB01019005.1|_translation_frame_+3
MEVTIVINMAHLLCVALLLSSCLCTRKLKSDDFTSEQLDDSANVNQKEDILLMDGKNYQNNALNHLSKSLDDG FRFSFYSPKPRDLKRMIEEIKHVEDDNEEEKYRLTSFFENLKRRIHSSITSEEGNHERNTFKGVTLMADPRGG REIHLRPRGGKRDCIETGLDEYLRDEEECFEG*

Gene 3
>TRINITY_DN61513_C1_G1_I3
MFVLLLLTSCLLPFSSMMPMNSKKVDAHRLPPRLGRDVSEVKALNEKEKSDELLPRLGQLPPRLGRDVPQVSA LNVKVKNDESLPQLEHLPPRLGRDVSQTSVETTKEKRNELPPRLGRELNPNSPKHLPPRLGRDVSQASVETTK EKRDELPPRLGRELPPNPLQHLPPRLGRDVSQASVETTKEKRDEIPPWQFWKFWKKGRGDEKE*

Gene 4
>GHBB01029094.1 TSA: Physalia physalis isolate Yomitan phy_comp27574_c0_seq3, transcribed RNA sequence
MFALLLFTTCLLSFSSMMPMDSKEVALQRLPPRLGRDVSQVSVLNTKDKSDELLHQLDQ

Podocoryna carnea

>GCHV01017056.1 TSA: PODOCORYNA CARNEA PODOCORYNA_41066.0_TRANSCRIPT_1/0_CONFIDENCE_0_LENGTH_1212 TRANSCRIBED RNA SEQUENCE
MRSPIILLFLFGVTLLVRGENTSESQYEGILASLLKYLPDSFFEEASEYLEDRQLNEDATQRHYHPRKFASFK PRGGKRDEHHHHIYRPRAGRSFETLGSRPRAGKREIRPRAGKRSLYRPRAGKRSIDRPRAGKRDLLRKMDHLR PRAGKRNSLEHRLQSEKRSARNHKKSTLKKDSKNTRSRRGLDVIDYEEGSDADFNLDYEDNKSLREDEYFDSE EFDSR*

Porpita porpita

>GHBA01045569.1 TSA: Porpita porpita isolate Yomitan por_comp44161_c0_seq1, transcribed RNA sequence
MMESMMKLALLFVFVSALYAKSINDKAIRNHMTNELNDQERYSLISRPRSGRSVDKRNVYDRLLHYLQQLKEL
KDNKDAAILDERSFGVSESFNDMVTEKLIHALYDTLSKKSTFKRFATSRQLMGLIPPRLGKKNVDDM*

Turritopsis sp.

>IAAF01041061.1 TSA: TURRITOPSIS SP. SK-2016 MRNA, CONTIG: C52423_G1_I2, TRANSCRIBED RNA SEQUENCE
GESQLLRGGKRQSERPRGGKRQLERPRGGKRSKNNDDSRQEERPRGGKRNLDDDYSRESQLLRGGKRHVNFGQ
SFRSERPRGGKRSTNNVKFRKSSFRPRDRKRNMDDDNSHKIQLLRGDKRYANFGQSFQAKRPLGGKTSRETER
PQGGKREMGDDNGRGESQLLRGGKRQSERPRGGKRQLERPRGGKRSKNNDDSRQEERPRGGKRNLDDDYSRES
QLLRGGKRHVKFGHYFQSERPRGGKRSSNNDSSREIERPRGGKREQDTNNDNYREWF*

Velella velella

>GHAZ01080105.1 TSA: Velella velella isolate Yomitan vel_comp94891_c0_seq1, transcribed RNA sequence
METKMILVLLLIFASALYGKHIKDKVIRNDMSSDIGDQTHYMAMGRPRSGRSLNKRSVYDHLLNYFHHLKEMK
DSKEMLNEKSELFSDMTTDELIHALYDTLSKKVSYKRFSSSQQLIPPRLGKKSVDDLD*
