# Supplementary material for: An evolutionary genomics view on neuropeptide genes in Hydrozoa and Endocnidozoa (Myxozoa)
Source: BMC Genomics. 2021 Nov 30;22:862. doi: 10.1186/s12864-021-08091-2 (PMC8638164; doi:10.1186/s12864-021-08091-2)
Supplement: Supplementary file 2 — Additional file 2. Partial or complete amino acid sequences of the GRFamide preprohormones in species belonging to the Hydrozoa (Part One) or the Endocnidozoa (Part Two). [file 12864_2021_8091_MOESM2_ESM.pdf]

Additional file 2. Partial or complete amino acid sequences of the GRFamide preprohormones in species belonging to the **Hydrozoa** (**Part One**) or the **Endocnidozoa** (**Part Two**). For some species more than one preprohormone fragment was identified, which sometimes indicated the presence of more than one gene or the presence of splicing variants. Signal sequences are underlined. An asterisk indicates a stop codon. Neuropeptide sequences are highlighted in yellow; C-terminal processing sites are highlighted in green. The C-terminal Gly residues that are converted into C-terminal amide groups are highlighted in red.

Part One: Hydrozoa

Clytia hemispherica

>TCONS_00033583-protein
MELKYFLASSIFVIIAIQLASCSSKAEEYKQMKKEVDGLLKEIVSQENAKQHTSEKKSSQWLNGRFGKRQLVS
GRFGRELKQWLNGRFGREATEQWLNGRFGKREADQWLNGRFGREVEQWLNGRFGRDAAEQWLNGRFGKRSANQ
WLNGRFGKRSADQWLNGRFGKRSADQWLNGRFGREASADQWLNGRFGREAADQWLNGRFGREAKEQWLNGRFG
REVEQWLNGRFGREAGQWLNGRFGREMGQWLNGRFGREADQWLNGRFGREADQWLNGRFGRDAAPLAARYGDE
PAHVESQTIASPEEAKPKVVAAVKVVKKPVAVSE*

This is the GRFamide preprohormone dubbed Che-pp5 and published in [35].It is nearly identical to our identified sequence, but four amino acid residues have been exchanged (highlighted in red color font).
MELKYFLASFIFVIIAIQLASCSSKAEEYKQMKKEVDGLLKEIVSQENAKQHTSEKKSSQWLNGRFGKRQLVS
GRFGRELKQWLNGRFGREATEQWLNGRFGKREDDQWLNGRFGREVEQWLNGRFGRDAAEQWLNGRFGKRSANQ
WLNGRFGKRSADQWLNGRFGKRSADQWLNGRFGREASADQWLNGRFGREAADQWLNGRFGREAKEQWLNGRFG
REVEQWLNGRFGREAGQWLNGRFGREMGQWLNGRFGREADQWLNGRFGREADQWLNGRFGRDAAPLAARYGDE
PAHVESQTIAAPEEAKPKIVAAVKVVKKPVAVSE

Craspedacusta sowerbii

Gene 1
>QQSS01031682.1 Craspedacusta sowerbii isolate 2016 scaffold128562_cov76, whole genome shotgun sequence
MIGKIPIHAVFALVLLCLAYCAKERKAIEQDDTKRAADQWLRGRFGKREESGVAKRQYYEEEEEEEEEEEEDD
DDDYDDKRAVDQWLRGRFGRESGEQWLRGRFGRELSDSARSNHGNSNSENKRAIVTADQWLRGK

Gene 2
>QQSS01345190.1 Craspedacusta sowerbii isolate 2016 scaffold1839387_cov37, whole genome shotgun sequence
MIGKIPISAVFALALLCLAYCAKETKATEQNDAKRAADQWLRGRFGKREENGVAKRQYYEEEEEEEEEEEDDD
DDYDDKRGADQWLRGRFGRESGEQWLRGRFGRELSDSASSHHGSINSDNKRAIAAADQWLRGKRDLSWITSWS
HM

Gene 3
>QQSS01406432.1 Craspedacusta sowerbii isolate 2016 scaffold2247036_cov82, whole genome shotgun sequence
MNRTVLVYAFVALTVLCLAYCAEETKAIDQELEDAKRAIDQWLRGRFGKREDGVAKRQGYEEEEEEEEDDDDD
DKKRSVDQWLRGRFGRESEEQWLRGRFGRELNDWIKSHEGSSSEDKRASLTTDQWLRGKYAHFLMRLFL

Gene 4

>QQSS01304015.1 Craspedacusta sowerbii isolate 2016 scaffold1568862_cov37, whole genome shotgun sequence

MLLMTIATTLLALQIHAYSLDRKENVNWASRRLDAKDSFRPLIPRPRFFNKRLVYLTENDLKNIYHILLEEPG
ADTIAIDYDDHQEKNLNKGDSNGQYPVDFKPREHSRTVRDAQERQEADAADKVGVYRLTKGNSDIKDQSIHTS
LDKNPARDVIDSRRLAREQQRWGRFGGDVSNGQQPRWGRFGREDITAEGLSKDITKYHRAFVRRDTGPSENKS
SMQWSRFGRELAEALQPRWGRFGDGATKRSSPHWGRFGREAKKEETVSNPRWGRFGRDTDEVLAEPR

Gene 5

>QQSS01050098.1 Craspedacusta sowerbii isolate 2016 scaffold208766_cov37, whole genome shotgun sequence

MLVECITTLFLALQIHGHVLGRQERSPNELLGGPEARDGFRPLIPRTRFFNKRLIYLTDNGLLDMYRTLFGGL
PKDRTEGSYDDNLTSNLSDAGMNVPTPANTMHVEHAKQRENVEKAIISLPTVADSANDVDADGETLDSNLKGR
YRKTAQEETPMPNFRDQGRFKRRQQRWFRLGGDIHSGQQPRWGRFGRGDSGRSVVEDSSLYHDRELKTDTGSV
EYKSGLQWGRFGRELKEAIQPLWGRFGDVAHKLSSPRWGRFGRQTRNDEDSGDPLWGRFGRESLEAEPRWGRF
GRETMKDVPSDPLWGRFGRESMETEPRWGRFGRQIQKDVPDGLLWGRFGRESIEAEPRWGRFGRESIEAEPRW
GRFGRESIEAEPRWGRFGREIKNDEVLGYSIKGRQGSFVNERSW

Gene 6

>QQSS01278138.1 Craspedacusta sowerbii isolate 2016 scaffold1411248_cov50, whole genome shotgun sequence

MLVKCIATFFLALQVHAHVLDCQEKNLNELIRVAEARDFFRPLIPRTRFFNKRLVYLTDNELLDMYRTLLGGL
ARDRTEVAYDDHLASNFSDADTNAQTPTDTMHADHAKHKKNAEKEFIPLLTVADSTDDVDVNGETVNSNREYR
YSKTATEETLMPDPYPVRDPGRFKKRQQRWFRLGGDSYSSQPPRWGRFGRRDADKSLIEDSRLHYGGELKTET
GSADYKSGSQWGRFGRELAEAIQPLWGRFGDAAYKLSSPRWGRFGRQTRKEEVSGDPLWGRFGRESLGGEPRW
GRFGRQAKKDVPGDPLWGRFGRESVEGEERWRRFGRQAKKEKESGDPLWGRFGRESLEAEPRWGRFGRQAIKG
VPDDPLWGRFGRESLETEPRWGRFGRQTMKDVPDDLLWGRFGREFETEPRWGRFGRETKKDKVPGDNL

Dynamena pumila

>GHMC01016042.1 TSA: DYNAMENA PUMILA ISOLATE DYNAMENA PMILA COLONY DYNAMENA16041, TRANSCRIBED RNA SEQUENCE

MELRDLIIVVGCMLLSTCTLIYGKESKENLDLLKKEVDELLKDVEKGSHEKRQKNIVGRFGKEIPSEEENIAE
ADTEKRELEDQWLKGRFGRELSDQWLKGRFGREVEEQWLKGRFGREATEQWLKGRFGREVADQWLKGRFGRES
SNQWLKGRFGRELSDQWLKGRFGREVEEQWLKGRFGREVSEQWLKGRFGREASDQWLKGRFGREATEQW

Hydractinia symbiolongicarpus

>GAWH01026400.1 TSA: Hydractinia symbiolongicarpus Hs_transcript_26402 transcribed RNA sequence

MLIMASKAMVFAVIFITSYVTAKDTGKLQKKESHNEETKLASLLDDIISAEENSHATEPKELDQWLKGRFGRE
ADQWLKGRFGREAEQWLKGRFGRESDQWLKGRFGRESEQWLKGRFGREAEQWLKGRFGREAEQWLKGRFGREA
EQWLKGRFGRENEQWLKGRFGRENEQWLKGRFGRENDQWLKGRFGRENEQWLKGRFGRENEQWLKGRFGRENE
QWLKGRFGREIEQWLKGRFGRESDAEILKGFGNDVASSQWLKGRFGRAESNGQHRNMIGKRETLPLRRGRYGK
EVEEYNKKEVDQWLKGRFGREAEQWLKGRFGREMEQWLKGRFGREAEQWLKGRFGRSTQTESETKKSVSKDNT
KVETVKKSVKKM*

Hydra magnipapillata

Gene 1

Gene 1 codes for preprohormone A that we cloned in 1998,NCBI# Y11678, https://www.ncbi.nlm.nih.gov/pmc/articles/PMC1219495/ It is identical to the sequence below.

>GAOL01017548.1selectiontranslationframe+3
MLSNKKVELLFALVFVVVAVVRSEDKKLSLEDNKDVKRIVNDYLETKNGEQLMSGRFGKRETDEADSDDEDSS
EYENEYDDELENQGLANARYERQLMRGRFGREKNAASNEDQWLGGRFGREAATQWFNGRFGRDIEGRFLPRFA
KESNKPHLRGRFGRAVKL*

Gene 2

Gene 2 codes for preprohormone B that we cloned in 1998, NCBI# Y11679, https://www.ncbi.nlm.nih.gov/pmc/articles/PMC1219495/

>GAOL01026530.1selectiontranslationframe+1
MLSYKKFELLFALVLIVVEVVKSDDKNFSLEVNKDVKRFIKDILDAKSEEQLMSGRFGKSLPDEEDIDNEVEN
EYDNEYDDETESQGIINGRYGRQLLRGRFGRQNDNNAASKENQWLGGRFGKEVATQWFNGRFGREIGGRFLPR
FAREFNKPHYRGRFGRIAKL*

Gene 3

>GAOL01005393.1selectiontranslationframe+3
MLNHKIETFLVWGLIIVAVAKSEEKNFSAEDRKDVKRIVNDYLNIKNGEQLMTGRFGKRVTDEDIDNEIESEY
ENEYEDELENLANGREDAAQWFNGRFGREIGGRILPRFATESNKPHLRGRFGRAAKM*

Gene 4

>GAOL01013683.1selectiontranslationframe-3
MLNQRKVELFFALALIVVALVKSDNNNHLSEDNKNNNHLSEDNKNNNHLSEDNKNIKRIIEDYLNAKNAEQLT
RGKFMKKITNKEDDFKNEAEYEYENKYGDELKNHVLVNKREDAAQWFNGRFGREMGERFLPRFGKELNKPHLR
GRFGRSLKL*

Transcript 5

Transcript 5 codes for preprohormone C that we cloned in 1998, NCBI# Y11680, https://www.ncbi.nlm.nih.gov/pmc/articles/PMC1219495/

>Preprohormone C
MATNMALLTFILFATSIFMLAKADSQNEDNQKYAGIARSLKVLLQNYYQKQEEKSDIQNIIEKFSEYQNTDHK
RNDKTNPMFEKKDSDTENRFNREAIEQWFSGRFGLPNQKRNNEVNPMIEKKDSDIENRFNRESLEQWLSGRFG
LTNQKRHNEANPMIEKKDSDTENRFNKETIEQWLSGRFGLTNHKRNNEVNPMIEKKDSDTENRFNRESLEQWL
SGRFGLTNHKRNNEVNPMIEKKDSDTENRFNRESLEQWLSGRFGLTNHKRNDEANPMIEKKDSDTENRFNRES
LEQWLSGRFGLTNHKRNNEVNPMIEKKDSDTENRFNRESLEQWLSGRFGLTNHKRNDEVNPMIEKKDSENENR
FNRESIEQWLGGRFGRTVYEFLLSETSEKRKK*

Hydra oligactis

Gene 1

>GBFD01012965.1 TSA: Hydra oligactis contig22242 transcribed RNA sequence

MLSKNKELLFAFVLIVVTVVKSEDKNLSSEDNKDVKRVVKDYLDTKNGEQIMSGRFGKRETDEEDIDNEDKSE
AENELDDELGNQGDNGRYERQLMRGRFGRERNAASNEDQWLGGRFGREFANQRFNGRFGRDIEGRFLPRFAKE
FNKPHLRGRFGRAAKL*

Gene 2
>GBFD01020678.1 TSA: Hydra oligactis contig24196 transcribed RNA sequence
MFSFKKVETFFALVLIVVAFVKSEDKNLSSEDRKDVKRIVKDYLYTKNGEQLMTGRFGKRVTDEDIDNEIENE
YENEYEDALVNGREDATQWFNGRFGRELGGRILPRFATESNKPHLRGRFGRAAKL*

Gene 3
>PJUT01227504.1selectionselectiontranslationframe+1
MLKRVELLFAFVLIVVSVVKSDDKNLSSEDNVNRVATDYLDTKNEEQLMSGRFGKSVTDEDDTDNEVEIEYDN
DYNGNFYKNFQNLFLCLLIQFQFLHFDFCKRLDRLKIAYINFTNIRYRYVVDTLIVPIYYYLDEQVLHGPTVG
RYGRQILRGRFGKQYNAANNEDQWLGGRFGREDQWLGGRFGRENQWLGGRFGKEVANQWFNGRFGREVGGRFL
PRFEKDSNKPYYRARFGRVAKL*

Gene 4
>GBFD01017912.1TSA: Hydra oligactis contig29949 transcribed RNA
sequenceMLNQRVKLFLFLALIVVAVVKSDDKNFSSEDSKNLKRIIKDYVDTKSEEQLMRGRFGKRVTDEDI
DNEVENEYENEYDDLNIDK*

Gene 5
>PJUT01399962.1selectionselectiontranslationframe-1
MLSQRVKLFLFLALIVVAVVKSDDKNFSSEDSKNLKRIIKDYVDTKSEEQLMRGRFGKRVTDEDIDNEVENEY
ENEYDGNKIFLLIAFKIPFAL*

Gene 6
>PJUT01265764.1selectionselectiontranslationframe-1
MQVIIILKSFVKEKTMQRENTGNPMFEKKDALNPMFEKKDAIEQWMSGRFGRVVYDSLLSEVSKGFFKLIFYN
CQHHSLCSYFL*

Hydra vulgaris

Gene 1
>GGKH01002512.1 TSA: Hydra vulgaris c12338_g1_i01 transcribed RNA
sequence
MLSNKKVELLFALVLVVVAVVRSEDKNLLLEDNKDVKRIVNDYLETKNGEQLMSGRFGKRETDEGDSDDEDSS
EYENEYDDELENQGLANVRYERQLMRGRFGREKNAVSNEDQWLGGRFGREAATQWFNGRFGRDIEGRFLPRFA
KESNKPHLRGRFGRAAKL*

For comparison: Gene 1 from H. magnipapillata. Seven differences are indicated by red font color.
>GAOL01017548.1selectiontranslationframe+3
MLSNKKVELLFALVFVVVAVVRSEDKKLSLEDNKDVKRIVNDYLETKNGEQLMSGRFGKRETDEADSDDEDSS
EYENEYDDELENQGLANARYERQLMRGRFGREKNAASNEDQWLGGRFGREAATQWFNGRFGRDIEGRFLPRFA
KESNKPHLRGRFGRAVKL*

Gene 2

>gi|1069011567|emb|HAAC01002650.1|_translation_frame_+2
MLSYKKFELLFALVLIVVEVVKSDDKNFSLEVNKDVKRFIKDILDAKSEEQLMSGRFGKSLPDEEDIDNEVEN
EYDNEYDDETESQGIINGRYGRQLLRGRFGRQNDNNAASKENQWLGGRFGKEVATQWFNGRFGREIGGRFLPR
FAREFNKPHYRGRFGRIAK*

For comparison: Gene 2 from H. magnipapillata. The two sequences are identical with the exception of the last amino acid residue (= one difference).

>GAOL01026530.1selectiontranslationframe+1
MLSYKKFELLFALVLIVVEVVKSDDKNFSLEVNKDVKRFIKDILDAKSEEQLMSGRFGKSLPDEEDIDNEVEN
EYDNEYDDETESQGIINGRYGRQLLRGRFGRQNDNNAASKENQWLGGRFGKEVATQWFNGRFGREIGGRFLPR
FAREFNKPHYRGRFGRIAKL*

Transcript 3

>gb|GEVZ01016017.1|_translation_frame_+3
MATNMALLTFILFATSIFMLAKADSQNEDNQKYAGIARSLKVLLQNYYQKQEEKSDIQNIIEKFSEYQNTDHK
RNDKTNPMIEKKDSDTENRFNREAIEQWFSGRFGLPNQKRNNEVNPMIEKKDSDIENRFNRESLEQWLSGRFG
LTNQKRHNEANPMIEKKDSDTENRFNKETIEQWLSGRFGLTNHKRNNEVNPMIEKKDSDTENRFNRESLEQWL
SGRFGLTNHKRNNEVNPMIEKKDSDTENRFNRESLEQWLSGRFGLTNHKRNDEVNPMIEKKDSENENRFNREC
IEQWLGGRFGRTVYEFLLSETSEKRKK*

For comparison: Transcript 5 from H. magnipapillata. The two sequences are nearly identical in the N-terminal part, but the C-terminal part of the H. magnipapillata sequence has an insertion highlighted in red font, due to alternative splicing (see Fig.3).

>Preprohormone C
MATNMALLTFILFATSIFMLAKADSQNEDNQKYAGIARSLKVLLQNYYQKQEEKSDIQNIIEKFSEYQNTDHK
RNDKTNPMFEKKDSDTENRFNREAIEQWFSGRFGLPNQKRNNEVNPMIEKKDSDIENRFNRESLEQWLSGRFG
LTNQKRHNEANPMIEKKDSDTENRFNKETIEQWLSGRFGLTNHKRNNEVNPMIEKKDSDTENRFNRESLEQWL
SGRFGLTNHKRNNEVNPMIEKKDSDTENRFNRESLEQWLSGRFGLTNHKRNDEANPMIEKKDSDTENRFNRES
LEQWLSGRFGLTNHKRNNEVNPMIEKKDSDTENRFNRESLEQWLSGRFGLTNHKRNDEVNPMIEKKDSENENR
FNRESIEQWLGGRFGRTVYEFLLSETSEKRKK*

Gene 4

>gb|GHHG01002052.1|_translation_frame_+2
MLSNKKVKLLFALVLIVVEVVKSDDKNFSLEVNKDVKRFIKDILDAKSEEQLMSGRFGKSLPDEEDIDNEVEN
EYDNEYDDETESQGIINGRYGRQLLRGRFGRQNDNKAASKESQWLGGRFGKEVATQWFNGRFGREIGGRFLPR
FGREFNKPHYRGRFGRVAKL*

For comparison: Gene 2 from H. magnipapillata. Seven differences are indicated by red font color.

>GAOL01026530.1selectiontranslationframe+1
MLSYKKFELLFALVLIVVEVVKSDDKNFSLEVNKDVKRFIKDILDAKSEEQLMSGRFGKSLPDEEDIDNEVEN
EYDNEYDDETESQGIINGRYGRQLLRGRFGRQNDNNAASKENQWLGGRFGKEVATQWFNGRFGREIGGRFLPR
FAREFNKPHYRGRFGRIAKL*

Gene 5

>GGKH01059288.1 TSA: Hydra vulgaris c8038_g1_i01 transcribed RNA sequence
MLNHKIETLLVWGLIIVAVVKSEDKNLSAEDRKDVKRIVKDYLNIKNGEQLMSGRFGKRVTDEDIDNEIESEY
ENEYEDELENFANGREDAAQWFNGRFGREIGGRILPRFATESNKPHLRGRFGRAA*

For comparison: Gene 3 from H. magnipapillata. Nine differences are indicated by red font color.

>GAOL01005393.1selectiontranslationframe+3
MLNHKIETFLVWGLIIVAVAKSEEKNFSAEDRKDVKRIVNDYLNIKNGEQLMTGRFGKRVTDEDIDNEIESEY
ENEYEDELENLANGREDAAQWFNGRFGREIGGRILPRFATESNKPHLRGRFGRAAKM

Gene 6

>XP_002167764.1 PREDICTED: pol-RFamide neuropeptides-like [Hydra vulgaris]
MLNHKIETFLVWGLIIVAVAKSEEKNFSAEDRKDVKRIVNDYLNIKNGEQLMTGRFGKRVTDEDIDNEIESEY
ENEYEDELENLANGREDAAQWFNGRFGREIGGRILPRFATESNKPHLRGRFGRAAKM*

For comparison: Gene 3 from H. magnipapillata. The two sequences are identical.

>GAOL01005393.1selectiontranslationframe+3
MLNHKIETFLVWGLIIVAVAKSEEKNFSAEDRKDVKRIVNDYLNIKNGEQLMTGRFGKRVTDEDIDNEIESEY
ENEYEDELENLANGREDAAQWFNGRFGREIGGRILPRFATESNKPHLRGRFGRAAKM*

Gene 7

>XP_002170240.2 PREDICTED: pol-RFamide neuropeptides-like [Hydra vulgaris]
MVKMLNQRKVELFFALALIVVALVKSDNNNHLSEDNKNNNHLSEDNKNIKRIIEDYLNAKNAEQLTRGKFMKK
ITNKEDDFKNEAEYEYENKYGDELKNHVLVNKREDAAQWFNGRFGREMGERFLPRFGKELNKPHLRGRFGRSL
KL

For comparison: Gene 4 from H. magnipapillata. The H. vulgaris sequence has a signal sequence that is three residues longer as indicated by red font color. It has also a deletion of 10 residues (indicated in red).

>GAOL01013683.1selectiontranslationframe-3
MLNQRKVELFFALALIVVALVKSDNNNHLSEDNKNNNHLSEDNKNNNHLSEDNKNIKRIIEDYLNAKNAEQLT
RGKFMKKITNKEDDFKNEAEYEYENKYGDELKNHVLVNKREDAAQWFNGRFGREMGERFLPRFGKELNKPHLR
GRFGRSLKL

Transcript 8

>GGKF01043920.1selectiontranslationframe+2
MVTNMALLAFILFATSIFMLAKADSQNEDNQKYAGIARSLKVLLQNYYQKQEEKSDIQNIIEKFSEYQNTGKI
IQRKNNVNPMFEKKDAIEQWLGGRFGRAVYDLLWSEVSKGLQSTFINFLNKLHHSLIF*

For comparison: Transcript 5 from H. magnipapillata. Also here, we propose alternative splicing. See Fig.3.

MATNMALLTFILFATSIFMLAKADSQNEDNQKYAGIARSLKVLLQNYYQKQEEKSDIQNIIEKFSEYQNTDHK
RNDKTNPMFEKKDSDTENRFNREAIEQWFSGRFGLPNQKRNNEVNPMIEKKDSDIENRFNRESLEQWLSGRFG
LTNQKRHNEANPMIEKKDSDTENRFNKETIEQWLSGRFGLTNHKRNNEVNPMIEKKDSDTENRFNRESLEQWL

SGRFGLTNHKRNNEVNPMIEKKDSDTENRFNRESLEQWLSGRFGLTNHKRNDEANPMIEKKDSDTENRFNRES
LEQWLSGRFGLTNHKRNNEVNPMIEKKDSDTENRFNRESLEQWLSGRFGLTNHKRNDEVNPMIEKKDSENENR
FNRESIEQWLGGRFGRTVYEFLLSETSEKRKK*

Millepora alcicornis

>GB|GFAS01199724.1| TSA: MILLEPORA ALCICORNIS TRINITY_DN121470_C0_G1_I1
TRANSCRIBED RNA SEQUENCE
MMMNGLSLILFMSVITPYFITCEKLVAKTKREMDELEEDVNELLRDVLSDEKEQLHKDSKQWHAGRFGRESDQ
WLKGRFGREANQWLKGRFGREAEQWLKGRFGREAEQWLKGRFGRELADQWLKGRFGRETEQWLKGRFGRENDQ
WLKGRFGRERSNEDQWLKGRFGREVERWLKGRFGQEERDAQWLKGRFGRELADQWLKGRFGRDSSAQLVRGRY
GGDQPSEQSDSSDKQLVRGRYGGDQWLKGRFGREVDDKVNSKENNMIDAVKNDDKKSLKVENNEQKSM*

Millepora complanata

>GFGT01132665.1 TSA: MILLEPORA COMPLANATA TRINITY_DN85097_C0_G1_I1
TRANSCRIBED RNA SEQUENCE
MMMNGLSLILFMSVITPYFITCEKLVAKTKREMDELEEDVNELLRDVLSDEKEQLHKDSKQWHAGRFGRESDQ
WLKGRFGREANQWLKGRFGREAEQWLKGRFGREAEQWLKGRFGRELADQWLKGRFGRETEQWLKGRFGRENDQ
WLKGRFGRERSNEDQWLKGRFGREVERWLKGRFGQEERDAQWLKGRFGRELADQWLKGRFGRDSSAQLVRGRY
GGDQPSEQSDSSDKQLVRGRYGGDQWLKGRFGREVDDKVNSKENNMIDAVKNDDKKSLKVENNEQKSM*

Millepora sp. (RR-2016)

>GFGV01123832.1 TSA: MILLEPORA SP. RR-2016 TRINITY_DN82396_C0_G1_I1
TRANSCRIBED RNA SEQUENCE
MMMNGLSLILFMSVITPYFITCEKLVAKTKREMDELEEDVNELLRDVLSDEKEQLHKDSKQWHAGRFGRESDQ
WLKGRFGREANQWLKGRFGREAEQWLKGRFGREAEQWLKGRFGRELADQWLKGRFGRETEQWLKGRFGRENDQ
WLKGRFGRERSNEDQWLKGRFGREVERWLKGRFGQEERDAQWLKGRFGRELADQWLKGRFGRDSSAQLVRGRY
GGDQPSEQSDSSDKQLVRGRYGGDQWLKGRFGREVDDKVNSKENNMIDAVKNDDKKSLKVENNEQKSM*

Millepora squarrosa

>GFGU01110161.1 TSA: MILLEPORA SQUARROSA TRINITY_DN73250_C0_G1_I1
TRANSCRIBED RNA SEQUENCE
MKTSTMNGLSLILFMSVITPYFITCEKLVAKTKREMDELEEDVSELLRDVLSDEKEQLNKDSKQWHAGRFGRE
SDQWLKGRFGREAGQWLKGRFGREAEQWLKGRFGRELVDQWLKGRFGREAEQWLKGRFGRENDQWLKGRFGRE
SSNEDQWLKGRFGREIEQWLKGRFGREEGDAQWLKERFGRELADQWLKGRFGRDFSSQLVRGRYGGDQPSEQA
DSSDKQLVRGRYGGDQWLKGRFGREVDDKVNSKVNSMIDTVKKDDKKSLKVDNNEQKSM*

Physalia physalis

>GHBB01022770.1 TSA: PHYSALIA PHYSALIS ISOLATE YOMITAN
PHY_COMP24576_C0_SEQ1, TRANSCRIBED RNA SEQUENCE
MAFNTKSSCFVLILVVSLYGIATGGLLNNKKEIEEAGKELARILDDVMYEDRKKEKRETDENSLNGQSDRENE
QWLKGRFGREAVEQWLKGRFGREEDQWLKGRFGRESNKFEKTFSERESDEPTKDYNGNEDSQWLKGRFGREVI
EQWLKGRFGREVEDEETQWLKGRFGRALSEQWLKGRFGRSFSNDIPNKRHNDVTYKEDKYARKFHERFQREVE
QWLKGRFGREMDQWLKGRFGREIEQWLKGRFGRESKESKEVKSTKDNKIEKKFRKSTKKM*

Podocoryna carnea

>GCHV01024366.1 TSA: PODOCORYNA CARNEA PODOCORYNA_46417.0_TRANSCRIPT_1/0_CONFIDENCE_7_LENGTH_1563 TRANSCRIBED RNA SEQUENCE
MKLTLDIFILIVAIIYLTSATEIKKSSKKEISKEEEREITKLLDDLAKVGEEEKSHTVETKEDGQWLKGRFGR
ESEQWLKGRFGREAEQWLKGRFGREAGQWLKGRFGREIEQWLKGRFGREADQWLKGRFGRESEQWLKGRFGRD
EQWLKGRFGRDENSEQWLKGRFGRESSSLPLRRGRYGKEVEESNSEAEQWLKGRFGREAEQWLKGRFGRSTNS
NKESKNNTSGNKSVKTEKKSVKNM*

Porpita porpita

>GHBA01057914.1 TSA: PORPITA PORPITA ISOLATE YOMITAN POR_COMP48537_C0_SEQ1, TRANSCRIBED RNA SEQUENCE
MTIFELILLLSIAYPLFINCEKLITVEKTDKQSDDETIAKVLNYVSDISDILDSTSSNEPADDQWLRGRFGRE
ASDQWLRGRFGREANDQLEQSKNEADEQWLRGRFGREANDQWLRGRFGREADDQWLRGRFGRSADDQWLRGRF
GDAAEDQWLRGRFGREANDQWLRGRFGDEVDDQWLRGRFGREADEQWLRGRFGREASDQWLRGRFGREASDQW
LRGRFGRDADSQWIKNRFGKDVDQNTKQSGGRYGRDEKLPQQIRGRYAGDATDSASNKPQGRFGRDEKAKTQH
PHRARYTGGVIVSDDKKPLDRGRYGRNEEEKSNEQRFVRNINEQTDSNKNSSKSK*

Turritopsis

>IAAF01021964.1 TSA: TURRITOPSIS SP. SK-2016 MRNA, CONTIG: C41501_G1_I1, TRANSCRIBED RNA SEQUENCE
MKYNSILLCSFFTLACLHVSFTEEIQTAKDTSTTADDETNKLLDELLELTEEETPSDQWLKGRFGREVEQWLK
GRFGREADQWLKGRFGREVEQWLKGRFGREMDDKQWLKGRFGKDGPRRRGRFGDYSSDEANKETGQWLKGRFG
REVEQWLKGRFGREADQWLKGRFGREVEQWLKGRFGREMDDKQWLKGRFGKDGPRRRGRFGDYSSDEANKETG
QWLKGRFGREVEQWLKGRFGRDASAENKSQEKKSSM*

Velella velella

The N-terminal part of the preprohormone:

>GHAZ01091818.1 TSA: VELELLA VELELLA ISOLATE YOMITAN VEL_ _C0_SEQ1, TRANSCRIBED RNA SEQUENCE
MTTFELILLLSIAYPLFINCEKVITVEKSNDDSDDETIAKVLKYISNDILENTSSNKENKAADEQWLRGRFGR
EASDQWLRGRFGREASDQWLRGRFGREAEDQLENSKDQAKDQWLRGRF

The probable C-terminal part of the same preprohormone:

>GHAZ01060575.1 TSA: VELELLA VELELLA ISOLATE YOMITAN VEL_COMP79882_C0_SEQ1, TRANSCRIBED RNA SEQUENCE
QWLRGRFGREASDQWLRGRFGREADGQWIKSRFGKDAKQKELPEAQDSTVPSEQGRFGREAEGKPLHRGRYSG
DVTDNNGQKQTNPGTEGRFGKDTSKGRFVRNINEKTNSNKLISEHTKKSTTSK*

Part Two: Endocnidozoa

Buddenbrockia plumatellae

>BPE00000043 Buddenbrockia plumatellae SMART cDNA library (Clontech) Buddenbrockia plumatellae cDNA, mRNA sequence
XFYCNLIICENDALKIDALDSESDKELLKKEVSNEMVKSKLARSADQWARGRYGRDANQWARGRYGRDATIND
NQWARGRYGRNIDYDQWARGRYGRESNQWARGRYGRDLNEDQWARGRYGGDLNDDQWARGRYGGDLNDDQWAR
GRYGRGIKNSNDEKLVNNNSEMNKRFFYYDDIYDDYFDDFYDYYDDK

Polypodium hydriforme

Gene 1

This is the N-terminus of the protein:
>Polypodium hydriforme c20737_g1_i1 transcribed RNA sequence
MRLGLIVVLLCLSSPAFAEPSSETVEVSSKVSELTLEEKEAILRKALIIMVEEDLGSLKKQIAAKVLGKTSES
DSEDARISRDSLSEQWLRGRFGREAFEQWLRGR

This is probably the C-terminus of the same protein:
>GBGH01004839.1selectiontranslationframe+1
RESSEQWLRGRFGRESSEQWLRGRFGRSLDNANNQWIKGMADRKNSSDGLQETNAQVM*

Gene 2

This is the C-terminus of a different protein
>Polypodium hydriforme c6631_g1_i1 transcribed RNA sequence
YAQWIKGRFGRSLDEAGNQWIKGRFGRDKDSNSSEVELPQQNLATDLSSSKNGMADRKNSSDGLQETNAQVM*
