# Supplementary material for: An evolutionary genomics view on neuropeptide genes in Hydrozoa and Endocnidozoa (Myxozoa)
Source: BMC Genomics. 2021 Nov 30;22:862. doi: 10.1186/s12864-021-08091-2 (PMC8638164; doi:10.1186/s12864-021-08091-2)
Supplement: Supplementary file 3 — Additional file 3. Partial or complete amino acid sequences of the GLWamide preprohormones in species belonging to the Hydrozoa (Part One) or the Endocnidozoa (Part Two). [file 12864_2021_8091_MOESM3_ESM.pdf]

Additional file 3. Partial or complete amino acid sequences of the GLWamide preprohormones in species belonging to the **Hydrozoa** (**Part One**) or the **Endocnidozoa** (**Part Two**). For some species more than one preprohormone fragment was identified, which sometimes indicated the presence of more than one gene or the presence of splicing variants. Signal sequences are underlined. An asterisk indicates a stop codon. Neuropeptide sequences are highlighted in yellow; C-terminal processing sites are highlighted in green. The C-terminal Gly residues that are converted into C-terminal amide groups are highlighted in red.

Part One

Clytia hemispherica

Gene 1

The below sequence was recently published in [35] and dubbed Che-pp11.
>Che-pp11
MDQSLSSILLLLCCWVALTTCMSVQRKEAGDALSALDKENAKKSANSITEELARNLMEHLYDEIRKRSNSNEE
TISNFRRASSDTHRQQQAPKGLWGRELQPGNPPGLWGREASEAENTDSNDGPIPGMWGRREADDKNAHEKFQ*

Gene 2

The below sequence was recently published in [35] and dubbed Che-pp2.
>Che-pp2
MKIYFGCLFVILSVNQIGCYPSSNQNSERELVRRIYKTVHPNPHYQVNEIQRVKEALKRRVLENVHRVDSLKA
SLKRVLGQDAGNGFHMSSSKIFQKKRSKARLPHSYMFRKRQNSPGALGLWGREVEAPGDIGPPGIWGDVVPDE
TRKDKPGAVQGLWGKDERVIRALLKTLKR*

Gene 3

The below sequence was identified by us in the current paper.
>TCONS_00025986-protein
MQWFDTVFFSSIILLLLLNFVIAEQHNHEMTPPPGLWGREMPMSMMRVQRQKEKKLPMKFGRETSAINHALPM
KFGREATTRDRKLPMKFGREASTRDRKLPMKFGREATTRDRKLRMKFGREATTKDRKLQMKFGRELPMKFGRS
SKSEHQEVTTFDRQLPRIILGRELTTTTDNQLSRRKSTKNLLKRRESFVEQILSNEIRREFFNKDIVKRFLTL
YWNKARQDRYKNNKFQ

Craspedacusta sowerbii

Gene 1

>QQSS01014304.1 Craspedacusta sowerbii isolate 4NJM1BBP0Z
scaffold56580_cov35, whole genome shotgun sequence
MLRFSRMETRRAGMLLACAIAISYAASAENKRYLHDDRTVREFDVVPPRRSSSWTSNLPEADLRKLRTQDHSL
DDTHTLEEIIRELVSGESVKVWGNKALNKDLRDLFDSYVNTYKKSKSENYTPKGVWGKDDSSTDEREKRQDNI
PAGVWGREDTRNSIHQAGWEGHEGQSKFEGGLGDAETERPVGVWGDSETTRPVGVWGDSESIRPVGVWGDAET
KRPVGVWGDSESNRPVGVWGREDGEESTAWEQDLNSHKNLARTWGREAEYKATKKVNAEADVPQLGIDNLAET
GIGRPAGARGGRYGEGNNIRPAGMWGRDSNFEGAFRAWKRSAESRLGRPAGLWGREQPQRPPGLWGRESGNIV
ESAKPEGSEAGEKLKLPPGISPGKREE*

Gene 2
>QQSS01409292.1 Craspedacusta sowerbii isolate 4NJM1BBP0Z scaffold2303982_cov77, whole genome shotgun sequence
MERRKVNTLLLYTIIICCAVSAENNRLLREDARLVEFDALGLEPPSSSNNFHDGVKRNMVTQNCSQDGIHKLG
EGSEMVNGKSAKEWGTKTRNKDLSILFDSYAKDYKKNKLDNYTPNRVLAKDESFIIEREKRQNNIPAGVWGRE
DTQNSIEEGDLENRKSLGGIAGVREDSELSRPAGIWNREDDMKGPELPAGVWGRDMNSKNMAGVWNRAVEQGT
AFKANQEAAAISPGLDSLDHVYTEENVWEKQQKSRENRRPRDNLGHNSKSTTKDTAFGVWARSAGLKLERPSR
KSEGELRRSSGKQERSLDSQPQRPPGLWGRQLESIVKKAKPEASQAEEKMKLPPGILPGRREE*

Gene 3
>QQSS01046958.1 Craspedacusta sowerbii isolate 4NJM1BBP0Z scaffold194701_cov71, whole genome shotgun sequence
MERRRVWILIVCAIAISYAASAENKRYLHDDRTAREFDVVPPRRSTSWTNNLLDADLRKLRTQDYSLDDTHTL
EEIIRELVNGESIKVWGNKALNKDLSDLFDSYANTYKKSKSENYTPIGVWGRDESSTDEMEKRQDNIPAGVWG
REGNQNSIQQGGSDGHESQNKPAPVLGDTESKRPVGVWGDAESKRPVGVWGDAESKRPXXXXXXXXXXXXXXX
XXXMGSRGC*

Dynamena pumila

>GHMC01023148.1 TSA: DYNAMENA PUMILA ISOLATE DYNAMENA PMILA COLONY DYNAMENA23146, TRANSCRIBED RNA SEQUENCE
MAKLTLLMVSMAVAVVSCIAVDKSTKTSQDQISTLDDENNQKRMLVEEIVKQLVESLHGELKKRSLAPPKQRP
PGLWGREIGSSDVLGRQIEKPPGLWGREIEKPVGLWGREDQKPVGLWGREEQKPGGLWGREEQKGLWGREAQK
GLWGREVQKGLWGRGAQKPVGLWGREEQKPVGLWGREAQKPVGLWGRDAQKTVGLWIRHAQKPFGVWGREVQK
PVGLWGRDAQKPVGLWGREEQKPVGLWGRQIGKPPGLLGREQRLAAPALWKRSKESTKTEGKPPGMWGKDTID
ERSSDATLKTKVKNDDDSNRTKM*

Hydractinia symbiolongicarpus

Gene 1
>GAWH01054631.1 TSA: Hydractinia symbiolongicarpus Hs_transcript_54638 transcribed RNA sequence
MRNLILLVLLTVLLDKGIGKCNAKNEEDRDGNARNNRIDKNDEDSDSIEKYLREVTDELSKILAKRIYRDIQL
RENNKKAENRQSWIGDLENLDIDSTVQRPPGLWGREADFDNNRAHDSAQISDEKPPGIWAGDAKPPGLWGRDA
KPPGLWGRDAKPPGLWGRDAKPPGLWTGDAKPPGLWGRDAKPPSLWSKDNNVIKSQSEDAKPPGLWGRQVEDG
PTKIWGDGFLDAERHIRLLKNDERFNRLEKKVDMEEEVRIAQGPSVTKDTFGELADLLRKRIVKRLHKTDSLN
NRRNNNKNNKF*

Gene 2
>GAWH01047817.1 TSA: Hydractinia symbiolongicarpus Hs_transcript_47823 transcribed RNA sequence
MLVYFLLGACTILPILNNAVMGETYQRQSPGLWGRKRQPSVEADTLVQRSNDLALNKKQVEEGIWGRNVHSRI
SQQQRKQLKGRSGSGLWGREMSNNAELSLIEEKKSLENDRHMRMIKNILNLIKFYKDEGAWEMKKGVHAVKNR
YLQADGSGLWG*

Hydra magnipapillata

This sequence is identical to the GLWamide preprohormone that we cloned in 1997, https://pubmed.ncbi.nlm.nih.gov/9048780/

>GAOL01018093.1selectiontranslationframe+3
MGMFERKKIVLLVSLICVSQQATNVQDANSKSTSTELKVVKPQKRVTPVKDAEKLSILRTQDNSLDLNTNGEE
VWDELTHNIPLEYIEKIYNELNQLAQNENRPKRLWGATAAINTDNLNPEVENELENKKNAPVIEKFERPIGLW
HKDIETKNPENRLPLGLWGKDSEPLPIGLWGKDADVNDDLKKEPLPIGLWGKDTDSTRGDNKPNAYKGKLPIG
LWGKDNALTNDLGKKNNGKDSGPPPGLWGKDSKPIPGLWGKDNGPMTGLWGKKDVGPPPGLWGKKDQPPIGMW
GRAGKRDSNPYPGLWGKKEEELENVDKEIEEDSLEEFPACLLENPPCEIQEKRYNIDKSGPPPGLWGKRSEKY
QMNKPPWRGGMWGRSEILENSVHDSKKTNTIDMEHAEN*

Hydra oligactis

>GBFD01001219.1 TSA: Hydra oligactis contig15632 transcribed RNA sequence
MGMFERKKFVLLVSLICVSQQAANIKDANTLSTSTELKVVKPQKRVTPVKDAEKLSILRTQDNSLDLNANREE
VWNELTQDIPLEYIEGIYNELTRLAHNENRPKRLWGATAAINTENFNPEAENELENKKSEPVVEKFERPIGLW
HKDIETENPENRLPLGLWGKDSEPLPIGLWGKDSEVNDELNKEPLPIGLWGKDIDSTQEDNKPNPKGKLPIGL
WGKDDAVTHDLRKKNSGPPPGLWGKDKPIPGLWGKDNAPMPGLWGKKDSGPPPGLWGKKDQPPIGMWGRTGKK
DSNPYPGLWGKKEEEIENLNREFNENILEEYPPCLLENPPCEIQVKRYKTEKSGPPPGLWGKRSEKNTINKPP
WRGGMWGRSAILENSVHDSKQTNNVELKRAEKN*

Hydra vulgaris

>NW_004168100.1selectionselectiontranslationframe-1
MGMFERKKIVLLVSLICVSQQATNVQDANSKSTSTELKVVKPQKRVTPVKDAEKLSILRTQDNSLDLNTNGEE
VWDELTHNIPLEYIEKIYNELNQLAQNENRPKRLWGATAAINTDNLNPEVENELENKKNAPVIEKFERPIGLW
HKDIETKNPENRLPLGLWGKDSEPLPIGLWGKDADVNDDLKKEPLPIGLWGKDTDSTRGDNKPNAYKGKLPIG
LWGKDNALTNDLGKKNNGKDSGPPPGLWGKDSKPIPGLWGKDNGPMTGLWGKKDVGPPPGLWGKKDQPPIGMW
GRAGKRDSNPYPGLWGKKEEELENVDKEIEEDSLEEFPACLLENPPCEIQEKRYNIDKSGPPPGLWGKRSEKY
QMNKPPWRGGMWGRSEILENSVHDSKKTNTIDMEHAEN*

The above H. vulgaris sequence is identical to the H. magnipapillata sequence (see below).

MGMFERKKIVLLVSLICVSQQATNVQDANSKSTSTELKVVKPQKRVTPVKDAEKLSILRTQDNSLDLNTNGEE
VWDELTHNIPLEYIEKIYNELNQLAQNENRPKRLWGATAAINTDNLNPEVENELENKKNAPVIEKFERPIGLW
HKDIETKNPENRLPLGLWGKDSEPLPIGLWGKDADVNDDLKKEPLPIGLWGKDTDSTRGDNKPNAYKGKLPIG
LWGKDNALTNDLGKKNNGKDSGPPPGLWGKDSKPIPGLWGKDNGPMTGLWGKKDVGPPPGLWGKKDQPPIGMW
GRAGKRDSNPYPGLWGKKEEELENVDKEIEEDSLEEFPACLLENPPCEIQEKRYNIDKSGPPPGLWGKRSEKY
QMNKPPWRGGMWGRSEILENSVHDSKKTNTIDMEHAEN*

Millepora alcicornis

>GB|GFAS01111040.1| TSA: MILLEPORA ALCICORNIS TRINITY_DN83136_C0_G1_I1 TRANSCRIBED RNA SEQUENCE
LAKEENAKKHLPLNFLPMPRPPGLWANDILHRKDRNREFRSGASIPGDQYQPGLWAGDETNRIKSARFDSNLY
RELDGNSVLGGTGRDAENNSPPGLWGRGIDNHNPPGVWGRELENNSPPGLWGRDVENNNPPGVWGDSFGINRP
PGVWGREIENRNPPGVWGDVLENNSPPGLWGREIKHDAKLGNRGREIEIDTKAGVWKRDIENNSPPGLWGRDL
TQDHQTHDTNKGRKNGNN*

Millepora complanata

>GFGT01056872.1selectiontranslationframe+2
SNLYRELDGNSVLGGTGRDAENNSPPGLWGRGIDNHNPPGVWGRELENNSPPGLWGRDVENNNPPGVWGDSFG
INRPPGVWGREIENRNPPGVWGDVLENNSPPGLWGREIKHDAKLGNRGREIEIDTKAGVWKRDIENNSPPGLW
GRDLTQDHQTHDTNKGRKNGNN*

Millepora sp.

>GFGV01164755.1 TSA: MILLEPORA SP. RR-2016 TRINITY_DN97483_C0_G2_I1
TRANSCRIBED RNA SEQUENCE
MYHLFLLMVYFCMIYGEKVDLSESPKEKIEITKNDDQKNLLKRVHENVKENRELYDQLADEISKIVLKKIFDE
LKREANLEAKKELAKEENAKKHLPLNFLPMPRPPGLWANDILHRKDRNREFRSGASIPGDQYQPGLWAGDETN
RIKSARFDSNLYRELDGNSVLGGTGRDAENNSPPGLWGRGIDNHNPPGVWGRELENNSPPGLWGRDVENNNPP
GVWGDSFGINRPPGVWGREIENRNPPGVWGDVLENNSPPGLWGREIKHDAKLGNRGREIEIDTKAGVWKRDIE
NNSPPGLWGRDLTQDHQTHDTNKGRKNGNN*

Millepora squarrosa

>GFGU01143841.1selectiontranslationframe+2
MYHLFLLMVYFCMIYGEKVDLSKSPKEKIEITKNDDQKNLLKRVHENVKENRELYDQLADEISKIVLKKIFDE
LKTEANLEAKKELAKEENAKKHLPLNFLPIPRPPGLWANDILHRRDRNPEFRSGASTSGDQYQPGLWAGDETN
RIKSARFNSNLHRELDGNSVLGGIGRDAENNSPPGLWGRGIDNHNPPGVWGRELENNSPPGVWGRNVENNNPP
GVWGDSFGINRPPGVWRREIENRNPPGVWGDVSENNGPPGLWGREIKRDAKLGNHGREIEIDAKAGVWKRDIE
SYSPPGLWGRDLTQDHLTHDTNKDRKNGNN*

Physalia physalis

Gene 1
>GHBB01027127.1 TSA: PHYSALIA PHYSALIS ISOLATE YOMITAN
PHY_COMP26731_C0_SEQ1, TRANSCRIBED RNA SEQUENCE
MNDRNFIMKFLMVIWLTIVVCAMTVKGNTRGEALKELNARMLADIERKGKIMEKQRKENMELSDKTSMVEGLK
KEITEALVNILYDELKKKTNLEVLQKRIMANTLKNSDAKRPPLSLTPPPGLWVGKNDLRVSKEQWMRQEGKED
SMRADYKIRPPGLWGRELYGPPPGIWGKKEIQRSSRLWRGNREAKRSESGKQANQYINSPPGLWGKEILSTNG
DLSDANPPGLWGREMTSEEVRQPPGLWGKEVDEKTMSADIKETSDIGSSKKQN*

Gene 2
>GHBB01025771.1 TSA: PHYSALIA PHYSALIS ISOLATE YOMITAN
PHY_COMP26075_C0_SEQ1, TRANSCRIBED RNA SEQUENCE
MDIHITAFILLNLSLNDIIAANRNIRLNGNEKYEWIQAKEISEKDRSVKDNYRNLPTMFNAMKKKIMKQADGS
SLALIEFKNDPIHKGLWGKRVINLQNAMTMKSDYINSRHSNGPIENGLWGKRGDMHQSTKLLKDYDDGYVDGV
LNKGLWGKRDFIDQSTEILNNDYAGVNNYVGPLHKGLWGKREKHVRKFKGQVNNRDKKEEQSGSTKERSWDKK
SKIHHQSSRRINARFADERHWNGPIEKGLWGKRSIKDQKKRSLKDYSNGYVSGSTVNNLKSVRTVTNQGTKIL
VDRRADGRYDINAIPKGLWGKKEMMLQNARFSKDVYDENFYKNVPIEKGLWGRRNIILTNINRVESNDNGRRN
ENNPFYEMMLGKRKVIRDTQAMEKDEVSGKYDNNSLYKGLWGKRNVKSKEEKPLTIYKGDERYENDPNFKGLW
GKRTHGNKFDSAQGSFIKYNGDLGSKRSQNVDILTRRFRKDAARSIDNVYARNGDELEHNIIKKRMTDAGPV*

Podocoryna carnea

Gene 1
>GCHV01018002.1selectiontranslationframe+3
MNFQKFLSKKFMMTLGQRTLTKKTAQLPWNLESIGNGHRSKPRPPGLWGRSIHNDLEAQGPVKSFDKDAKPPG
LWGDAKPPGLWAGDAKPPGLWAGDAKPPGLWAGDSKPPGLWAGDAKPPGLWAGDAKPPGLWAGDAKTLGLKVE
DVATRGDDNSIEHDMEDKPPGLLGREATSTRLWSEGATPRSSWSKGSDSHEAKPPGLWGRDIEDSANELENKY
ARLMERRNTIEHRESDTRSSNKEYHSARGPVSSKDTFGELANILNKRIMKRLLKNGSEKHYDNGNGVKHT*

Gene 2
>GCHV01007382.1 TSA: PODOCORYNA CARNEA
PODOCORYNA_51557.0_TRANSCRIPT_1/0_CONFIDENCE_13_LENGTH_1272 TRANSCRIBED
RNA SEQUENCE
MLVYLLIGVCIILPALNEGATRETLQGQSPGLWGRKKQVTQGGVWGKRLILHKKQMDKDGVWGRDAIMEFQQG
GNEEDTFGTPELREANTFGVPELREADGISSEKRDVIRGYPERNTKLSDDVESLRTIEEMLELLKSYVVLQRE
GKLSKKGVEHSNQHLQSKLDGSGLWGR*

Porpita porpita

>GHBA01031810.1 TSA: PORPITA PORPITA ISOLATE YOMITAN
POR_COMP36469_C0_SEQ1, TRANSCRIBED RNA SEQUENCE
MMNHSMFWVVYFCAWFVQLNSAIKSTEDESTNFNDVSFKKFYDDNMNENKELYDELMKELSTKLMEKIYHKLK
RDISVDGFNDFETKQKSRRQAKPRPPGLWQGDNKNKKDSNIQGNSFEESVDETVKKDTSFRANGNENPPGGLW
GREVENNSPPGLWGRDVENNSPPGLWGRDAEVYYNDMNKKSDSDDFFEKLRHDAINSPPGLWGRDVNRNSIVA
ASEHDTENRSPPGLWGRDAEIASRTGLKVKDYEYLAEESSIENTDEKSKQISDKTSSNNDITYKYKQI*

Turritopsis

Gene 1
>IAAF01024174.1 TSA: TURRITOPSIS SP. SK-2016 MRNA, CONTIG: C43094_G1_I1,
TRANSCRIBED RNA SEQUENCE
MNKGNIKKSLEKRASALLNEIDEYHLGGLHYVPTRPRPAGLWGKRNVNRGPPGLWGRDAGNSLKRDLLKEESA
RSDRHDVVKGPPGLWGRDADTRHNTNRAAERWEREIMKNHLGKASLESKFDSNGPPGLWGRDLREKLSGRIFN
VGPPGLWGREMDINGNDMKRESERLSTNAEAVRGPPGLWGRSIKDKSHLSNRLKRDGPPGLWGRDVVVGPPGL
WGKRVVKRLLSELIENDLERLDDKSHSKP*

Gene 2
>IAAF01084686.1 TSA: TURRITOPSIS SP. SK-2016 MRNA, CONTIG: C74251_G1_I1,
TRANSCRIBED RNA SEQUENCE
MSGFNRDETNKMIGENLGSYKRENGATKGNAWTKRSLINNRDMTARYDSQKHGIWGKRMSGFNRDETNKMIGE
NLGSYKRENGATKGNAWTKRSLINNRDMTARYDSQKHGIWGKRHTGTKSDDLDKAIAEILFSYKRNADGPVIG
SGVWGKRDNKRQVNKM*

Gene 3
>IAAF01045888.1 TSA: TURRITOPSIS SP. SK-2016 MRNA, CONTIG: C54405_G1_I1,
TRANSCRIBED RNA SEQUENCE
MMVLVYVLICIISTSLTTTGARYLSAHKEIRLQNPDVEGVGLWVKRGIVQNNLKNALASENQLKEAWKRSALE
LNGGHGLWGKRQTNHGGIWGRELEDAVERKNAIFYDSSNSEEKKDIGSYWKSYVERQAKRNKMQSTGLWGK*

Velella velella

>GHAZ01086713.1 TSA: VELELLA VELELLA ISOLATE YOMITAN
VEL_COMP99114_C0_SEQ1, TRANSCRIBED RNA SEQUENCE
MNRLIFWMVYFFVGFVHSNRDVELSEEKSVNNNNADFINYYYDNVKGNKHELVNDVANILVEKIYEILKKETT
MDDLNSFKTKQNSRRQARPRPPGLWQGDGKSSTDLKNQKKSIKYLADKAVKEGTSLDAVENRNSPAGMWGRDV
ENNSPPGLWGREIINHSPPGLWGRDEESSNVDVDQKSDKMDLLKNWRREAINRSPPGLWGRDVENHSPPGLWG
RDIENNSPPGLWGKDSKYEVNEKSSENDNQEIEEVFDQISSNNDISYRNDKA*

Part Two

Polypodium hydriforme

>GBGH01006055.1selectionselectiontranslationframe+1
MASKLMLLLMAIAVVLVCASAYPGYTGKFRQIPPRDEEAQTQLKDLLRLYAEDQYEKVAR
EAYPPGLWGR*
