# Supplementary material for: An evolutionary genomics view on neuropeptide genes in Hydrozoa and Endocnidozoa (Myxozoa)
Source: BMC Genomics. 2021 Nov 30;22:862. doi: 10.1186/s12864-021-08091-2 (PMC8638164; doi:10.1186/s12864-021-08091-2)
Supplement: Supplementary file 4 — Additional file 4. Partial amino acid sequence of the LRPamide preprohormone from the hydrozoan Craspedacusta sowerbii (neuropeptide family 4). [file 12864_2021_8091_MOESM4_ESM.pdf]

Additional file 4. Partial amino acid sequence of the LRPamide preprohormone from the hydrozoan Craspedacusta sowerbii (neuropeptide family 4). The signal sequence is underlined. Neuropeptide sequences are highlighted in yellow; C-terminal processing sites are highlighted in green. The C-terminal Gly residues that are converted into C-terminal amide groups are highlighted in red.

Craspedacusta sowerbii

>QQSS01417476.1 Craspedacusta sowerbii isolate 2016 scaffold2468851_cov129, whole genome shotgun sequence
MKANTAVLTVFHMLILSPCGTVALPVADTFSSSAAEDNGQFIRPGRSESANGKDGFSRLDQRTDFERT
HRGWNGDEELDSVEKRGDFALGRNSGSAYSYADVEQVFRPANRARLAAQFLRPGKRSDNSLRPSDLGP
SDEDERMLGLLQFLRPGKRMDLIGESVTPGKRDESTLQELYDLFHVFQVLDDARRQEKLAKGISKRVS
