# Supplementary material for: An evolutionary genomics view on neuropeptide genes in Hydrozoa and Endocnidozoa (Myxozoa)
Source: BMC Genomics. 2021 Nov 30;22:862. doi: 10.1186/s12864-021-08091-2 (PMC8638164; doi:10.1186/s12864-021-08091-2)
Supplement: Supplementary file 5 — Additional file 5. Partial amino acid sequence of two RWGamide preprohormones from the hydrozoan Clytia hemisphaerica (neuropeptide family 5). [file 12864_2021_8091_MOESM5_ESM.pdf]

Additional file 5. Partial amino acid sequence of two RWGamide preprohormones from the hydrozoan Clytia hemisphaerica (neuropeptide family 5). The first preprohormone was identified by Takeda et al. in 2018 [35]. The signal sequences are underlined. Neuropeptide sequences are highlighted in yellow; C-terminal processing sites are highlighted in green. The C-terminal Gly residues that are converted into C-terminal amide groups are highlighted in red. The first neuropeptide sequence (GPPCRWGCamide) in both preprohormones must be cyclic after the formation of a cystine bridge. This sequence, however, was not recognized as a neuropeptide sequence by Takeda and coworkers [35].

Gene 1

Che-pp7:
MRFCSWTNLFLLGITCLCLTNGMPNKQHVRNKKNLIDNTVKMADHGKTLVKKSAHPMKIKDVSKKSTG
GGSDIANSDDTFDRAADGTDNSLYGRQEKEQGTENSGVGKFEGPPCRWGCGKREAGIDGPPGRWGGRK
RGMRRVGPPGRWGGRKRGELPGRWGGKKRGELPGRWGGKKRSELPGHWGGKKRSELPGHWGGKKRSEI
PGRWGGKKRSEIPGRWGGKNRSELPLGWSQKEGNQRPPSKET

Gene 2

This is a second RWGamide preprohormone fragment that we identified in the current paper. It is identical to the gene#1 product except for its C-terminus, which also includes a different neuropeptide sequence.

>TCONS_00064694-protein
MRFCSWTNLFLLGITCLCLTNGMPNKQHVRNKKNLIDNTVKMADHGKTLVKKSAHPMKIKDVSKKSTG
GGSDIANSDDTFDRAADGTDNSLYGRQEKEQGTENSGVGKFEGPPCRWGCGKREAGVDGTPGRWSGRK
RGMRRVGPPGR
