# Supplementary material for: An evolutionary genomics view on neuropeptide genes in Hydrozoa and Endocnidozoa (Myxozoa)
Source: BMC Genomics. 2021 Nov 30;22:862. doi: 10.1186/s12864-021-08091-2 (PMC8638164; doi:10.1186/s12864-021-08091-2)
Supplement: Supplementary file 6 — Additional file 6. Complete amino acid sequences of the LFRamide (neuropeptide family 6) preprohormones from three Hydra species. [file 12864_2021_8091_MOESM6_ESM.pdf]

Additional file 6. Complete amino acid sequences of the LFRamide (neuropeptide family 6) preprohormones from three *Hydra* species. Signal sequences are underlined. An asterisk indicates a stop codon. Neuropeptide sequences are highlighted in yellow; C-terminal processing sites are highlighted in green. The C-terminal Gly residues that are converted into C-terminal amide groups are highlighted in red.

Hydra magnipapillata

The cDNA for this preprohormone was cloned by Hayakawa et al.[34] (2007) DDBJ database accession # AB266099
>GAOL01004353.1selectiontranslationframe+1
MYLRLLLVVFFVLQISLQESNVRELDLGKLIEDYLAKENVRREEFLNKINTEILRYIYELENENKGKRR
IEDSADKNVLEKVLTEVPSIRESAMSKESNVNKVHNSLDSKSSIRSIPTGTLIFRGKKESNSNNENTS
EQGAPGSLLFRGKKEPYVKENSKNETEASHGERLQQTERNFLVKTKEYIEKLLNSGEEIV*

Hydra oligactis

>PJUT01444884.1selectionselectiontranslationframe-1
MYLWLLLVLFVIQGSRQEGNVRDLDLGRLIEDYLAKENVRREEFLNKINTEILRYIYELENENKEKRR
TEVNGNKNVLDKVDVTSKEIVNKQYSLDSKSNIRSIPTGTLIFRGKKESNLNNENTSEQGAPGSLLFR
GKKEPNVKENLKNETEVSHGERLQQTERNFLVKTKEYIEKLLNSGEEIV*

Hydra vulgaris

>GGKH01056607.1 TSA: Hydra vulgaris c5097_g1_i01 transcribed RNA sequence
MYLRLLLVFFVLQISLQESNVRQLDLGQLIEDYLAKENVRREEFLNKINTEILRYIYELENENKGKKR
IEASADKNVLEKVLTEVPSIRESVTSKESNVNKMHNSLDSKSSIRSIPTGTLIFRGKKESNSNNENAS
EQGAPGSLLFRGKKEPNVKENSKNETEASHGERLQQTERNFLVKTKEYIEKLLNSGEEIV*

Below is the same preprohormone from H. magnipapillata. There are 8 amino acid residue differences.
MYLRLLVVFFVLQISLQESNVRELDLGKLIEDYLAKENVRREEFLNKINTEILRYIYELENENKGKRR
IEDSADKNVLEKVLTEVPSIRESAMSKESNVNKVHNSLDSKSSIRSIPTGTLIFRGKKESNSNNENTS
EQGAPGSLLFRGKKEPYVKENSKNETEASHGERLQQTERNFLVKTKEYIEKLLNSGEEIV*
