# Supplementary material for: An evolutionary genomics view on neuropeptide genes in Hydrozoa and Endocnidozoa (Myxozoa)
Source: BMC Genomics. 2021 Nov 30;22:862. doi: 10.1186/s12864-021-08091-2 (PMC8638164; doi:10.1186/s12864-021-08091-2)
Supplement: Supplementary file 7 — Additional file 7. Complete amino acid sequences of the PKVamide neuropeptide (family 7) preprohormones from three Hydra species. [file 12864_2021_8091_MOESM7_ESM.pdf]

Additional file 7. Complete amino acid sequences of the PKVamide neuropeptide (family 7) preprohormones from three Hydra species. Signal sequences are underlined. An asterisk indicates a stop codon. Neuropeptide sequences are highlighted in yellow; C-terminal processing sites are highlighted in green. The C-terminal Gly residues that are converted into C-terminal amide groups are highlighted in red.

Hydra magnipapillata

Gene 1
This is the KVamide (also called Hym-176) preprohormone cloned in 1998 by Yum et al., doi: 10.1016/s0014-5793(98)01314-3.
>Hym176 (KVamide)
MSKINKLTMYVFYAFLVLNIYVVLSVNSLPLRDDEDTDEIDGDISELENEYQTNQVYDYNKFKNQADL
KVKSRNHYAPFIFPGPKVGRDVNFHSVLSPSDESRKSFNTYYENGYQHDKPAFLFKGYKPGDQTQKNL
*

Hydra oligactis

Gene 1
>PJUT01386048.1selectiontranslationframe+1
MSNINKLMTYVFNALLVLNIIVVLSVNSLPLRDDEDIDNEIDGDISELENEYQNNQYYDYNKFKNQAD
FKVKTRNHYAPFIFPGPKVGRDVNFHSVLSPSDESRKSNVYHINGYRQDKPSFLFKGYKPGDQTQKNF
*

Gene 2
>PJUT01035862.1selectionselectiontranslationframe+1
MSKTNKLVTYTLNAILVLNIIVVLSVNSLPLRDDEETGNEIDGDSTELENGYQSSPIYDYNMFKRQTN
LRDKDKKKIFKIFQGPKVGRDVSFHSILSLPKEIESKKSTRFYHGNGY*

Hydra vulgaris

Gene 1
>GGKH01000655.1 TSA: Hydra vulgaris c10604_g2_i01 transcribed RNA sequence
MSKINKLTMYVFYAFLVLNIYVVLSVNSLPLRDDEDTDEIDGDISELENEYQTNQVYDYNKFKNQADL
KVKSRNHYAPFIFPGPKVGRDVNFHSVLSPSDESRKSFNTYYENGYQHDKPAFLFKGYKPGDQTQKNL
*

Gene 2
This sequence appears to be an allelic variation of Hydra vulgaris gene 1. It contains an insertion (highlighted in blue) compared to Gene 1, and seven amino acid exhanges (highlighted in red).
>NW_004167320.1selectionselectiontranslationframe-1
MSKINKLTMYVFYALLVLNIYVVLSVNSLPFRDDEDTDNEIDGDISELENEYQTNQVYDYNKFKNQAD
LKIKARNHYAPFIFPGPKVGRDVNFHSVLSPSDESRKSFNNYHENGYRHDKPAFLFKGYKPGDQTQKN
L*

Gene 3
>GGKH01040744.1 TSA: Hydra vulgaris c31432_g1_i01 transcribed RNA sequence
MSKANKLTAFNILLVLNIFVILAVNSLPLRDDEEIDSEIDGDITELENGYQNTQINSYDRHKKQLNPK
DKNKKFMIFQGPKVGRDVDFHSVQSPSNKVGKSTRFYYGNDYR*
