# Supplementary material for: An evolutionary genomics view on neuropeptide genes in Hydrozoa and Endocnidozoa (Myxozoa)
Source: BMC Genomics. 2021 Nov 30;22:862. doi: 10.1186/s12864-021-08091-2 (PMC8638164; doi:10.1186/s12864-021-08091-2)
Supplement: Supplementary file 8 — Additional file 8. FASTA files used for TBLASTN. [file 12864_2021_8091_MOESM8_ESM.pdf]

Additional file 8

FASTA files used for TBLASTN (See Methods)

>sp|Q8ISH7|FCAP_APLCA Feeding circuit activating peptides OS=Aplysia californica OX=6500 PE=1 SV=1

MTFAASFRALLCVLFCAALVHCKTRTKRYVPHSELWRILAVVDELQREQAAEQRQEDALA

LALRSDIAGGGGGGQLADNVRWFPETYDYGALADRDVDKRVFDSLGGYEVHGFKKRGSLD

AIPQDTDASSDKRALDSLGGFQVHGWKRALDTLGGFQVHGWKRGSGAEKRQVDRLGGFQV

HGWKKRALDSLGGFQVHGWKKRGTGGQMHASSPRVVPWGSRSLLADTQSGHRWKRDTELV

ENRQTTGQQTEVNKRALDSLGGFQVHGWKRSGEAGKRQVDSLGGFQVHGWKRADDQGKRA

LDSLGGFQVHGWKRFDNSAGEKRALDSLGGFQVHGWKRAGDKKSLDSLGSFQVHGWKRFD

NDISGQKRSLDSLGSFQVHGWKRSDQDNKRALDSLGGFQVHGWKRADDDGKRSLDSLGSF

QVHGWKRADEDDKKSLDSLGSFQVHGWKRGDEDDKRSLDSLGSFQVHGWKRADEDDKRSL

DSLGSFQVHGWKRSDEDDKRSLDSLGSFQVHGWKRSDEDDKRSLDSLGSFQVHGWKRADE

DDKRSLDSLGSFQVHGWKRNSPGLKRALDSLGGFQVHGWKRNNEYYSGAENEKRALDSLG

GFQVHGWKRDQPGEKRSLDSLGSFQVHGWKRNLNNLGSFQVHGWKKNSADEMGDKPGVES

YQDNSGKILSGKAQEFEGGDETGDIHGVVRTLSGVDASGKERENIKELDAKFKTNDGGVG

VEHIFVDNVKSADDDVPSAGQM

>sp|P86435|VGF_BOVIN Neurosecretory protein VGF OS=Bos taurus OX=9913 GN=VGF PE=1 SV=2

MKSLRLPATVLFCLLLLIKGLGAAPPGHPEAQPPPPSSEHKEPVAGDAVLGSKDVSALEV

RAARNSEPQDEGELFQGVDPRALAAVLLQALDRPASPPAPGGSQQRPEEETAESLLTETV

RSQTHSLPVPETQAPAAPPRPQTQENGAEAPDPSEELEALASLLQELRDFSPSSAKRQQE

TAAAETETRTHTLTRVNLESPGPERVWRASWGEFQARVPERAPLPPPAPPQFQARVPESG

PLPEAHQFGGGSSPKTHLGEALAPLSKAYQGLAAPFPKARRPETSLLGGTEAGERLLQQG

LAQVEAGRRQAEATRQAAAQEERLADLASDLLLQYLLQGGARQRGLGGRGLQEEEGGGRE

TARQQEEAEQERRGGEERVGEEDEEAAEAEAEAEEAERARQNALLFAEEEEGEAGAEDKR

SREETPGHRRKEAEGAEEGGAEDEDDDEEMDPQTIDSLIELSTKLHLPADDVVSIIEEVE

EKRKRKKNAPPEPVPPPRAAPAPTHARSPKTPPPAPAPDREELPDGNEELPPRDRXENEV

FSPVPYHPFPNYIRARTVQPPPASRRRHYHHALPPSRHYPDREAQARRAQEEAEAEERRL

QEQEELENYIEHVLLRRP

>sp|P20156|VGF_RAT Neurosecretory protein VGF OS=Rattus norvegicus OX=10116 GN=Vgf PE=1 SV=3

MKTFTLPASVLFCFLLLIRGLGAAPPGRSDVYPPPLGSEHNGQVAEDAVSRPKDDSVPEV

RAARNSEPQDQGELFQGVDPRALAAVLLQALDRPASPPAVPAGSQQGTPEEAAEALLTES

VRSQTHSLPASEIQASAVAPPRPQTQDNDPEADDRSEELEALASLLQELRDFSPSNAKRQ

QETAAAETETRTHTLTRVNLESPGPERVWRASWGEFQARVPERAPLPPSVPSQFQARMSE

NVPLPETHQFGEGVSSPKTHLGETLTPLSKAYQSLSAPFPKVRRLEGSFLGGSEAGERLL

QQGLAQVEAGRRQAEATRQAAAQEERLADLASDLLLQYLLQGGARQRDLGGRGLQETQQE

RENEREEEAEQERRGGGEDEVGEEDEEAAEAEAEAEEAERARQNALLFAEEEDGEAGAED

KRSQEEAPGHRRKDAEGTEEGGEEDDDDEEMDPQTIDSLIELSTKLHLPADDVVSIIEEV

EEKRKRKKNAPPEPVPPPRAAPAPTHVRSPQPPPPAPARDELPDWNEVLPPWDREEDEVF

PPGPYHPFPNYIRPRTLQPPASSRRRHFHHALPPARHHPDLEAQARRAQEEADAEERRLQ

EQEELENYIEHVLLHRP

>sp|Q0VGU4|VGF_MOUSE Neurosecretory protein VGF OS=Mus musculus OX=10090 GN=Vgf PE=1 SV=1

MKTFTLPASVLFCFLLLIQGLGAAPPGRPDVFPPPLSSEHNGQVAEDAVSRPKDDGVPEV

RAARNPEPQDQGELFQGVDPRALASVLLQALDRPASPPSVPGGSQQGTPEEAAEALLTES

VRSQTHSLPAPEIQAPAVAPPRPQTQDRDPEEDDRSEELEALASLLQELRDFSPSNAKRQ

QETAAAETETRTHTLTRVNLESPGPERVWRASWGEFQARVPERAPLPPPVPSQFQARMSE

SAPLPETHQFGEGVSSPKTHLGETLTPLSKAYQSLGGPFPKVRRLEGSFLGGSEAGERLL

QQGLAQVEAGRRQAEATRQAAAQEERLADLASDLLLQYLLQGGARQRDLGGRELQETQQE

RENEREEEAEQERRGGGEDDVGEEDEEAAEAEAEAEEAERARQNALLFAEEEDGEAGAED

KRSQEEAPGHRRKDAEGAEEGGEEDDDDEEMDPQTIDSLIELSTKLHLPADDVVSIIEEV

EEKRKRKKNAPPEPVPPPRAAPAPTHVRSPQPPPPAPARDELPDWNEVLPPWDREEDEVF

PPGPYHPFPNYIRPRTLQPPASSRRRHFHHALPPARHHPDLEAQARRAQEEADAEERRLQ

EQEELENYIEHVLLHRP

>sp|O15240|VGF_HUMAN Neurosecretory protein VGF OS=Homo sapiens OX=9606 GN=VGF PE=1 SV=2

MKALRLSASALFCLLLLINGLGAAPPGRPEAQPPPLSSEHKEPVAGDAVPGPKDGSAPEVR

GARNSEPQDEGELFQGVDPRALAAVLLQALDRPASPPAPSGSQQGPEEEAAEALLTETVR

SQTHSLPAPESPEPAAPPRPQTPENGPEASDPSEELEALASLLQELRDFSPSSAKRQQET

AAAETETRTHTLTRVNLESPGPERVWRASWGEFQARVPERAPLPPPAPSQFQARMPDSGP

LPETHKFGEGVSSPKTHLGEALAPLSKAYQGVAAPFPKARRPESALLGGSEAGERLLQQG

LAQVEAGRRQAEATRQAAAQEERLADLASDLLLQYLLQGGARQRGLGGRGLQEAAEERES

AREEEEAEQERRGGEERVGEEDEEAAEAEAEAEEAERARQNALLFAEEEDGEAGAEDKRS

QEETPGHRRKEAEGTEEGGEEEDDEEMDPQTIDSLIELSTKLHLPADDVVSIIEEVEEKR

KRKKNAPPEPVPPPRAAPAPTHVRSPQPPPPAPAPARDELPDWNEVLPPWDREEDEVYPP

GPYHPFPNYIRPRTLQPPSALRRRHYHHALPPSRHYPGREAQARRAQEEAEAEERRLQEQ

EELENYIEHVLLRRP

>sp|P12764|ALLS_DIPPU Allatostatins OS=Diploptera punctata OX=6984 PE=1 SV=2

MSGPRTCFCLPSALVLVLLSLSTSALGTAPEPSGVHEESPAGGGTDLLPHPEDLSASDNP

DLEFVKRLYDFGLGKRAYSYVSEYKRLPVYNFGLGKRSKMYGFGLGKRDGRMYSFGLGKR

DYDYYGEEDEDDQQAIGDEDIEESDVGDLMDKRDRLYSFGLGKRARPYSFGLGKRAPSGA

QRLYGFGLGKRGGSLYSFGLGKRGDGRLYAFGLGKRPVNSGRSSGSRFNFGLGKRSDDID

FRELEEKFAEDKRYPQEHRFSFGLGKREVEPSELEAVRNEEKDNSSVHDKKNNTNDMHSG

ERIKRSLHYPFGIRKLESSYDLNSASSLNSEENDDITPEEFSRMVRRPFNFGLGKRIPMY

DFGIGKRSER

>sp|Q9W0W6|NPLP1_DROME Neuropeptide-like 1 OS=Drosophila melanogaster OX=7227 GN=Nplp1 PE=1 SV=2

MQAVLQSAHSSRRLMLLLSMLLNAAIQPRSIIVSATDDVANVSPCEMESLINQLMSPSPE

YQLHASALRNQLKNLLRERQLAVGEEQPLGEYPDYLEEDKRSVAALAAQGLLNAPKRSLA

TLAKNGQLPTAEPGEDYGDADSGEPSEQKRYIGSLARAGGLMTYGKRNVGTLARDFQLPI

PNGKRNIATMARLQSAPSTHRDPKRNVAAVARYNSQHGHIQRAGAEKRNLGALKSSPVHG

VQQKREDEEMLLPAAAPDYADPMQSYWWYPSYAGYADLDWNDYRRAEKRFLGRVLPPTRA

TASTHRSRL

>sp|Q29KK2|SNPF_DROPS Short neuropeptide F OS=Drosophila pseudoobscura pseudoobscura OX=46245 GN=sNPF PE=3 SV=2

MFRFNPQLSHGCALALICCLLNLLMMHQPTNAELSPVVQGEFFLPILPDDHPPNTDTSFG

GPISNLYDNLLQREYAGPVVFPNHQVERKAQRSPSLRLRFGRSDPDMLNNIVEKRWFGDV

NQKPIRSPSLRLRFGRRDPTLPQMRRTAYDDLLERELTLNNQQQQQLGDTADDLSADYDG

LYERVVRKPQRLRWGRSVPQFEATIGDNDQLYNSLWNSEKMRRMLLALQQYEAAPGHVAG

YANDGDDTEAQLDEDTSEFQREARKPMRLRWGRSTGKAPQIETSSIAPKN

>sp|Q9VGE8|TACHY_DROME Tachykinins OS=Drosophila melanogaster OX=7227 GN=Tk PE=1 SV=1

MRPLSGLIALALLLLLLLTAPSSAADTETESSGSPLTPGAEEPRRVVKRAPTSSFIGMRG

KKDEEHDTSEGNWLGSGPDPLDYADEEADSSYAENGRRLKKAPLAFVGLRGKKFIPINNR

LSDVLQSLEEERLRDSLLQDFFDRVAGRDGSAVGKRAPTGFTGMRGKRPALLAGDDDAEA

DEATELQQKRAPVNSFVGMRGKKDVSHQHYKRAALSDSYDLRGKQQRFADFNSKFVAVRG

KKSDLEGNGVGIGDDHEQALVHPWLYLWGEKRAPNGFLGMRGKRPALFE

>sp|Q9VIQ0|SNPF_DROME Short neuropeptide F OS=Drosophila melanogaster OX=7227 GN=sNPF PE=1 SV=4

MFHLKRELSQGCALALICLVSLQMQQPAQAEVSSAQGTPLSNLYDNLLQREYAGPVVFPN

HQVERKAQRSPSLRLRFGRSDPDMLNSIVEKRWFGDVNQKPIRSPSLRLRFGRRDPSLPQ

MRRTAYDDLLERELTLNSQQQQQQLGTEPDSDLGADYDGLYERVVRKPQRLRWGRSVPQF

EANNADNEQIERSQWYNSLLNSDKMRRMLVALQQQYEIPENVASYANDEDTDTDLNNDTS

EFQREVRKPMRLRWGRSTGKAPSEQKHTPEETSSIPPKTQN

>sp|P01210|PENK_HUMAN Proenkephalin-A OS=Homo sapiens OX=9606 GN=PENK PE=1 SV=1

MARFLTLCTWLLLLGPGLLATVRAECSQDCATCSYRLVRPADINFLACVMECEGKLPSLK

IWETCKELLQLSKPELPQDGTSTLRENSKPEESHLLAKRYGGFMKRYGGFMKKMDELYPM

EPEEEANGSEILAKRYGGFMKKDAEEDDSLANSSDLLKELLETGDNRERSHHQDGSDNEE

EVSKRYGGFMRGLKRSPQLEDEAKELQKRYGGFMRRVGRPEWWMDYQKRYGGFLKRFAEA

LPSDEEGESYSKEVPEMEKRYGGFMRF

>sp|Q9NL82|ORCKB_PROCL Orcokinin peptides type B OS=Procambarus clarkii OX=6728 PE=1 SV=1

MTAQMFTIALLLSLSAIAAAGTIKTAPARTPSTQDDASFPPDGAPVKRFDAFTTGFGHSK

RNFDEIDRSGFGFAKKNFDEIDRSGFGFNKRNFDEIDRSGFGFNKRNFDEIDRSGFGFNK

RNFDEIDRSGFGFNKRNFDEIDRSGFGFNKRNFDEIDRSGFGFNKRNFDEIDRSGFGFNK

RNFDEIDRSGFGFVKRVYVPRYIANLYKRNFDEIDRSGFGFNKRNFDEIDRTGFGFHKRD

YDVFPDKRNFDEIDRSGFGFVRRNVE

>sp|Q9NL83|ORCKA_PROCL Orcokinin peptides type A OS=Procambarus clarkii OX=6728 PE=1 SV=1

MTAQMFTIALLLSLSAIAAAGTIKTAPARTPSTQDDASFPPDGAPVKRFDAFTTGFGHSK

RNFDEIDRSGFGFAKKNFDEIDRSGFGFNKRNFDEIDRSGFGFNKRNFDEIDRSGFGFNK

RNFDEIDRSGFGFNKRNFDEIDRSGFGFNKRNFDEIDRSGFGFNKRNFDEIDRSGFGFVK

RVYVPRYIANLYKRNFDEIDRSGFGFNKRNFDEIDRTGFGFHKRDYDVFPDKRNFDEIDR

SGFGFVRRNVE

>sp|O35417|PDYN_MOUSE Proenkephalin-B OS=Mus musculus OX=10090 GN=Pdyn PE=2 SV=1

MAWSRLMLAACLLVMPSNVMADCLSLCSLCAVRIQDGPRPINPLICSLECQDLVPPSEEW

ETCRGFSSFLTLTVSGLRGKDDLEDEVALEEGISAHAKLLEPVLKELEKSRLLTSVPEEK

FRGLSSSFGNGKESELAGADRMNDEAAQGRTVHFNEEDLRKQAKRYGGFLRKYPKRSSEM

ARDEDGGQDGDQVGHEDLYKRYGGFLRRIRPKLKWDNQKRYGGFLRRQFKVVTRSQENPN

TYSEDLDV

>sp|A0SIF1|SNPF_ANOGA Short neuropeptide F OS=Anopheles gambiae OX=7165 GN=sNPF PE=1 SV=1

MYRINLTTFTLLLVLAVGSLMSESLHPSDGAINDLYEYLLQREYAAPVSYADHQIKRKAV

RSPSLRLRFGRRSDPSVPLRPEEDELIDQKAIRAPQLRLRFGRNDPLWTSFNENALLEEN

FEKRAPSQRLRWGRSNLFGNLVNQFQQDDVMQQKTIRAPQLRLRFGRTDPSWAMYNEHQL

TTGQQAQPANEASEKRAPTQRLRWGRSDPALAKDSSEDKALDVEESENTNADDK

>sp|C0HKR2|ALLA_AGRIP Allatostatin-A OS=Agrotis ipsilon OX=56364 PE=1 SV=1

MLSTSLPVCFLVIGAALCAPERMQNDPDPHDSTAQGSDNHSDHIAPLAKRSPHYDFGLGK

RAYSYVSEYKRLPVYNFGLGKRSRPYSFGLGKRSVDEDQTNDDQQQIMNNDLDQAALAEF

FDQYDDAGYEKRARPYSFGLGKRFADDDTSEEKRARAYDFGLGKRLPLYNFGLGKRARSY

NFGLGKRLASKFNFGLGKRERDMHRFSFGLGKRSADDASTEDSDNYFDV

>sp|O44314|ALLP_HELAM Helicostatins OS=Helicoverpa armigera OX=29058 PE=1 SV=1

MLYSSLPVCFLVLGAALCAPERMQNEAEPHDLQPHEAEPHSDHVAPLAKRSPHYDFGLGK

RAYSYVSEYKRLPVYNFGLGKRSRPYSFGLGKRSVDEDQSNDEQQLTTSDLDQAALAELF

DQYDDAEKRARPYSFGLGKRFADDETSEEKRARAYDFGLGKRLPMYNFGLGKRARSYNFG

LGKRYSKFNFGLGKRERDMHRFSFGLGKRSGDDVSADDSDNYFDV

>sp|A0SIX6|SNPF_AEDAE Short neuropeptide F OS=Aedes aegypti OX=7159 GN=sNPF PE=2 SV=2

MCRINFTTLSLILVLWSGSLMSEPSQNADGSIKGLYEYLLQREYAAPVSYADHQIKRKAV

RSPSLRLRFGRRSDPSVPVEPEDDDMVDQRSIRAPQLRLRFGRTDPLWSSFNENALLEEK

RAPSQRLRWGRSGGGMFSTNDVMQQKAIRAPQLRLRFGRSDPSWAMFNEHQLDEQQFADA

TRQPSKTLRGDEPTSIESTEQVESEENSPSNMDEK

>sp|Q9VVF7|MIP_DROME Allatostatins MIP OS=Drosophila melanogaster OX=7227 GN=Mip PE=1 SV=1

MAHTKTRRTYGFLMVLLILGSACGNLVASGSAGSPPSNEPGGGGLSEQVVLDQLSESDLY

GNNKRAWQSLQSSWGKRSSSGDVSDPDIYMTGHFVPLVITDGTNTIDWDTFERLASGQSA

QQQQQQPLQQQSQSGEDFDDLAGEPDVEKRAWKSMNVAWGKRRQAQGWNKFRGAWGKREP

TWNNLKGMWGKRDQWQKLHGGWGKRSQLPSN

>sp|P21786|ALLT_MANSE Allatotropin OS=Manduca sexta OX=7130 PE=1 SV=2

MNLTMQLAVIVAVCLCLAEGAPDVRLTRTKQQRPTRGFKNVEMMTARGFGKRDRPHPRAE

RDVDHQAPSARPNRGTPTFKSPTVGIARDFGKRASQYGNEEEIRVTRGTFKPNSNILIAR

GYGKRTQLPQIDGVYGLDNFWEMLETSPEREVQEVDEKTLESIPLDWFVNEMLNNPDFAR

SVVRKFIDLNQDGMLSSEELLRNF

>sp|P45644|VIP_MELGA VIP peptides OS=Meleagris gallopavo OX=9103 GN=VIP PE=2 SV=1

MEHRGTSPLLLALALLSALCWRARALPPRGAAFPAVPRLGNRLPFDAASESDRAHGSLKS

ESDILQNTLPENEKFYFDLSRIIDRNDRHADGIFTTVYSHLLAKLAVKRYLHSLIRKRVS

SQDSPVKRHSDAVFTDNYSRFRKQMAVKKYLNSVLTGKRSQEELNPAKLADEAEILEPSF

SENYDDVSVDELLSHLPLDL

>sp|P48143|VIP_CHICK VIP peptides OS=Gallus gallus OX=9031 GN=VIP PE=1 SV=1

MEHRGASPLLLALALLSALCWRARALPPRGAAFPAVPRLGNRLPFDAASESDRAHGSLKS

ESDILQNTLPENEKFYFDLSRIIDRNARHADGIFTSVYSHLLAKLAVKRYLHSLIRKRVS

SQDSPVKRHSDAVFTDNYSRFRKQMAVKKYLNSVLTGKRSQEELNPAKLRGEAEILEPSF

SENYDDVSVDELLSHLPLDL

>sp|P06850|CRF_HUMAN Corticoliberin OS=Homo sapiens OX=9606 GN=CRH PE=1 SV=1

MRLPLLVSAGVLLVALLPCPPCRALLSRGPVPGARQAPQHPQPLDFFQPPPQSEQPQQPQ

ARPVLLRMGEEYFLRLGNLNKSPAAPLSPASSLLAGGSGSRPSPEQATANFFRVLLQQLL

LPRRSLDSPAALAERGARNALGGHQEAPERERRSEEPPISLDLTFHLLREVLEMARAEQL

AQQAHSNRKLMEIIGK

>sp|A8CL69|PBAN_APIME PBAN-type neuropeptides OS=Apis mellifera OX=7460 GN=PBAN PE=1 SV=1

MIGFAVFSSFNRFTTIFVCVLLCVVYLLSYASGEYDGRDSSSGSNNDRAPSNEFGSCTDG

KCIKRTSQDITSGMWFGPRLGRRRRADRKPEINSDIEAFANAFEEPHWAIVTIPETEKRQ

ITQFTPRLGRESGEDYFSYGFPKDQEELYTEEQIYLPLFASRLGRRVPWTPSPRLGRQLH

NIVDKPRQNFNDPRF

>sp|P11159|PBAN_HELZE PBAN-type neuropeptides OS=Helicoverpa zea OX=7113 PE=1 SV=2

MFNQTQLFVFLAVFTTSSVLGNNNDVKDGAASGAHSDRLGLWFGPRLGKRSLRISTEDNR

QAFFKLLEAADALKYYYDQLPYEMQADEPETRVTKKVIFTPKLGRSLAYDDKSFENVEFT

PRLGRRLSDDMPATPADQEMYRQDPEQIDSRTKYFSPRLGRTMNFSPRLGRELSYDMMPN

KIRVVRSTNKTRST

>sp|O18641|PBAN_HELAU PBAN-type neuropeptides OS=Helicoverpa assulta OX=52344 GN=PBAN PE=2 SV=1

MFNQTQFFVLLAVFTTSSVLGNNNDVKDGAASGAHSDRLGLWFGPRLGKRSLRISTEDNR

QAFFKLLEAADALKYYYDQLPYEMQADEPETRVTKKVIFTPKLGRSLAYDDKSFENVEFT

PRLGRRLSDDMPATPADQEMYRQDPEQIDSRTKYFSPRLGRTMNFSPRLGRELSYDMMPN

KIRVVRSANKTRST

>sp|C0HL17|PBAN_HELIE PBAN-type neuropeptides OS=Heliothis peltigera OX=542982 PE=1 SV=1

MYVQRKCLFLLAVFTISSVSGNNNDVKDGADRGAHSDRGGLWFGPRLGKRSLRIATEDNR

QAFFKLLEAADALKYYYDQLPYEMQADEPETRVTKKVIFTPKLGRSIAYEDKSFENVEFT

PRLGRRLADDMPATPADQEIYRQDPEQIDSRTKYFSPRLGRTMNFSPRLGRELSYDMLPN

KIRVARSTNKTRST

>sp|O76818|PBAN_AGRIP PBAN-type neuropeptides OS=Agrotis ipsilon OX=56364 PE=1 SV=1

MYGAVLPGLFFIFISCVVASSNDVKDGGADRGAHSDRGGMWFGPRIGKRSLRMATEDNRQ

AFFKLLEAADALKYYYDQLPYEMQADEPEARVTKKVIFTPKLGRSLSYEDKMFDNVEFTP

RLGRRLADDTPATPADQEMYRPDPEQIDSRTKYFSPRLGRTMNFSPRLGRELAYEMLPSK

VRVVRSTNKTQST

>sp|P09971|PBAN_BOMMO PBAN-type neuropeptides OS=Bombyx mori OX=7091 PE=1 SV=3

MYKTNIVFNVLALALFSIFFASCTDMKDESDRGAHSERGALWFGPRLGKRSMKPSTEDNR

QTFLRLLEAADALKFYYDQLPYERQADEPETKVTKKIIFTPKLGRSVAKPQTHESLEFIP

RLGRRLSEDMPATPADQEMYQPDPEEMESRTRYFSPRLGRTMSFSPRLGRELSYDYPTKY

RVARSVNKTMDN

>sp|Q9VG55|HUGIN_DROME Protein hugin OS=Drosophila melanogaster OX=7227 GN=Hug PE=1 SV=1

MCGPSYCTLLLIAASCYILVCSHAKSLQGTSKLDLGNHISAGSARGSLSPASPALSEARQ

KRAMGDYKELTDIIDELEENSLAQKASATMQVAAMPPQGQEFDLDTMPPLTYYLLLQKLR

QLQSNGEPAYRVRTPRLGRSIDSWRLLDAEGATGMAGGEEAIGGQFMQRMVKKSVPFKPR

LGKRAQVCGGD

>sp|Q95P48|PBAN_SPOLI PBAN-type neuropeptides OS=Spodoptera littoralis OX=7109 PE=1 SV=1

MFSPLLFFAVSISCVLANSNEIKDGGSDRGAHSDRAGLWFGPRLGKRSLRISTEDNRQAF

FKLLEAADALKYYYDRLPYEMQADEPETRVTKKVIFTPKLGRSLAYDDKVFENVEFTPRL

GRRLADDMPATPADQELYRPDPDQIDSRTKYFSPRLGRTMNFSPRLGRELSYDMLPSKLR

LVRSTNRTQST

>sp|Q7PTL2|PBAN_ANOGA PBAN-type neuropeptides OS=Anopheles gambiae OX=7165 GN=PBAN PE=3 SV=2

MSRFYFFFNLICLYLAIKSALSAELDTNDQKYADLRTTGRGESPDSTGPDSDTLRRDDGA

EGLNKRAAAMWFGPRLGKRTIAADLHDDLVEEFDAEPLGYAGEPPQKLATELVQGAPYMV

LLVTAKPRKPQPIFYHTTSPRLGRRDSVGENHQRPPFAPRLGRNLPFSPRLGRSYNGGYP

LPFQFAY

>sp|Q4V4I9|CCHA1_DROME Neuropeptide CCHamide-1 OS=Drosophila melanogaster OX=7227 GN=CCHa1 PE=1 SV=1

MWYSKCSWTLVVLVALFALVTGSCLEYGHSCWGAHGKRSGGKAVIDAKQHPLPNSYGLDS

VVEQLYNNNNNNQNNQDDDNNDDDSNRNTNANSANNIPLAAPAIISRRESEDRRIGGLKW

AQLMRQHRYQLRQLQDQQQQGRGRGGQGQYDAAAESWRKLQQALQAQIDADNENYSGYEL

TK

>sp|Q24049|AMN_DROME Amnesiac neuropeptides OS=Drosophila melanogaster OX=7227 GN=amn PE=1 SV=3

MRSFCCCFYPAAVALHCVLLFYTFFLLFRASALRRRVVSGSKGSAALALCRQFEQLSASR

RERAEECRTTQLRYHYHRNGAQSRSLCAAVLCCKRSYIPRPNFSCFSLVFPVGQRFAAAR

TRFGPTLVASWPLCNDSETKVLTKWPSCSLIGRRSVPRGQPKFSRENPRALSPSLLGEMR

>sp|P18509|PACA_HUMAN Pituitary adenylate cyclase-activating polypeptide OS=Homo sapiens OX=9606 GN=ADCYAP1 PE=1 SV=3

MTMCSGARLALLVYGIIMHSSVYSSPAAAGLRFPGIRPEEEAYGEDGNPLPDFDGSEPPG

AGSPASAPRAAAAWYRPAGRRDVAHGILNEAYRKVLDQLSAGKHLQSLVARGVGGSLGGG

AGDDAEPLSKRHSDGIFTDSYSRYRKQMAVKKYLAAVLGKRYKQRVKNKGRRIAYL

>sp|Q29W19|PACA_BOVIN Pituitary adenylate cyclase-activating polypeptide OS=Bos taurus OX=9913 GN=ADCYAP1 PE=2 SV=1

MTMCSGARLALLVYGILMHSSVYGSPAASGLRFPGIRPENEVYDEDGNPQQDFYDSESLG

VGSPASALRDAYALYYPAEERDVAHGILNKAYRKVLDQPSARRSPADAHGQGLGWDPGGS

ADDDSEPLSKRHSDGIFTDSYSRYRKQMAVKKYLAAVLGKRYKQRVKNKGRRIPYL

>sp|Q13519|PNOC_HUMAN Prepronociceptin OS=Homo sapiens OX=9606 GN=PNOC PE=1 SV=1

MKVLLCDLLLLSLFSSVFSSCQRDCLTCQEKLHPALDSFDLEVCILECEEKVFPSPLWTP

CTKVMARSSWQLSPAAPEHVAAALYQPRASEMQHLRRMPRVRSLFQEQEEPEPGMEEAGE

MEQKQLQKRFGGFTGARKSARKLANQKRFSEFMRQYLVLSMQSSQRRRTLHQNGNV

>sp|P16613|PACA_SHEEP Pituitary adenylate cyclase-activating polypeptide OS=Ovis aries OX=9940 GN=ADCYAP1 PE=1 SV=1

MTMCSGARLALLVYGILMHSSVYGSPAASGLRFPGIRPENEAYDEDGNPQQDFYDSEPPG

VGSPASALRDAYALYYPAEERDVAHGILDKAYRKVLDQLSARRYLQTLMAKGLGGTPGGG

ADDDSEPLSKRHSDGIFTDSYSRYRKQMAVKKYLAAVLGKRYKQRVKNKGRRIPYL

>sp|P41535|PACA_PIG Pituitary adenylate cyclase-activating polypeptide OS=Sus scrofa OX=9823 GN=ADCYAP1 PE=1 SV=3

MTMCSGARLALLVYGIIMHSSVYCSPAAAGLRFPGIRPEDEAYDEDGNPLQDFYDSDPPG

VGGPASTLRDAYALYYPAEERDVAHGILNKAYRKVLDQLSARKYLQTLMAKSVGGNLDGG

AEDDSEPLSKRHSDGIFTDSYSRYRKQMAVKKYLAAVLGKRYKQRVKNKGRRIAYL

>sp|P13589|PACA_RAT Pituitary adenylate cyclase-activating polypeptide OS=Rattus norvegicus OX=10116 GN=Adcyap1 PE=1 SV=2

MTMCSGARLALLVYGIIMHNSVSCSPAAGLSFPGIRPEEEAYDQDGNPLQDFYDWDPPGA

GSPASALRDAYALYYPADRRDVAHEILNEAYRKVLDQLSARKYLQSMVARGMGENLAAAA

VDDRAPLTKRHSDGIFTDSYSRYRKQMAVKKYLAAVLGKRYKQRVKNKGRRIAYL

>sp|P41534|PACA_CHICK Glucagon family neuropeptides OS=Gallus gallus OX=9031 GN=ADCYAP1 PE=1 SV=2

MSGNVYKTLLTLLVYGLIMHCNVYCSPDRWTPVPGAKLEEEVYDEDGNTLQDFALRAGAP

GGGGPRPRWGRCTALYYPPGKRHADGIFSKAYRKLLGQLSARNYLHSLMAKRVGGASSGL

GDEAEPLSKRHIDGIFTDSYSRYRKQMAVKKYLAAVLGKRYKQRVKNKGRRVAYL

>sp|O70176|PACA_MOUSE Pituitary adenylate cyclase-activating polypeptide OS=Mus musculus OX=10090 GN=Adcyap1 PE=2 SV=1

MTMCSGARLALLVYGIIMHSSVSCSPAAGLSFPGIRPEDEAYDQDGNPLQDFYDWDPPGV

GSPASALRDAYALYYPADRRDVAHEILNEAYRKVLDQLSARKYLQSVVARGAGENLGGSA

VDDPAPLTKRHSDGIFTDSYSRYRKQMAVKKYLAAVLGKRYKQRVKNKGRRIAYL

>sp|Q16N80|PBAN_AEDAE PBAN-type neuropeptides OS=Aedes aegypti OX=7159 GN=PBAN PE=3 SV=1

MFRLYFFFNVICIFLAIRSAIGGEVPDATEQKINNFLASGKDSEDLSKRAAAMWFGPRLG

KRTIASELHDEMMDEIDDNPLYYSGESPQRVASEIAQGTPYVVLLLTGRVLRQPQPVFYH

STTPRLGRRDASSSNENNSRPPFAPRLGRNLPFSPRLGRSFGAPVVDNFAY

>sp|A0NDK8|CORZ_ANOGA Pro-corazonin OS=Anopheles gambiae OX=7165 GN=CRZ PE=3 SV=1

MLHTRTIALLLVGLVVLVNAQTFQYSRGWTNGKRSPLSSSSSSPSSSAAMEPLTANQLLA

SALSSGGLNSLKPSEKALLRRFLRNPCDLRVASLLAAAHPTKELFPLAGNSFDSAESAGA

AFVLPPFLMDPDESNGGIGGSNLANGRSMEDELRFKRGTATGFSDHRQKIA

>sp|P10673|NEUT_CANLF Neurotensin/neuromedin N OS=Canis lupus familiaris OX=9615 GN=NTS PE=1 SV=1

MMAGMKIQLVCMILLAFSSWSLCSDSEEEMKALEADLLTNMHTSKISKASVSSWKMTLLN

VCSFVNNLNSQAEETGEFREEELITRRKFPTALDGFSLEAMLTIYQLQKICHSRAFQQWE

LIQEDVLDAGNDKNEKEEVIKRKIPYILKRQLYENKPRRPYILKRGSYYY

>sp|P81401|VIP_BOVIN VIP peptides OS=Bos taurus OX=9913 GN=VIP PE=1 SV=2

METRSKPQLLVFLTLFSVLFSQTLAWPLFGAPSALRMGDRIPFEGANEPDQVSLKADTDI

LQDALAENDTPYYDVSRNVRHADGVFTSDYSRLLGQLSAKKYLESLIGKRVSNSISEDQG

PIKRHSDAVFTDNYTRLRKQMAVKKYLNSILNGKRSSEGESPDFLEELEK

>sp|P01283|VIP_RAT VIP peptides OS=Rattus norvegicus OX=10116 GN=Vip PE=1 SV=2

MESRSKPQFLAILTLFSVLFSQSLAWPLYGPPSSVRLDDRLQFEGAGDPDQVSLKADSDI

LQNALAENDTPYYDVSRNARHADGVFTSDYSRLLGQISAKKYLESLIGKRISSSISEDPV

PVKRHSDAVFTDNYTRLRKQMAVKKYLNSILNGKRSSEGDSPDFLEELEK

>sp|P32648|VIP_MOUSE VIP peptides OS=Mus musculus OX=10090 GN=Vip PE=1 SV=1

MEARSKPQFLAFLILFSVLFSQSLAWPLFGPPSVVRLDDRMPFEGAGDPDQVSLKADSDI

LQNPLAENGTPYYDVSRNARHADGVFTSDYSRLLGQISAKKYLESLIGKRISSSISEDPV

PIKRHSDAVFTDNYTRLRKQMAVKKYLNSILNGKRSSEGDSADFLEELEK

>sp|P01282|VIP_HUMAN VIP peptides OS=Homo sapiens OX=9606 GN=VIP PE=1 SV=1

MDTRNKAQLLVLLTLLSVLFSQTSAWPLYRAPSALRLGDRIPFEGANEPDQVSLKEDIDM

LQNALAENDTPYYDVSRNARHADGVFTSDFSKLLGQLSAKKYLESLMGKRVSSNISEDPV

PVKRHSDAVFTDNYTRLRKQMAVKKYLNSILNGKRSSEGESPDFPEELEK

>sp|P30990|NEUT_HUMAN Neurotensin/neuromedin N OS=Homo sapiens OX=9606 GN=NTS PE=1 SV=2

MMAGMKIQLVCMLLLAFSSWSLCSDSEEEMKALEADFLTNMHTSKISKAHVPSWKMTLLN

VCSLVNNLNSPAEETGEVHEEELVARRKLPTALDGFSLEAMLTIYQLHKICHSRAFQHWE

LIQEDILDTGNDKNGKEEVIKRKIPYILKRQLYENKPRRPYILKRDSYYY

>sp|P01156|NEUT_BOVIN Neurotensin/neuromedin N OS=Bos taurus OX=9913 GN=NTS PE=1 SV=3

MMAGMKIQLVCMILLAFSSWSLCSDSEEEMKALETDLLTNMHTSKISKASVPSWKMSLLN

VCSLINNLNSQAEETGEFHEEELITRRKFPAALDGFSLEAMLTIYQLQKICHSRAFQHWE

LIQEDILDAGNDKNEKEEVIKRKIPYILKRQLYENKPRRPYILKRGSYYY

>sp|Q9D3P9|NEUT_MOUSE Neurotensin/neuromedin N OS=Mus musculus OX=10090 GN=Nts PE=2 SV=1

MRGMNLQLVCLTLLAFSSWSLCSDSEEDVRALEADLLTNMHTSKISKASPPSWKMTLLNV

CSLINNVNSPAEEAGDMHDDDLVGKRKLPLVLDGFSLEAMLTIFQLQKICRSRAFQHWEI

IQEDILDNVNDKNEKEEVIKRKIPYILKRQLYENKPRRPYILKRGSYYY

>sp|P20068|NEUT_RAT Neurotensin/neuromedin N OS=Rattus norvegicus OX=10116 GN=Nts PE=1 SV=1

MIGMNLQLVCLTLLAFSSWSLCSDSEEDVRALEADLLTNMHASKVSKGSPPSWKMTLLNV

CSLINNLNSAAEEAGEMRDDDLVAKRKLPLVLDDFSLEALLTVFQLQKICRSRAFQHWEI

IQEDILDHGNEKTEKEEVIKRKIPYILKRQLYENKPRRPYILKRASYYY

>sp|P35455|NEU2_MOUSE Vasopressin-neurophysin 2-copeptin OS=Mus musculus OX=10090 GN=Avp PE=2 SV=1

MLARMLNTTLSACFLSLLAFSSACYFQNCPRGGKRAISDMELRQCLPCGPGGKGRCFGPS

ICCADELGCFVGTAEALRCQEENYLPSPCQSGQKPCGSGGRCAAVGICCSDESCVAEPEC

HDGFFRLTRAREPSNATQLDGPARALLLRLVQLAGTRESVDSAKPRVY

>sp|P01186|NEU2_RAT Vasopressin-neurophysin 2-copeptin OS=Rattus norvegicus OX=10116 GN=Avp PE=1 SV=1

MLAMMLNTTLSACFLSLLALTSACYFQNCPRGGKRATSDMELRQCLPCGPGGKGRCFGPS

ICCADELGCFLGTAEALRCQEENYLPSPCQSGQKPCGSGGRCAAAGICCSDESCVAEPEC

REGFFRLTRAREQSNATQLDGPARELLLRLVQLAGTQESVDSAKPRVY

>sp|P01183|NEU2_PIG Vasopressin-neurophysin 2-copeptin OS=Sus scrofa OX=9823 GN=AVP PE=1 SV=2

MPDATLPACFLGLLALTSACYFQNCPKGGKRAMSDLELRQCLPCGPGGKGRCFGPSICCG

DELGCFVGTAEALRCQEENYLPSPCQSGQKPCGSGGRCAAAGICCNDESCVTEPECREGA

SFLRRARASDRSNATLLDGPSGALLLRLVQLAGAPEPAEPAQPGVY

>sp|P01180|NEU2_BOVIN Vasopressin-neurophysin 2-copeptin OS=Bos taurus OX=9913 GN=AVP PE=1 SV=1

MPDATLPACFLSLLAFTSACYFQNCPRGGKRAMSDLELRQCLPCGPGGKGRCFGPSICCG

DELGCFVGTAEALRCQEENYLPSPCQSGQKPCGSGGRCAAAGICCNDESCVTEPECREGV

GFPRRVRANDRSNATLLDGPSGALLLRLVQLAGAPEPAEPAQPGVY

>sp|P01185|NEU2_HUMAN Vasopressin-neurophysin 2-copeptin OS=Homo sapiens OX=9606 GN=AVP PE=1 SV=2

MPDTMLPACFLGLLAFSSACYFQNCPRGGKRAMSDLELRQCLPCGPGGKGRCFGPSICCA

DELGCFVGTAEALRCQEENYLPSPCQSGQKACGSGGRCAAFGVCCNDESCVTEPECREGF

HRRARASDRSNATQLDGPAGALLLRLVQLAGAPEPFEPAQPDAY

>sp|Q924A4|UCN3_MOUSE Urocortin-3 OS=Mus musculus OX=10090 GN=Ucn3 PE=2 SV=1

MLMPTYFLLPLLLLLGGPRTSLSHKFYNTGPVFSCLNTALSEVKKNKLEDVPLLSKKSFG

HLPTQDPSGEEDDNQTHLQIKRTFSGAAGGNGAGSTRYRYQSQAQHKGKLYPDKPKSDRG

TKFTLSLDVPTNIMNILFNIDKAKNLRAKAAANAQLMAQIGKKK

>sp|Q299B0|CORZ_DROPS Pro-corazonin OS=Drosophila pseudoobscura pseudoobscura OX=46245 GN=Crz PE=3 SV=1

MMRLLLLPLFLFTLSMACMGQTFQYSRGWTNGKRALTPPSLLSHGHFNRASDLGFSDLYD

VQDWSSERRLERCLAQLQRSLLSRVYGSVVDFNANRPEPDSSDSGSSRNRANNNNENVLY

PTPIQNRHHSSNELLEEISAAVAGSGPTGAGSGEPSVFGKH

>sp|P24787|NEUV_CHICK Vasotocin-neurophysin VT OS=Gallus gallus OX=9031 PE=2 SV=1

MAEPSLPLSFLCLLALSSACYIQNCPRGGKRALGDTALRQCLPCGPGNRGRCFGPGICCG

AELGCYLGTAETRRCAEEDYMPSPCQAGGQPCGSDGRCAANGVCCSADTCAMDAVCLEEG

SEQAEEAAEKNLTVLDGAAGDLLLRLMHLANRQQQGKQPGL

>sp|Q7M428|NEU3_EPTST Vasotocin-neurophysin VT OS=Eptatretus stoutii OX=7765 PE=2 SV=1

MSAMGWTLLAAALLAISAQSNGCYIQNCPRGGKRAVETELHSCAACGLGGQCVGPSICCG

GLLGGGRGGGCIVGGPLSAPCKRENLHPEPCRPGGGSSCGLEGICAAPGICCTDVTCSID

ATCDDVTEKAGVTFSGATGGLANPTGDLLRKVLLLANADLE

>sp|A0A4Y5X186|CESS2_CONMO Conopressin/conophysin, isoform 2 (Fragment) OS=Conus monile OX=351660 PE=2 SV=1

MKCSVLQMSRLSWAMCLMLLMLLLLGTAQGCFIRNCPRGGKRAVDALQPTRQCMSCGPDG

VGQCVGPSVCCGLGLGCLMGTPETEVCQKENESSVPCAISGRHCGMDNTGNCVADGICCV

EDACSFNSLCRVDTDQEDSVSARQELLTLIRRLLVNRQYD

>sp|P81829|LCK_DROME Leucokinin OS=Drosophila melanogaster OX=7227 GN=Lk PE=1 SV=3

MAKIVLCMVLLAFGRQVYGASLVPAPISEQDPELATCELQLSKYRRFILQAILSFEDVCD

AYSSRPGGQDSDSEGWPFRHYAPPPTSQRGEIWAFFRLLMAQFGDKEFSPIIRDAVIERC

RIKSQLQRDEKRNSVVLGKKQRFHSWGGKRSPEPPILPDY

>sp|P08163|NEUV_BUFJA Vasotocin-neurophysin VT (Fragment) OS=Bufo japonicus OX=8387 PE=2 SV=1

TAPVPACFLCLLALSSACYIQNCPRGGKRSYPDTAVRQCIPCGPGNRGNCFGPNICCGED

LGCYVGTPETLRCVEETYLPSPCEAGGKPCSSGGRCAAPGVCCSDDTCVVDSSCLDEDSE

RRRVTPEQNMTQMDGSASDLLLRLMHMANRQQQSKHQFY

>sp|P16042|NEU4_ONCKE Vasotocin-neurophysin VT 2 OS=Oncorhynchus keta OX=8018 PE=2 SV=1

MPHSTLLLCVIGLLAFSSACYIQNCPRGGKRALQDTGIRQCMTCGPGDQGHCFGPSICCG

EGLGCWMGSPETARCFEENYLPTPCQTGGRPCGSDAGRCAAPGVCCDSESCVLDPDCLSE

SRYHSPADHSAGATSDSPGELLLRLLHFATRGQSEYKQ

>sp|C0HKS6|CAPA_AGRIP CAPA peptides OS=Agrotis ipsilon OX=56364 PE=1 SV=1

MQPTMRIIVSMALLAYAVASAYHSNVKLRRDGKMVLYPFPRVGRASGNTWQLPLNDLYPE

YEPAQVKRQLYAFPRVGRDPVMSRLGRSDLSRVESHEFQPMAVRRTESPGMWFGPRLGRA

FKNDDDEITIQNESNDHSEPEQTELIHEDRRKRQTLN

>sp|Q91166|NEU1_ONCKE Isotocin-neurophysin IT 1 OS=Oncorhynchus keta OX=8018 PE=2 SV=2

MFGTSVSALCLLFLLSVCTACYISNCPIGGKRSALAFPSRKCMACGPGDRGRCFGPNICC

GEGMGCYVGSPEAAGCVEENYLPSPCEAGGRVCGSEEGRCAAPGICCDVEGCSIDQSCTE

EDEAEYISQSVSSSHGHDLLMKLLNMISHTPPHRVHK

>sp|Q07663|NEU1_ONCMA Isotocin-neurophysin IT 1 OS=Oncorhynchus masou OX=8020 PE=1 SV=2

MFGTSVSALCLLFLLSVCTACYISNCPIGGKRSALAFPSRKCMSCGPGDRGRCFGPNICC

GEGMGCYVGSPEAAGCVEENYLPSPCEVGGRVCGSEEGRCAAPGICCDVEGCSIDQSCTE

EDEAEYISQSVSSSHGHDLLMKLLNMISHTPPHRVHK

>sp|Q91167|NEU2_ONCKE Isotocin-neurophysin IT 2 OS=Oncorhynchus keta OX=8018 PE=2 SV=1

MTGAAVSVCLLYALSVCSACYISNCPIGGKRSIMDAPQRKCMSCGPGEQGRCFGPSICCG

KDVGCWMGSPETAHCMEENYLPTPCQVGGRPCGSDTVRCASPGVCCDSEGCSADQSCFAE

EEGDNQIGQSEGSNSADVILRLLHLADHTPPHRVHQ

>sp|O42493|NEUI_TAKRU Isotocin-neurophysin IT 1 OS=Takifugu rubripes OX=31033 PE=3 SV=1

MTGTAISVCLLFLLSVCSACYISNCPIGGKRSIMDAPQRKCMSCGPGDRGRCFGPGICCG

ESFGCLMGSPESARCAEENYLLTPCQAGGRPCGSEGGLCASSGLCCDAESCTMDQSCLSE

EEGDERGSLFDGSDSGDVILKLLRLAGLTSPHQTH

>sp|Q07662|NEU3_ONCMA Vasotocin-neurophysin VT 1 OS=Oncorhynchus masou OX=8020 PE=1 SV=1

MPDSTIPLLCVLGLLALSSACYIQNCPRGGKRSFPDLKRPCMSCGPGNRGLCFGPSICCG

EGMGCYMGSPEAASCVEENYLTSPCEVGGRVCGSEEGHCAAPGVCCDAESCLLDSDCLDD

SKRQPPSEQYSSLMEGLAGDLLQWMLHATRRERPQ

>sp|Q00945|CONO_LYMST Conopressin/neurophysin OS=Lymnaea stagnalis OX=6523 PE=1 SV=1

MMSSLCGMPLTYLLTAAVLSLSLTDACFIRNCPKGGKRSLDTGMVTSRECMKCGPGGTGQ

CVGPSICCGQDFGCHVGTAEAAVCQQENDSSTPCLVKGEACGSRDAGNCVADGICCDSES

CAVNDRCRDLDGNAQANRGDLIQLIHKLLKVRDYD

>sp|Q9VCW0|CCAP_DROME Cardioactive peptide OS=Drosophila melanogaster OX=7227 GN=CCAP PE=1 SV=2

MRTSMRISLRLLALLACAICSQASLERENNEGTNMANHKLSGVIQWKYEKRPFCNAFTGC

GRKRTYPSYPPFSLFKRNEVEEKPYNNEYLSEGLSDLIDINAEPAVENVQKQIMSQAKIF

EAIKEASKEIFRQKNKQKMLQNEKEMQQLEERESK

>sp|Q29CA0|CP2B_DROPS Cardio acceleratory peptide 2b OS=Drosophila pseudoobscura pseudoobscura OX=46245 GN=capa PE=3 SV=2

MKAIFSLYNIVSAILLLVLLAEFSTAELNHDKNRRGANMGLYAFPRVGRSDPSLANSLRD

ASDAAVFDGLYGDASQEDYNEADYQKRAGLVAFPRVGRSDAELRKFAHLLALQQVLDKRT

GPSASSGLWFGPRLGKRSVDAKAFSDASKGQQEFN

>sp|P16229|NEU4_CATCO Vasotocin-neurophysin VT 2 OS=Catostomus commersonii OX=7971 PE=2 SV=1

MSVCAVLLLCVAGLLCLSSACYIQNCPRGGKRALLEPVSRQCLACGPGDKGRCLGPSICC

GEEIGCLVGSPWMARCQEEEYLPSPCQTAGKLCGSDAGPCAAPGVCCGTEGCKLDPNCSE

DSESEEPADQNTLGASPGELLLRLLHPNNRKHNQY

>sp|O45027|PBAN_MAMBR PBAN-type neuropeptides (Fragment) OS=Mamestra brassicae OX=55057 GN=PBAN PE=2 SV=1

GLWFGPRIGKRSLRMATEDNRQAFFKLLEAADALKYYYDQLPYEMQADEPETRVTKKVIF

TPKLGRSLAYDDKVFENVEFTPRLGRRLADDMPATPADQEMYRPDPEQIDSRTKYFSPRL

GRTMNFSPRLGRELAYEMVPSKIRVVRSTNKTQST

>sp|P29007|GRP_BOMOR Gastrin-releasing peptide OS=Bombina orientalis OX=8346 GN=grp PE=1 SV=1

MEGVLLFWKYRALFFLVLCSLVLCKVHLSQASPTSQQHNDAASLSKIYPRGSHWAVGHLM

GKKSIEEYPYAYDEADRSSAAVFSEGDKPSDGYQQWKESLLNLLKMIEVNEYRNSKAMRE

ASVYNKKFSGAEDNNLKEMLDYLYQMMNMKENTSS

>sp|Q5W1L5|CORZ_DROSI Pro-corazonin OS=Drosophila simulans OX=7240 GN=Crz PE=2 SV=1

MLRLLLLPLFLFTLSMCMGQTFQYSRGWTNGKRSFNAASPLLANGHLHRGSELGLTDLYD

LQDWSSDRRLERCLSQLQRSLIARNCVPGSDFNANRVDPDPENSVHPRLSNINGENVLYS

SANIPNRHRQSNELLEELSAAGGASAEPNVFGKH

>sp|Q26377|CORZ_DROME Pro-corazonin OS=Drosophila melanogaster OX=7227 GN=Crz PE=1 SV=2

MLRLLLLPLFLFTLSMCMGQTFQYSRGWTNGKRSFNAASPLLANGHLHRASELGLTDLYD

LQDWSSDRRLERCLSQLQRSLIARNCVPGSDFNANRVDPDPENSAHPRLSNSNGENVLYS

SANIPNRHRQSNELLEELSAAGGASAEPNVFGKH

>sp|Q5W1L4|CORZ_DROER Pro-corazonin OS=Drosophila erecta OX=7220 GN=Crz PE=2 SV=1

MLRLLLLPLFLFTLSMCMGQTFQYSRGWTNGKRSFNAASPLLTTGHLHRGSELGLSDLYD

LQEWTSDRRLERCLSQLQRSLIARNCVPGSDFNANRVDPDPESSAHPRLGNINNENVLYS

SANVPTRHRQSNELLEELSAAGGASAEPNVFGKH

>sp|P15210|NEU1_CATCO Isotocin-neurophysin IT 1 OS=Catostomus commersonii OX=7971 PE=2 SV=1

MSGSMFSVFSLLYLLSVCSACYISNCPIGGKRAIQDSPSRQCMSCGPGDRGRCFGPSICC

GEGLGCLLGSPETQRCLEEDFLPSPCEAGGKVCGYEGRCAAPGVCCDSEGCSVDQSCVDG

DGDATAVSQPASSQDLLLKLLHLSNPAHPYRLHQ

>sp|Q566B3|PBURS_ANOGA Partner of bursicon OS=Anopheles gambiae OX=7165 GN=pburs PE=2 SV=1

MCNSVRTALAASNCCSIVLCCVLLLTLTLTVAVTAQHNQADETCETLPSEIHLIKEEYDE

LGRLYRTCNGDVTVNKCEGKCNSQVQPSVITATGFLKECYCCRESFLRERQLQLTHCYDP

DGVRMTDHESATMEIRLKEPVDCKCFKCGEMVR

>sp|O42499|NEUV_TAKRU Vasotocin-neurophysin VT 1 OS=Takifugu rubripes OX=31033 PE=3 SV=1

MPQCALLLSLLGLLALSSACYIQNCPRGGKRALPETGIRQCMSCGPRDRGRCFGPNICCG

EALGCLMGSPETARCAGENYLLTPCQAGGRPCGSEGGRCAVSGLCCNSESCAVDSDCLGE

TESLEPGDSSADSSPTELLLRLLHMSSRGQSEY

>sp|Q5W1L3|CORZ_DROVI Pro-corazonin OS=Drosophila virilis OX=7244 GN=Crz PE=2 SV=1

MLRLLLLPLFLFTLSMACMGQTFQYSRGWTNGKRAPPAALVTNGHNLGLLDIYDIQDRPT

DIKLERCLLQLQHFVGNALLHRSFANGLAYSASRPDPETDVRSINIHSRPGSGNNNIENS

LYPNVNHRQSNELFEALNAPGPDAVEPNDYGKH

>sp|P16041|NEU3_ONCKE Vasotocin-neurophysin VT 1 OS=Oncorhynchus keta OX=8018 PE=2 SV=1

MPYSTFPLLWVLGLLALSSACYIQNCPRGGKRSFPDLPRQCMSCGPGDRGRCFGPNICCG

EGMGCYMGSPEAAGCVEENYLPSPCEAGGRVCGSEGSCAASGVCCDSESCVLDPDCLEDS

KRQSPSEQNAALMGGLAGDLLRILHATSRGRPQ

>sp|P17668|NEU3_CATCO Vasotocin-neurophysin VT 1 OS=Catostomus commersonii OX=7971 PE=2 SV=1

MSDSFLPTCILCLLALSSACYIQNCPRGGKRSQPDTSRECVSCGPGNAGRCYGPSICCGA

ALGCLVGSPETMSCMEENHLPSPCETGGRPCGDEGRCAAPGVCCDSVSCVMDSECLEDVR

SDQSEDPSRLKTVSGEILLRLLNLASRGRRDF

>sp|P01161|ANF_RAT Natriuretic peptides A OS=Rattus norvegicus OX=10116 GN=Nppa PE=1 SV=1

MGSFSITKGFFLFLAFWLPGHIGANPVYSAVSNTDLMDFKNLLDHLEEKMPVEDEVMPPQ

ALSEQTDEAGAALSSLSEVPPWTGEVNPSQRDGGALGRGPWDPSDRSALLKSKLRALLAG

PRSLRRSSCFGGRIDRIGAQSGLGCNSFRYRR

>sp|P05125|ANF_MOUSE Natriuretic peptides A OS=Mus musculus OX=10090 GN=Nppa PE=1 SV=2

MGSFSITLGFFLVLAFWLPGHIGANPVYSAVSNTDLMDFKNLLDHLEEKMPVEDEVMPPQ

ALSEQTEEAGAALSSLPEVPPWTGEVNPPLRDGSALGRSPWDPSDRSALLKSKLRALLAG

PRSLRRSSCFGGRIDRIGAQSGLGCNSFRYRR

>sp|P01160|ANF_HUMAN Natriuretic peptides A OS=Homo sapiens OX=9606 GN=NPPA PE=1 SV=2

MSSFSTTTVSFLLLLAFQLLGQTRANPMYNAVSNADLMDFKNLLDHLEEKMPLEDEVVPP

QVLSEPNEEAGAALSPLPEVPPWTGEVSPAQRDGGALGRGPWDSSDRSALLKSKLRALLT

APRSLRRSSCFGGRMDRIGAQSGLGCNSFRY

>sp|Q9VC44|ALLS_DROME Allatostatin-A OS=Drosophila melanogaster OX=7227 GN=AstA PE=1 SV=1

MNSLHAHLLLLAVCCVGYIASSPVIGQDQRSGDSDADVLLAADEMADNGGDNIDKRVERY

AFGLGRRAYMYTNGGPGMKRLPVYNFGLGKRSRPYSFGLGKRSDYDYDQDNEIDYRVPPA

NYLAAERAVRPGRQNKRTTRPQPFNFGLGRR

>sp|Q9NIP6|CP2B_DROME Cardio acceleratory peptide 2b OS=Drosophila melanogaster OX=7227 GN=Capa PE=1 SV=1

MKSMLVHIVLVIFIIAEFSTAETDHDKNRRGANMGLYAFPRVGRSDPSLANSLRDGLEAG

VLDGIYGDASQEDYNEADFQKKASGLVAFPRVGRGDAELRKWAHLLALQQVLDKRTGPSA

SSGLWFGPRLGKRSVDAKSFADISKGQKELN

>sp|P07492|GRP_HUMAN Gastrin-releasing peptide OS=Homo sapiens OX=9606 GN=GRP PE=1 SV=2

MRGRELPLVLLALVLCLAPRGRAVPLPAGGGTVLTKMYPRGNHWAVGHLMGKKSTGESSS

VSERGSLKQQLREYIRWEEAARNLLGLIEAKENRNHQPPQPKALGNQQPSWDSEDSSNFK

DVGSKGKVGRLSAPGSQREGRNPQLNQQ

>sp|P15211|NEU2_CATCO Isotocin-neurophysin IT 2 OS=Catostomus commersonii OX=7971 PE=2 SV=1

MSGSMSSVFSLLYLLSVCSACYISNCPIGGKRAVQDLPSRQCMSCGPGDRGRCFGPSICC

GEGLGCLLGSPETLRCQEEDFLPSPCEAGGKMCGYEGRCAAPGVCCDSEGCSMDQSCVNG

DATAFGQPDLLLKLLHLSNHAHPYRLHQ

>sp|Q99LS0|AUGN_MOUSE Augurin OS=Mus musculus OX=10090 GN=Ecrg4 PE=1 SV=2

MSTSSARPAVLALAGLALLLLLCLGPDGISGNKLKKMLQKREGPVPSKTNVAVAENTAKE

FLGGLKRAKRQLWDRTRPEVQQWYQQFLYMGFDEAKFEDDVNYWLNRNRNGHDYYGDYYQ

RHYDEDAAIGPHSRESFRHGASVNYNDY

>sp|Q9H1Z8|AUGN_HUMAN Augurin OS=Homo sapiens OX=9606 GN=ECRG4 PE=1 SV=1

MAASPARPAVLALTGLALLLLLCWGPGGISGNKLKLMLQKREAPVPTKTKVAVDENKAKE

FLGSLKRQKRQLWDRTRPEVQQWYQQFLYMGFDEAKFEDDITYWLNRDRNGHEYYGDYYQ

RHYDEDSAIGPRSPYGFRHGASVNYDDY

>sp|P01181|NEU2_SHEEP Vasopressin-neurophysin 2-copeptin (Fragment) OS=Ovis aries OX=9940 GN=AVP PE=1 SV=1

CYFQNCPRGXXXAMSDLELRQCLPCGPGGKGRCFGPSICCGDELGCFVGTAEALRCQEEI

YLPSPCQSGQKPCGSGGRCAAAGICCNDESCVTEPECREGIGFPRRVXASDRSNATLLDG

PSGALLLRLVQLAAAPEPAEPAQPGVY

>sp|P24393|GRP_RAT Gastrin-releasing peptide OS=Rattus norvegicus OX=10116 GN=Grp PE=2 SV=2

MRGSELSLLLLALVLCQAPRGPAAPVSTGAGGGTVLAKMYPRGSHWAVGHLMGKKSTDEL

PPLYAADRDGLKEQLRGYIRWEEAARNLLGLLEAAGNRSHQPPQDQPLGSLQPTWDPEDG

SYFSDAQNAKLVDSLLQVLKGKEGTAS

>sp|Q8R1I2|GRP_MOUSE Gastrin-releasing peptide OS=Mus musculus OX=10090 GN=Grp PE=1 SV=1

MRGSELSLLLLALVLCQAPRGPAAPVSTGAGGGTVLAKMYPRGSHWAVGHLMGKKSTDES

PSLYAADRDGLKEQLRGYVRWEEAARDLLDLLEAAGNQSHQPPQHPPLSLQPTWDPEDGS

YFNDVQTAKLVDSLLQVLKEKGGTAS

>sp|A2VB90|PBURS_APIME Partner of bursicon OS=Apis mellifera OX=7460 GN=pburs PE=2 SV=1

MKENFSIMFIHSIFLILIIFIYSNETIAQVTDDENCETLQSEVHITKDEYDEIGRLKRTC

SGDISVTKCEGFCNSQVQPSVASTTGFSKECYCCRESYLKERHITLHHCYDADGIKLMNE

ENGVMEIKIREPVECKCIKCGDISQ

>sp|P10769|NEU2_CAVPO Vasopressin-neurophysin 2-copeptin OS=Cavia porcellus OX=10141 GN=AVP PE=1 SV=2

CYFQNCPRGGKRALSDTELRQCLPCGPGGQGRCFGPSICCADALGCFVGTAEALRCQEEN

YLPSPCQSGQKPCGSGGRCAANGVCCNDESCVIEPECREEFHRPVRAGDRSNVTQLDGPA

GALLLRLMQLAGAPEPQPAAPGGY

>sp|P14944|CHH_CARMA Crustacean hyperglycemic hormones OS=Carcinus maenas OX=6759 PE=1 SV=2

MYSKTIPAMLAIITVAYLCALPHAHARSTQGYGRMDRILAALKTSPMEPSAALAVENGTT

HPLEKRQIYDTSCKGVYDRALFNDLEHVCDDCYNLYRTSYVASACRSNCYSNLVFRQCMD

DLLMMDEFDQYARKVQMVGRKK

>sp|B2ZB99|DSK_DROSI Drosulfakinins OS=Drosophila simulans OX=7240 GN=Dsk PE=1 SV=2

MGLRSCTHLATLFMTLWAVAFCFLVVVPIPAQTTSLQNAKDDRRLQELESKIGAESDQTN

ANLVGPSFSRFGDRRNQKAISFGRRVPLISRPMIPIELDLLMDNDDERTKAKRFDDYGHM

RFGKRGGDDQFDDYGHMRFGR

>sp|P09040|DSK_DROME Drosulfakinins OS=Drosophila melanogaster OX=7227 GN=Dsk PE=1 SV=2

MGPRSCTHFATLFMPLWALAFCFLVVLPIPAQTTSLQNAKDDRRLQELESKIGGEIDQPI

ANLVGPSFSLFGDRRNQKTMSFGRRVPLISRPIIPIELDLLMDNDDERTKAKRFDDYGHM

RFGKRGGDDQFDDYGHMRFGR

>sp|B2ZB95|DSK_DROER Drosulfakinins OS=Drosophila erecta OX=7220 GN=Dsk PE=1 SV=1

MGLRRCTHFATLVMPLWALALFFLVVMQVPAQTTSLQISKEDRRLQELESKMGAESEQPN

ANLVGPSISRFGDRRNQKTISFGRRVPLISRPMIPIELDLLMDNDDERTKAKRFDDYGHM

RFGKRGGDDQFDDYGHMRFGR

>sp|B2ZB98|DSK_DROSE Drosulfakinins OS=Drosophila sechellia OX=7238 GN=Dsk PE=1 SV=1

MGLRSCTHLATLFMTLWALAFCFLVVVPIPAQTTSLQNAKDDRRLQELESKIGAESDQPN

ANLVGPSFSRFGDRRNQKTISFGRRVPLISRPMIPIELDLLMDNDDERTKAKRFDDYGHM

RFGKRGGDDQFDDYGHMRFGR

>sp|Q9VJS7|PBURS_DROME Partner of bursicon OS=Drosophila melanogaster OX=7227 GN=Pburs PE=1 SV=1

MHVQELLFVAAILVPQCLRALRYSQGTGDENCETLKSEIHLIKEEFDELGRMQRTCNADV

IVNKCEGLCNSQVQPSVITPTGFLKECYCCRESFLKEKVITLTHCYDPDGTRLTSPEMGS

MDIRLREPTECKCFKCGDFTR

>sp|P11858|NEUV_PELLE Vasotocin-neurophysin VT OS=Pelophylax lessonae OX=45623 PE=1 SV=1

CYIQNCPRGGKRSYPDTEVRQCIPCGPGNRGNCFGPNICCGEDLGCYIGTPETLRCVEEN

YLPSPCEAGGKPCGAGGRCAAPGVCCNDQSCTMDSSCLDEDSERQRVSPDQNMTQMNGSA

SDLLLRLMHMANRQQQQTKHY

>sp|Q566B2|PBURS_BOMMO Partner of bursicon OS=Bombyx mori OX=7091 GN=pburs PE=2 SV=1

MNIMITKIFFLVQLFYIVVSKSSAEENCETVASEVHVTKEEYDEMGRLLRSCSGEVSVNK

CEGMCNSQVHPSISSPTGFQKECFCCREKFLRERLVTLTHCYDPDGIRFEDEENALMEVR

LREPDECECYKCGDFSR

>sp|P56688|MOIH_LIBEM Mandibular organ-inhibiting hormone OS=Libinia emarginata OX=6807 PE=1 SV=1

MTTKCTVMAVVLAACICLQVLPQAYGRSTQGYGRMDKLLATLMGSSEGGALESASQHSLE

KRQIFDPSCKGLYDRGLFSDLEHVCKDCYNLYRNPQVTSACRVNCYSNRVFRQCMEDLLL

MEDFDKYARAIQTVGKK

>sp|Q17AN4|CORZ_AEDAE Pro-corazonin OS=Aedes aegypti OX=7159 GN=Crz PE=3 SV=1

MKHVFSTSLIVSLFVIFTDAQTFQYSRGWTNGKRSSPEQTAPSRTLLPHIPLGMDKPDEE

CRLLIQRFLKSPCDVRLANAIVNRNKDLLRDMADDVNDGTALLYDPVPMVDTAASEDVRF

KRGTPDRRLLNDGMHRL

>sp|Q25683|CHH_PROCL Crustacean hyperglycemic hormones OS=Procambarus clarkii OX=6728 PE=1 SV=3

MVSFRTMWSLVVVVVVVAASLGSSGVHGRSVEGSSRMERLLSSGSSSSEPLSFLSQDQSV

NKRQVFDQACKGIYDRAIFKKLDRVCEDCYNLYRKPYVATTCRQNCYANSVFRQCLDDLL

LIDVVDEYISGVQTVGK

>sp|Q8SXL2|CCHA2_DROME Neuropeptide CCHamide-2 OS=Drosophila melanogaster OX=7227 GN=CCHa2 PE=1 SV=1

MKSTISLLLVVICTVVLAAQQSQAKKGCQAYGHVCYGGHGKRSLSPGSGSGTGVGGGMGE

AASGGQEPDYVRPNGLLPMMAPNEQVPLEGDFNDYPARQVLYKIMKSWFNRPRRPASRLG

ELDYPLANSAELNGVN

>sp|P01257|CALC_RAT Calcitonin OS=Rattus norvegicus OX=10116 GN=Calca PE=1 SV=1

MGFLKFSPFLVVSILLLYQACGLQAVPLRSTLESSPGMATLSEEEARLLAALVQNYMQMK

VRELEQEEEQEAEGSSLDSPRSKRCGNLSTCMLGTYTQDLNKFHTFPQTSIGVGAPGKKR

DMAKDLETNHHPYFGN

>sp|P83859|OX26_HUMAN Orexigenic neuropeptide QRFP OS=Homo sapiens OX=9606 GN=QRFP PE=2 SV=1

MVRPYPLIYFLFLPLGACFPLLDRREPTDAMGGLGAGERWADLAMGPRPHSVWGSSRWLR

ASQPQALLVIARGLQTSGREHAGCRFRFGRQDEGSEATGFLPAAGEKTSGPLGNLAEELN

GYSRKKGGFSFRFGRR

>sp|O77220|CHH_MACLE Crustacean hyperglycemic hormones OS=Macrobrachium lanchesteri OX=82204 GN=CHH PE=2 SV=1

MIRSSVMGPTMFLVVLLLIASHQTSAWSLDGLARIEKLLSTSSSASAASPTRGQALNLKK

RAILDQSCKGIFDRELFKKLDRVCDDCYNLYRKPYVAIDCREGCYQNLVFRQCIQDLQLM

DQLDEYANAVQIVGK

>sp|Q865F0|ASIP_FELCA Agouti-signaling protein OS=Felis catus OX=9685 GN=ASIP PE=3 SV=1

MNILRLLLATLLVCLCLLTAYSHLAPEEKPRDDRNLRSNSSMNMLDLSSVSIVALNKKSK

KISRKEAEKKRSSKKKASMKNVAQPRRPRPPPPAPCVATRDSCKPPAPACCDPCASCQCR

FFRSSCSCRVLNPTC

>sp|Q25589|CHH1_FAXLI Crustacean hyperglycemic hormones A OS=Faxonius limosus OX=28379 GN=CHHA PE=1 SV=1

MVSFRTMWSLVVVVVVASLASSGVQGRSVEGSSRMERLLSSGSSSSEPLSFLSQDQSVSK

RQVFDQACKGIYDRAIFKKLDRVCEDCYNLYRKPYVATTCRQNCYANSVFRQCLDDLLLI

DVLDEYISGVQTVGK

>sp|Q25588|CHH2_FAXLI Crustacean hyperglycemic hormones A* OS=Faxonius limosus OX=28379 GN=CHHA* PE=1 SV=1

MVSFRTMWSVVVVVVVASLASSGVQGRSVEGSSRMERLLSSGSSSSEPLSFLSQDQSVNK

RQVFDQACKGIYDRAIFKKLDRVCEDCYNLYRKPYVATTCRQNCYANSVFRQCLDDLLLI

DVLDEYISGVQTVGK

>sp|P83862|OX26_BOVIN Orexigenic neuropeptide QRFP OS=Bos taurus OX=9913 GN=QRFP PE=2 SV=1

MRSPYSLPYLLFLPLGACFPVLDTEEPVDAVGGTGREMSWMDPARGRPFPWGSPGWPRAP

YPHALLVTAKELRASGKARAGFQLRLGRQDDGSEATGLLLGEAEKVGGLLGTLAEELNGY

SRKKGGFSFRFGRR

>sp|P0DN43|TESS_TERSU Terepressin/terephysin OS=Terebra subulata OX=89435 PE=2 SV=1

MKCSVLPRSRLSWTMCVLLLPLLMLMLEGGVQGCFIRNCPRGGKRAVDSVQPTRQCMSCG

PEGVGQCVGPSICCGLAIGCLMGTSEAEVCQKENESSAPCAVSGRHCGMDNTGNCVADGI

CCVEDACSFNSLCR

>sp|Q863C3|GRP_BOVIN Gastrin-releasing peptide OS=Bos taurus OX=9913 GN=GRP PE=1 SV=3

MRGREVPLVLLALVLCLAPRGWAAPVTAGRGGALAKMYTRGNHWAVGHLMGKKSVAESPQ

LHEEESLKEQLREYAQWEEATRNLLSLLQAKGARGHQMPPWEPLSIHQPAWDSEDVSNFK

DTGPQHEGRNPQLN

>sp|Q25154|CHHB_HOMAM Crustacean hyperglycemic hormones isoform B OS=Homarus americanus OX=6706 PE=1 SV=2

MFACRTLCLVVVMVASLGTSGVGGRSVEGVSRMEKLLSSSISPSSTPLGFLSQDHSVNKR

QVFDQACKGVYDRNLFKKLNRVCEDCYNLYRKPFIVTTCRENCYSNRVFRQCLDDLLLSD

VIDEYVSNVQMVGK

>sp|Q26492|ITPL_SCHGR Ion transport peptide-like OS=Schistocerca gregaria OX=7010 PE=2 SV=1

MHHQKQQQQQKQQGEAPCRHLQWRLSGVVLCVLVVASLVSTAASSPLDPHHLAKRSFFDI

QCKGVYDKSIFARLDRICEDCYNLFREPQLHSLCRKDCFTSDYFKGCIDVLLLQDDMDKI

QSWIKQIHGAEPGV

>sp|P19806|CHHA_HOMAM Crustacean hyperglycemic hormones isoform A OS=Homarus americanus OX=6706 PE=1 SV=3

MMACRTLCLVVVMVASLGTSGVGGRSVEGASRMEKLLSSSNSPSSTPLGFLSQDHSVNKR

QVFDQACKGVYDRNLFKKLDRVCEDCYNLYRKPFVATTCRENCYSNWVFRQCLDDLLLSD

VIDEYVSNVQMVGK

>sp|P56413|AGRP_BOVIN Agouti-related protein OS=Bos taurus OX=9913 GN=AGRP PE=2 SV=1

MLTAVLLSCALLLAMPPLQGAQMGPAPLEGIGRPEEALFLELQGLSLQPSLKRITEEQAE

ESLLQEAEAKALAEVLDPEGRKPRSPRRCVRLHESCLGHQVPCCDPCATCYCRFFNAFCY

CRKLGTTTNPCSRT

>sp|Q9TU18|AGRP_PIG Agouti-related protein OS=Sus scrofa OX=9823 GN=AGRP PE=3 SV=2

MLTTMLLSCALLLAMPTMLGAQIGLAPLEGIGRLDQALFPELQDLGLQPPLKRTTAERAE

EALLQQAEAKALAEVLDPEGRKARSPRRCVRLHESCLGHQVPCCDPCATCYCRFFNAFCY

CRKLGTATNPCSRT

>sp|P0DN42|TESS_TERAN Terepressin/terephysin OS=Terebra anilis OX=553697 PE=2 SV=1

MKCSVLQMSRLSWTACVLLLPLLLLTLQGGVQGCFIRNCPRGGKRAVDSVQPTRQCMSCG

PEGVGQCVGPSICCGLAIGCLMGTPEAEVCQKENESSAPCAVSGRHCGMDNTGNCVADGI

CCVEDACSFNSLCR

>sp|P47851|GRP_SHEEP Gastrin-releasing peptide OS=Ovis aries OX=9940 GN=GRP PE=2 SV=1

MRSREVSLVLLALVLCPAPRGSAAPVTAGRAGALAKMYTRGNHWAVGHLMGKKSVAESPQ

LREEESLKEQLREYAQWEEATRNLLSLLQAKVAQGHQPPRWEPLSIHQPAWDSKDVSNFK

DSGSQREGGNPQLY

>sp|Q95MP2|ASIP_HORSE Agouti-signaling protein OS=Equus caballus OX=9796 GN=ASIP PE=3 SV=1

MDVIHLFLATLLVSLCFLTAYSHLSPEEKPKDDRSLRNNSSMNLLDSPSVSIMALNKKSK

KISRKEAEKKKRSSKKKASMTKVARPRLLQPAPCVATRDSCKPPAPACCDPCASCQCRFF

RSACSCRVLTRTC

>sp|Q29414|ASIP_BOVIN Agouti-signaling protein OS=Bos taurus OX=9913 GN=ASIP PE=2 SV=2

MDVSRLLLATLLVCLCFLTAYSHLAPEEKPRDERNLKNNSSMNLLDFPSVSIVALNKKSK

KISRNEAEKKKRPSKRKAPMKNVARTRPPPPTPCVATRDSCKPPAPACCDPCAFCQCRFF

RSACSCRVLNPTC

>sp|P21916|NEU2_STRCA Neurophysin 2 OS=Struthio camelus OX=8801 PE=1 SV=1

ALADAALRQCMPCGPGDRGNCFGPSICCGAELGCYVGTAETLRCAEENYLPSPCRAGGQP

CGAGGRCAAPGICCSDETCSLEPACLEEAGERGGEPAQKNLTGLDASAGDFLLKLMHLAA

NRQQQGGKGPLL

>sp|A1YL67|ASIP_MACMU Agouti-signaling protein OS=Macaca mulatta OX=9544 GN=ASIP PE=3 SV=1

MDVTRLLLATLLVFLCFFTAYSHLPPEEKLRDDRSLRSNSSVNLLDFPSVSIMALNKNSK

EISRKEAEKKRSSKKEASMKKVARPRTPLSAPCVATRDSCKPPAPACCDPCASCQCRFFR

SACSCRVLSLNC

>sp|P42127|ASIP_HUMAN Agouti-signaling protein OS=Homo sapiens OX=9606 GN=ASIP PE=1 SV=1

MDVTRLLLATLLVFLCFFTANSHLPPEEKLRDDRSLRSNSSVNLLDVPSVSIVALNKKSK

QIGRKAAEKKRSSKKEASMKKVVRPRTPLSAPCVATRNSCKPPAPACCDPCASCQCRFFR

SACSCRVLSLNC

>sp|Q1XGV5|ASIP_GORGO Agouti-signaling protein OS=Gorilla gorilla gorilla OX=9595 GN=ASIP PE=3 SV=1

MDVTRLLLATLLVFLCFFTANSHLPPEEKLRDDRSLRSNSSVNLLDFPSVSIVALNKKSK

QIGRKEAEKKRSSKKEASMKKVARPRTPLSAPCVATRNSCKPPAPACCDPCASCQCRFFR

SACSCRVLSLNC

>sp|Q1XGV7|ASIP_PANTR Agouti-signaling protein OS=Pan troglodytes OX=9598 GN=ASIP PE=3 SV=1

MDVTRLLLATLLVFLCFFTANSHLPPEEKLRDDRSLRSNSSVNLLDFPSVSIVALNKKSK

QIGRKEAEKKRSSKKEASMKKVARPRTPLSAPCVATRNSCKPPAPACCDPCASCQCRFFR

SACSCRVLSLNC

>sp|O00253|AGRP_HUMAN Agouti-related protein OS=Homo sapiens OX=9606 GN=AGRP PE=1 SV=1

MLTAAVLSCALLLALPATRGAQMGLAPMEGIRRPDQALLPELPGLGLRAPLKKTTAEQAE

EDLLQEAQALAEVLDLQDREPRSSRRCVRLHESCLGQQVPCCDPCATCYCRFFNAFCYCR

KLGTAMNPCSRT

>sp|O43612|OREX_HUMAN Orexin OS=Homo sapiens OX=9606 GN=HCRT PE=1 SV=1

MNLPSTKVSWAAVTLLLLLLLLPPALLSSGAAAQPLPDCCRQKTCSCRLYELLHGAGNHA

AGILTLGKRRSGPPGLQGRLQRLLQASGNHAAGILTMGRRAGAEPAPRPCLGRRCSAPAA

ASVAPGGQSGI

>sp|Q5UK76|ASIP_CANLF Agouti-signaling protein OS=Canis lupus familiaris OX=9615 GN=ASIP PE=2 SV=1

MNIFRLLLATLLVSLCFLTAYSHLAEEKPKDDRSLRSNSSVNLLDFPSVSIVALNKKSKK

ISRKEAEKKRSSKKKASMKNVARPRPPPPTPCVATRNSCKSPAPACCDPCASCQCRFFRS

ACTCRVLSPRC

>sp|Q6ZYM3|ASIP_PIG Agouti-signaling protein OS=Sus scrofa OX=9823 GN=ASIP PE=2 SV=1

MDVTRLLLATLLVCLCFFTASSHLAPEEKSKDERSLRSNSSMNLLDFPSVSIVALNKKSK

KISRKEAEKRSSKKKASMKKVAQPRPPRPAPCVANRDSCKPPALACCDPCAFCQCRFFRS

ACSCRVLNPTC

>sp|P56473|AGRP_MOUSE Agouti-related protein OS=Mus musculus OX=10090 GN=Agrp PE=1 SV=1

MLTAMLLSCVLLLALPPTLGVQMGVAPLKGIRRPDQALFPEFPGLSLNGLKKTTADRAEE

VLLQKAEALAEVLDPQNRESRSPRRCVRLHESCLGQQVPCCDPCATCYCRFFNAFCYCRK

LGTATNLCSRT

>sp|Q99JA2|ASIP_RAT Agouti-signaling protein OS=Rattus norvegicus OX=10116 GN=Asip PE=2 SV=1

MDVTRLLLATLVGFLCFLTVHSHLVFEETLGDDRSLKSNSSINSLDFSSVSIVALNKKSK

KISRKEAEKRKRSSKKKASIKKVARPPPPSPCVATRDSCKPPAPACCNPCASCQCRFFGS

ACTCRVLNPNC

>sp|Q03288|ASIP_MOUSE Agouti-signaling protein OS=Mus musculus OX=10090 GN=Asip PE=2 SV=2

MDVTRLLLATLVGFLCFFTVHSHLALEETLGDDRSLRSNSSMNSLDFSSVSIVALNKKSK

KISRKEAEKRKRSSKKKASMKKVARPPPPSPCVATRDSCKPPAPACCDPCASCQCRFFGS

ACTCRVLNPNC

>sp|P01302|PAHO_BOVIN Pancreatic prohormone OS=Bos taurus OX=9913 GN=PPY PE=1 SV=2

MAAAHRCLFLLLLSTCVALLLQPPLGALGAPLEPEYPGDNATPEQMAQYAAELRRYINML

TRPRYGKRDKEGTLDFLECGSPHSAVPRYGKRDKEGTLDFLECGSPHSAVPRWVFSLSCV

PRCLGQENGGV

>sp|P19630|NEU2_ANSAN Neurophysin 2 OS=Anser anser anser OX=8844 PE=1 SV=1

ALADTALRQCLPCGPGNRGRCFGPGICCGVELGGCYVGTAETRRCAEEDYLPSPCQPGGQ

PCGSGGRCAADGVCCSADTCAADASCLEEGSERAEEAAEKNLTVLDGSAGDLLLRLMQLA

GRQQGRQPGLL

>sp|O77668|OREX_PIG Orexin OS=Sus scrofa OX=9823 GN=HCRT PE=2 SV=1

MNPPFAKVSWATVTLLLLLLLLPPAVLSPGAAAQPLPDCCRQKTCSCRLYELLHGAGNHA

AGILTLGKRRPGPPGLQGRLQRLLQASGNHAAGILTMGRRAGAEPAPRLCPGRRCLAAAA

SSVAPGGRSGI

>sp|O55232|OREX_RAT Orexin OS=Rattus norvegicus OX=10116 GN=Hcrt PE=1 SV=1

MNLPSTKVPWAAVTLLLLLLLPPALLSLGVDAQPLPDCCRQKTCSCRLYELLHGAGNHAA

GILTLGKRRPGPPGLQGRLQRLLQANGNHAAGILTMGRRAGAELEPYPCPGRRCPTATAT

ALAPRGGSRV

>sp|Q26491|ITP_SCHGR Ion transport peptide OS=Schistocerca gregaria OX=7010 PE=2 SV=1

MHHQKQQQQQKQQGEAPCRHLQWRLSGVVLCVLVVASLVSTAASSPLDPHHLAKRSFFDI

QCKGVYDKSIFARLDRICEDCYNLFREPQLHSLCRSDCFKSPYFKGCLQALLLIDEEEKF

NQMVEILGKK

>sp|Q9GLF6|OREX_CANLF Orexin OS=Canis lupus familiaris OX=9615 GN=HCRT PE=3 SV=1

MNPPSTKVPWAAVTLLLLLLLPPALLSPGAAAQPLPDCCRQKTCSCRLYELLHGAGNHAA

GILTLGKRRPGPPGLQGRLQRLLQASGNHAAGILTMGRRAGAEPAPRPCPGRRCPVVAVP

SAAPGGRSGV

>sp|Q9PU41|CCKN_CHICK Cholecystokinin OS=Gallus gallus OX=9031 GN=CCK PE=1 SV=1

MYGGICICVLLAALSVSSLGQQPAGSHDGSPVAAELQQSLTEPHRHSRAPSSAGPLKPAP

RLDGSFEQRATIGALLAKYLQQARKGSTGRFSVLGNRVQSIDPTHRINDRDYMGWMDFGR

RSAEEYEYSS

>sp|O55241|OREX_MOUSE Orexin OS=Mus musculus OX=10090 GN=Hcrt PE=2 SV=1

MNFPSTKVPWAAVTLLLLLLLPPALLSLGVDAQPLPDCCRQKTCSCRLYELLHGAGNHAA

GILTLGKRRPGPPGLQGRLQRLLQANGNHAAGILTMGRRAGAELEPHPCSGRGCPTVTTT

ALAPRGGSGV

>sp|P49192|CART_RAT Cocaine- and amphetamine-regulated transcript protein OS=Rattus norvegicus OX=10116 GN=Cartpt PE=1 SV=1

MESSRLRLLPVLGAALLLLLPLLGAGAQEDAELQPRALDIYSAVDDASHEKELPRRQLRA

PGAVLQIEALQEVLKKLKSKRIPIYEKKYGQVPMCDAGEQCAVRKGARIGKLCDCPRGTS

CNSFLLKCL

>sp|P56388|CART_MOUSE Cocaine- and amphetamine-regulated transcript protein OS=Mus musculus OX=10090 GN=Cartpt PE=1 SV=2

MESSRLRLLPLLGAALLLLLPLLGARAQEDAELQPRALDIYSAVDDASHEKELPRRQLRA

PGAMLQIEALQEVLKKLKSKRIPIYEKKYGQVPMCDAGEQCAVRKGARIGKLCDCPRGTS

CNSFLLKCL

>sp|Q9GQV7|HPEP_AEDAE Head peptide OS=Aedes aegypti OX=7159 GN=HP-I PE=1 SV=1

MWKFASIVVLVVCLAWAVYCEDQRPPSLKTRFGRSADEPESDNYVSNDIMEKRSAQRPPS

LKTRFGRSEGAEVMEKRSAQRPPSLKTRFGRSVANPESDGYMRKRSAESEPFVTRIRHGR

ANKKRAAN

>sp|P05486|CESS_CONGE Conophysin-conopressin OS=Conus geographus OX=6491 PE=1 SV=2

MTRSAMQMGRLTLVLCLLLLLLLTTQACFIRNCPKGGKRDVDERYLFKACMSCSFGQCVG

PRICCGPRGCEMGTAEANRCIEEDEDPIPCQVVGQHCDLNNPGNIHGNCVANGICCVDDT

CTIHTGCL

>sp|P01256|CALCA_RAT Calcitonin gene-related peptide 1 OS=Rattus norvegicus OX=10116 GN=Calca PE=2 SV=1

MGFLKFSPFLVVSILLLYQACGLQAVPLRSTLESSPGMAATLSEEEARLLLAALVQNYMQ

MKVRELEQEQEAEGSSVTAQKRSCNTATCVTHRLAGLLSRSGGVVKDNFVPTNVGSEAFG

RRRRDLQA

>sp|P10092|CALCB_HUMAN Calcitonin gene-related peptide 2 OS=Homo sapiens OX=9606 GN=CALCB PE=1 SV=1

MGFRKFSPFLALSILVLYQAGSLQAAPFRSALESSPDPATLSKEDARLLLAALVQDYVQM

KASELKQEQETQGSSSAAQKRACNTATCVTHRLAGLLSRSGGMVKSNFVPTNVGSKAFGR

RRRDLQA

>sp|Q61839|ANFC_MOUSE C-type natriuretic peptide OS=Mus musculus OX=10090 GN=Nppc PE=3 SV=1

MHLSQLIACALLLALLSLRPSEAKPGTPPKVPRTPPGEELADSQAAGGNQKKGDKTPGSG

GANLKGDRSRLLRDLRVDTKSRAAWARLLHEHPNARKYKGGNKKGLSKGCFGLKLDRIGS

MSGLGC

>sp|P13389|NEU1_SHEEP Oxytocin-neurophysin 1 OS=Ovis aries OX=9940 GN=OXT PE=1 SV=2

MAGSSLACCLLGLLALTSACYIQNCPLGGKRAVLDLDVRTCLPCGPGGKGRCFGPSICCG

DELGCFVGTAEALRCREENYLPSPCQSGQKPCGSGGRCAAAGICCSPDGCHADPACDPEA

AFSQH

>sp|P01175|NEU1_BOVIN Oxytocin-neurophysin 1 OS=Bos taurus OX=9913 GN=OXT PE=1 SV=1

MAGSSLACCLLGLLALTSACYIQNCPLGGKRAVLDLDVRTCLPCGPGGKGRCFGPSICCG

DELGCFVGTAEALRCQEENYLPSPCQSGQKPCGSGGRCAAAGICCSPDGCHEDPACDPEA

AFSQH

>sp|P01178|NEU1_HUMAN Oxytocin-neurophysin 1 OS=Homo sapiens OX=9606 GN=OXT PE=1 SV=1

MAGPSLACCLLGLLALTSACYIQNCPLGGKRAAPDLDVRKCLPCGPGGKGRCFGPNICCA

EELGCFVGTAEALRCQEENYLPSPCQSGQKACGSGGRCAVLGLCCSPDGCHADPACDAEA

TFSQR

>sp|Q8WRC7|CCAP_MANSE Cardioactive peptide OS=Manduca sexta OX=7130 GN=CCAP PE=1 SV=1

MTVSRVCLLLLVALVYLDCCYAASIPRNFDPRLSEEIVMAPKKRPFCNAFTGCGRKRSQG

PPGMPAQDLRTKQYLDEEALGSILDSESAIDELSRQILSEAKLWEAIQEASAEIARRKQK

EAYIQ

>sp|A0A291NVT7|CESS1_CONMO Conophysin-conopressin, isoform 1 (Fragment) OS=Conus monile OX=351660 PE=1 SV=1

MQMGRPTLLPCLLLLLVLSTQACFIRNCPEGGKRDVHMIQPTKPCMNCSFGQCVGPRVCC

GAGRCEIGSTEADRCEEENEDPVPCKVLGQHCVLNNPGNVNGNCVDGGIGICCVDDTCAI

HRRCD

>sp|O15981|CHH5_PENJP Crustacean hyperglycemic hormones 5 OS=Penaeus japonicus OX=27405 PE=1 SV=1

MKPGNTSFNMVSFRMVWTAMMATLLVAGASSAGTRSSEDLSAPEDRSLSKRLVFDPSCAG

VYDRVLLGKLNRLCDDCYNVFREPNVATECRSNCFYNLAFVQCLEYLMPPSLHEEYQANV

QMVGK

>sp|Q868F8|ALLS_SPOFR Allatostatin OS=Spodoptera frugiperda OX=7108 GN=ast PE=2 SV=1

MKTSAYNVYLGVVAAMLALLFVTINAAPMEADDETAENTLVAHPDGDMELSGPWDAINTA

ALRKLLLQLDAEDRMGGVTRSWPQAEPRGWGLRALDSRLARQWRADKRQVRFRQCYFNPI

SCFRK

>sp|P01179|NEU1_RAT Oxytocin-neurophysin 1 OS=Rattus norvegicus OX=10116 GN=Oxt PE=1 SV=2

MACPSLACCLLGLLALTSACYIQNCPLGGKRAALDLDMRKCLPCGPGGKGRCFGPSICCA

DELGCFVGTAEALRCQEENYLPSPCQSGQKPCGSGGRCATAGICCSPDGCRTDPACDPES

AFSER

>sp|P01177|NEU1_PIG Oxytocin-neurophysin 1 OS=Sus scrofa OX=9823 GN=OXT PE=1 SV=3

MAGPSLACCLLGLLALTSACYIQNCPLGGKRAVLDLDVRKCLPCGPGGKGRCFGPSICCG

DELGCFVGTAEALRCQEENYLPSPCQSGQKPCGSEGRCAAAGICCNPDGCRFDPACDPEA

TFSQR

>sp|P08162|NEUM_BUFJA Mesotocin-neurophysin MT OS=Bufo japonicus OX=8387 PE=2 SV=1

MSYTALAVTFFGWLALSSACYIQNCPIGGKRSVIDFMDVRKCIPCGPRNKGHCFGPNICC

GEELGCYFGTTETLRCQEENFLPSPCESGRKPCGNNGGNCARSGICCNHESCTMDPACEQ

DSVFS

>sp|P35454|NEU1_MOUSE Oxytocin-neurophysin 1 OS=Mus musculus OX=10090 GN=Oxt PE=2 SV=1

MACPSLACCLLGLLALTSACYIQNCPLGGKRAVLDLDMRKCLPCGPGGKGRCFGPSICCA

DELGCFVGTAEALRCQEENYLPSPCQSGQKPCGSGGRCAATGICCSPDGCRTDPACDPES

AFSER

>sp|Q8CE23|OX26_MOUSE Orexigenic neuropeptide QRFP OS=Mus musculus OX=10090 GN=Qrfp PE=2 SV=1

MRGFRPLLSLLLPLSACFPLLDRRGPTDIGDIGARMNWAQLAEGHPPNSVQNPQPQALLV

VAREQQASHREHTGFRLGRQDGSSEAAGFLPADSEKASGPLGTLAEELSSYSRRKGGFSF

RFGR

>sp|P47212|GALA_MOUSE Galanin peptides OS=Mus musculus OX=10090 GN=Gal PE=2 SV=1

MARGSVILLGWLLLVVTLSATLGLGMPAKEKRGWTLNSAGYLLGPHAIDNHRSFSDKHGL

TGKRELQLEVEERRPGSVDVPLPESNIVRTIMEFLSFLHLKEAGALDSLPGIPLATSSED

LEKS

>sp|P10683|GALA_RAT Galanin peptides OS=Rattus norvegicus OX=10116 GN=Gal PE=1 SV=1

MARGSVILLAWLLLVATLSATLGLGMPTKEKRGWTLNSAGYLLGPHAIDNHRSFSDKHGL

TGKRELPLEVEEGRLGSVAVPLPESNIVRTIMEFLSFLHLKEAGALDSLPGIPLATSSED

LEQS

>sp|P83860|OX26_RAT Orexigenic neuropeptide QRFP OS=Rattus norvegicus OX=10116 GN=Qrfp PE=1 SV=1

MRCLCSWLCLLLPLSACFPLLDRRGPTDIGDIGARMSWVQLTEGHTPRSVQSPRPQALLV

VAKEQQASRREHTGFRLGRQDSGSEATGFLPTDSEKASGPLGTLAEELSSYSRRKGGFSF

RFGR

>sp|P55089|UCN1_HUMAN Urocortin OS=Homo sapiens OX=9606 GN=UCN PE=1 SV=1

MRQAGRAALLAALLLLVQLCPGSSQRSPEAAGVQDPSLRWSPGARNQGGGARALLLLLAE

RFPRRAGPGRLGLGTAGERPRRDNPSLSIDLTFHLLRTLLELARTQSQRERAEQNRIIFD

SVGK

>sp|P11242|GALA_BOVIN Galanin peptides OS=Bos taurus OX=9913 GN=GAL PE=1 SV=1

MPRGSVLLLASLLLAAALSATLGLGSPVKEKRGWTLNSAGYLLGPHALDSHRSFQDKHGL

AGKRELEPEDEARPGSFDRPLAENNVVRTIIEFLTFLHLKDAGALERLPSLPTAESAEDA

ERS

>sp|P22466|GALA_HUMAN Galanin peptides OS=Homo sapiens OX=9606 GN=GAL PE=1 SV=3

MARGSALLLASLLLAAALSASAGLWSPAKEKRGWTLNSAGYLLGPHAVGNHRSFSDKNGL

TSKRELRPEDDMKPGSFDRSIPENNIMRTIIEFLSFLHLKEAGALDRLLDLPAAASSEDI

ERS

>sp|P07480|GALA_PIG Galanin peptides OS=Sus scrofa OX=9823 GN=GAL PE=1 SV=1

MPRGCALLLASLLLASALSATLGLGSPVKEKRGWTLNSAGYLLGPHAIDNHRSFHDKYGL

AGKRELEPEDEARPGGFDRLQSEDKAIRTIMEFLAFLHLKEAGALGRLPGLPSAASSEDA

GQS

>sp|O15982|CHH7_PENJP Crustacean hyperglycemic hormones 7 OS=Penaeus japonicus OX=27405 PE=2 SV=1

MSLAMTAFRMMAVALVVVVASSTTWARSLEGSSSPVTSLTRGRSLNKRAAFDPSCTGVYD

RELLGRLSRLCDDCYNVFREPKVAMECRSNCFFNPAFVQCLEYLIPAELHEEYQALVQTV

GK

>sp|O97387|CHH5_PENMO Crustacean hyperglycemic hormones 5 OS=Penaeus monodon OX=6687 GN=CHH5 PE=2 SV=1

MSLGLIASRLVAVALVVVVACSTTWARSLEGSSSPVASLIRGRSLSKRANFDPSCAGVYD

RELLGGLSRLCDDCYNVFREPKVATECRSNCFYNSVFVQCLEYLIPADLHEEYQAHVQTV

GK

>sp|Q2T9U8|NMB_BOVIN Neuromedin-B OS=Bos taurus OX=9913 GN=NMB PE=2 SV=1

MTLRAVGVRLLGGLLLFALLAAGAAPLGWDLPESRSRASKIRVHPRGNLWATGHFMGKKS

LEPPSPSLLGTAPHTSLRDQTPQLSHHLLRVLLQKQALGMSLSVPAPNTQHRRLLVQTLQ

K

>sp|Q9CR53|NMB_MOUSE Neuromedin-B OS=Mus musculus OX=10090 GN=Nmb PE=2 SV=1

MTRQAGSSWLLRGLLLFALFASGVAPFNWDLPEPRSRASKIRVHPRGNLWATGHFMGKKS

LEPPSLSLVGTAPPNTPRDQRLQLSHDLLRILLRKKALGMNFSGPAPPIQYRRLLEPLLQ

K

>sp|P08949|NMB_HUMAN Neuromedin-B OS=Homo sapiens OX=9606 GN=NMB PE=1 SV=4

MARRAGGARMFGSLLLFALLAAGVAPLSWDLPEPRSRASKIRVHSRGNLWATGHFMGKKS

LEPSSPSPLGTAPHTSLRDQRLQLSHDLLGILLLKKALGVSLSRPAPQIQYRRLLVQILQ

K

>sp|O97386|CHH4_PENMO Crustacean hyperglycemic hormones 4 OS=Penaeus monodon OX=6687 GN=CHH4 PE=2 SV=1

MVALNTLSAVSAALLVLAASPSPASARSLDASPSSAFSGNHSLSKRSLFDPACTGIYDRQ

LLGKLGRLCDDCYNVFREPKVATGCRSNCYYNLIFLDCLEYLIPSHLQEEHMEALQTVGK

>sp|Q9U5D2|CHH2_PENJP Crustacean hyperglycemic hormones 2 OS=Penaeus japonicus OX=27405 PE=1 SV=1

MIAFHMVWSALLASLLLLLLAPSASPVDAFSPPEASLTGGQSLSKRSLFDPSCTGVFDRQ

LLRRLGRVCDDCFNVFREPNVAMECRSNCYNNPVFRQCMEYLLPAHLHDEYRLAVQMVGK

>sp|O97383|CHH1_PENMO Crustacean hyperglycemic hormones 1 OS=Penaeus monodon OX=6687 GN=CHH1 PE=2 SV=1

MTAFRMVWSMLLASLLMLLVASSTAPADALSPPAAGLGADHSFTKRSLFDPSCTGVFDRQ

LLRRLSRVCDDCFNVFREPNVATECRSNCYNNEVFRQCMEYLLPAHLHEEHRLAVQMVGK

>sp|O15980|CHH1_PENJP Crustacean hyperglycemic hormones 1 OS=Penaeus japonicus OX=27405 PE=1 SV=1

MIAFRAVWSALLASLLLLLLAPSASPVDAFSPPEASLTGGQSLSKRSLFDPSCTGVFDRQ

LLRRLGRVCDDCFNVFREPNVATECRSNCYNNPVFRQCMAYVVPAHLHNEHREAVQMVGK

>sp|O97384|CHH2_PENMO Crustacean hyperglycemic hormones 2 OS=Penaeus monodon OX=6687 GN=CHH2 PE=3 SV=1

MTAFRLVAVALVVVVACSTTWARSLEGSSSPVASLIRGRSLSKRANFDPSCAGVYNRELL

GRLSRLCDDCYNVFREPKVATECRSNCFYNPVFVQCLEYLIPADLHEEYQAHVQTVGK

>sp|E7EZ53|GALA_DANRE Galanin peptides OS=Danio rerio OX=7955 GN=gal PE=1 SV=1

MHRCVGGVCVSLIVCAFLTETLGMVIAAKEKRGWTLNSAGYLLGPHAIDSHRSLSDKHGL

AGKREMPLDEDFKTGALRIADEDVVHTIIDFLSYLKLKEIGALDSLPSSLTSEEISQP

>sp|Q9W6M9|GALA_COTJA Galanin peptides OS=Coturnix japonica OX=93934 GN=GAL PE=3 SV=2

MQRCAGFLFLSLILCAALSETFGLVLSAKEKRGWTLNSAGYLLGPHAVDNHRSFNDKHGF

TGKREIQPEEDIKAGNIGRPLADENIVRTVVEFLTYLHLKEAGALDNLPSPEETNES

>sp|Q94676|CHH3_PENJP Crustacean hyperglycemic hormones 3 OS=Penaeus japonicus OX=27405 PE=2 SV=1

MVTPRMLSALSAVLLLVLTASSSARSFDASPSATSGNHSLNKRSLFDPACTGIYDRQLLR

KLGRLCDDCYNVFREPKVATGCRSNCYHNLIFLDCLEYLIPSHLQEEHMAAMQTVGK

>sp|Q68RJ9|CART_BOVIN Cocaine- and amphetamine-regulated transcript protein OS=Bos taurus OX=9913 GN=CARTPT PE=3 SV=1

MESPRLRLLPLLGAALLLLLPLLGALAQEDAELQPRALDIYSAVEDASHEKELIEALQEV

LKKLKSKRIPIYEKKYGQVPMCDAGEQCAVRKGARIGKLCDCPRGTSCNSFLLKCL

>sp|Q16568|CART_HUMAN Cocaine- and amphetamine-regulated transcript protein OS=Homo sapiens OX=9606 GN=CARTPT PE=1 SV=1

MESSRVRLLPLLGAALLLMLPLLGTRAQEDAELQPRALDIYSAVDDASHEKELIEALQEV

LKKLKSKRVPIYEKKYGQVPMCDAGEQCAVRKGARIGKLCDCPRGTSCNSFLLKCL

>sp|Q9VLK4|DIUX_DROME Diuretic hormone class 2 OS=Drosophila melanogaster OX=7227 GN=Dh31 PE=1 SV=1

MTNRCACFALAFLLFCLLAISSIEAAPMPSQSNGGYGGAGYNELEEVPDDLLMELMTRFG

RTIIRARNDLENSKRTVDFGLARGYSGTQEAKHRMGLAAANFAGGPGRRRRSETDV

>sp|M0R8L2|SPXN_RAT Spexin OS=Rattus norvegicus OX=10116 GN=SPX PE=1 SV=1

MKGPSILAVAALALLLVLSVLENSSGAPQRLSEKRNWTPQAMLYLKGAQGHRFISDQSRR

KELADRPPPERRNPNLQLLTLPEAAALFLASLEKPQKDEGGDFDKSKLLEDRRFYW

>sp|D3Z752|SPXN_MOUSE Spexin OS=Mus musculus OX=10090 GN=Spx PE=2 SV=1

MKGPSVLAVTAVVLLLVLSALENSSGAPQRLSEKRNWTPQAMLYLKGAQGRRFLSDQSRR

KELADRPPPERRNPDLELLTLPEAAALFLASLEKSQKDEGGNFDKSELLEDRLFNW

>sp|Q9BT56|SPXN_HUMAN Spexin OS=Homo sapiens OX=9606 GN=SPX PE=1 SV=1

MKGLRSLAATTLALFLVFVFLGNSSCAPQRLLERRNWTPQAMLYLKGAQGRRFISDQSRR

KDLSDRPLPERRSPNPQLLTIPEAATILLASLQKSPEDEEKNFDQTRFLEDSLLNW

>sp|Q0VC44|SPXN_BOVIN Spexin OS=Bos taurus OX=9913 GN=SPX PE=3 SV=1

MKGFKSLVVMTLTLFLVFSFMGNCNSAPQRLFERRNWTPQAMLYLKGAQGRRFLSDQSRR

KDLSDRPPLERRSPNSQQLTLPEAAAVLLAFLQKPQEAGDENLDQTRFLEDSLLNW

>sp|Q9TUX7|NPFF_BOVIN Pro-FMRFamide-related neuropeptide FF OS=Bos taurus OX=9913 GN=NPFF PE=3 SV=1

MDARQAAALLLVLLLVTDWSHAEGPGGRDGGDQIFMEEDSGAHPAQDAQTPRSLLRSLLQ

AMQRPGRSPAFLFQPQRFGRNTRGSWSNKRLSPRAGEGLSSPFWSLAAPQRFGKK

>sp|P01355|CCKN_RAT Cholecystokinin OS=Rattus norvegicus OX=10116 GN=Cck PE=1 SV=1

MKCGVCLCVVMAVLAAGALAQPVVPVEAVDPMEQRAEEAPRRQLRAVLRPDSEPRARLGA

LLARYIQQVRKAPSGRMSVLKNLQGLDPSHRISDRDYMGWMDFGRRSAEDYEYPS

>sp|P06307|CCKN_HUMAN Cholecystokinin OS=Homo sapiens OX=9606 GN=CCK PE=1 SV=1

MNSGVCLCVLMAVLAAGALTQPVPPADPAGSGLQRAEEAPRRQLRVSQRTDGESRAHLGA

LLARYIQQARKAPSGRMSIVKNLQNLDPSHRISDRDYMGWMDFGRRSAEEYEYPS

>sp|P09240|CCKN_MOUSE Cholecystokinin OS=Mus musculus OX=10090 GN=Cck PE=1 SV=3

MKSGVCLCVVMAVLAAGALAQPVVPAEATDPVEQRAQEAPRRQLRAVLRTDGEPRARLGA

LLARYIQQVRKAPSGRMSVLKNLQSLDPSHRISDRDYMGWMDFGRRSAEDYEYPS

>sp|P41520|CCKN_BOVIN Cholecystokinin OS=Bos taurus OX=9913 GN=CCK PE=1 SV=2

MNRGVCLCLLMAVLAAGALAQPMPHADPTGPRAQQAEEAPRRQLRAVPRVDDEPRAQLGA

LLARYIQQARKAPSGRMSVIKNLQSLDPSHRISDRDYMGWMDFGRRSAEEFEYTS

>sp|P23362|CCKN_MACFA Cholecystokinin OS=Macaca fascicularis OX=9541 GN=CCK PE=3 SV=1

MNSGVSLCVLMAVLAAGALTQPVPPAEPAGSGLQRAEEAPRRQLRAVQRTDGESRAHLGA

LLARYIQQARKAPSGRMSIIKNLQNLDPSHRISDRDYMGWMDFGRRSAEEYEYPS

>sp|P01356|CCKN_PIG Cholecystokinin OS=Sus scrofa OX=9823 GN=CCK PE=1 SV=1

MNGGLCLCVLMAVLAAGTLAQPVPPADSAVPGAQEEEAHRRQLRAVQKVDGESRAHLGAL

LARYIQQARKAPSGRVSMIKNLQSLDPSHRISDRDYMGWMDFGRRSAEEYEYTS

>sp|Q9WVA9|NPFF_RAT Pro-FMRFamide-related neuropeptide FF OS=Rattus norvegicus OX=10116 GN=Npff PE=3 SV=1

MDSKWAAVLLLLLLLRNWGHAEEAGSWGEDQVFAEEDKGPHPSQYAHTPDRIQTPGSLMR

VLLQAMERPRRNPAFLFQPQRFGRNAWGPWSKEQLSPQAREFWSLAAPQRFGKK

>sp|Q9WVA8|NPFF_MOUSE Pro-FMRFamide-related neuropeptide FF OS=Mus musculus OX=10090 GN=Npff PE=3 SV=1

MDSKWAALLLLLLLLLNWGHTEEAGSWGEDQVFAGEDKGPHPPQYAHIPDRIQTPGSLFR

VLLQAMDTPRRSPAFLFQPQRFGRSAWGSWSKEQLNPQARQFWSLAAPQRFGKK

>sp|O96605|MIH_CHAFE Molt-inhibiting hormone OS=Charybdis feriata OX=65693 GN=MIH PE=3 SV=1

MMSRANSRFSCQRTWLLAVVVLAAIWSSSLHQAAARVFNDDCPNLMGNRDLYKKVEWICD

DCANIFRIPGMASICRKDCFFNEDFLWCVRATERTEEMMQLKQWVRILGAGRM

>sp|O61389|MIH_METMG Molt-inhibiting hormone OS=Metacarcinus magister OX=29965 PE=3 SV=1

MMSRTESRYSSQRTWLLSMVVLAALWSISVQRATARVINDDCPNLIGNRDLYKRVEWICE

DCSNIFRNTGMATLCRKNCFFNEDFLWCVYATERTEEMSQLRQWVGILGAGRE

>sp|O15130|NPFF_HUMAN Pro-FMRFamide-related neuropeptide FF OS=Homo sapiens OX=9606 GN=NPFF PE=1 SV=1

MDSRQAAALLVLLLLIDGGCAEGPGGQQEDQLSAEEDSEPLPPQDAQTSGSLLHYLLQAM

ERPGRSQAFLFQPQRFGRNTQGSWRNEWLSPRAGEGLNSQFWSLAAPQRFGKK

>sp|Q9GSA4|CORZ_GALME Pro-corazonin OS=Galleria mellonella OX=7137 GN=crz PE=1 SV=1

MATNITMFLIVITLTSVAAQTFQYSRGWTNGKRDGHKTEDIRDLTNNLERILSPCQMNKL

KYVLEGKPLNERLLGPCDTSKTRSTTNPSDTNTSAVKTPCSTHFNKHCYSFSY

>sp|Q9NGP0|CHHB_METEN Crustacean hyperglycemic hormones B OS=Metapenaeus ensis OX=32278 PE=2 SV=1

MVAFRMMSMALLVVVASSWWASPVEAASSPRVDHRLVRRSLFDPSCTGVFDRELLGRLNR

VCDDCYNVFREPKVATECRSHCFLNPAFIQCLEYIIPEVLHEEYQANVQLVGK

>sp|P55321|MIH_CALSI Molt-inhibiting hormone OS=Callinectes sapidus OX=6763 PE=3 SV=1

MMSLAHSKFSCQRTRLLAVVLLAALWSSSLQQAAARVINDDCPNLIGNRDLYKKVEWICD

DCANIYRSTGMASLCRKDCFFNEDFLWCVRATERSEDLAQLKQWVTILGAGRI

>sp|Q27225|MIH_CARMA Molt-inhibiting hormone OS=Carcinus maenas OX=6759 GN=MIH PE=1 SV=1

MMSRANSRFSCQRTWLLSVVVLAALWSFGVHRAAARVINDECPNLIGNRDLYKKVEWICE

DCSNIFRKTGMASLCRRNCFFNEDFVWCVHATERSEELRDLEEWVGILGAGRD

>sp|P55320|GIH_HOMAM Gonad-inhibiting hormone OS=Homarus americanus OX=6706 PE=1 SV=1

MVTRVGSGFSVQRVWLLLVIVVVLCGSVTQQASAWFTNDECPGVMGNRDLYEKVAWVCND

CANIFRNNDVGVMCKKDCFHTMDFLWCVYATERHGEIDQFRKWVSILRAGRK

>sp|Q62949|CORT_RAT Cortistatin OS=Rattus norvegicus OX=10116 GN=Cort PE=2 SV=1

MGGCSTRGKRPSALSLLLLLLLSGIAASALPLESGPTGQDSVQDATGGRRTGLLTFLAWW

HEWASQDSSSTAFEGGTPELSKRQERPPLQQPPHRDKKPCKNFFWKTFSSCK

>sp|Q9NL55|CHHL_BOMMO CHH-like protein OS=Bombyx mori OX=7091 GN=CHHL PE=2 SV=1

MHLSSVQFAWAALVALAVSAAGALPSSAPHHVERRSFFTLECKGVFDAAIFARLDRICDD

CFNLFREPQLYTLCRAECFTTPYFKGCMESLYLYDEKEQIDQMIDFVGKR

>sp|P56469|CORT_MOUSE Cortistatin OS=Mus musculus OX=10090 GN=Cort PE=2 SV=1

MMGGRGTGGKWPSAFGLLLLWGVAASALPLESGPTGQDSVQEATEGRSGLLTFLAWWHEW

ASQASSSTPVGGGTPGLSKSQERPPPQQPPHLDKKPCKNFFWKTFSSCK

>sp|Q23247|YH82_CAEEL Uncharacterized protein ZC168.2 OS=Caenorhabditis elegans OX=6239 GN=ZC168.2 PE=3 SV=1

MFVGPHRCFLISLIVYTLTVTTSSARLILEDTTVRSEEDSGPSCPVLADEPVGEVMERIC

DMCHELSSHSRPNMRVECRADCFTTDAFRECLKLFTPRRHTRHLRQKY

>sp|P01286|SLIB_HUMAN Somatoliberin OS=Homo sapiens OX=9606 GN=GHRH PE=1 SV=1

MPLWVFFFVILTLSNSSHCSPPPPLTLRMRRYADAIFTNSYRKVLGQLSARKLLQDIMSR

QQGESNQERGARARLGRQVDSMWAEQKQMELESILVALLQKHSRNSQG

>sp|Q5DW47|CORZ_APIME Pro-corazonin OS=Apis mellifera OX=7460 GN=Crz PE=1 SV=1

MVNSQILILFILSLTITIVMCQTFTYSHGWTNGKRSTSLEELANRNAIQSDNVFANCELQ

KLRLLLQGNINNQLFQTPCELLNFPKRSFSENMINDHRQPAPTNNNY

>sp|P01184|NEU2_BALPH Vasopressin-neurophysin 2 (Fragment) OS=Balaenoptera physalus OX=9770 GN=AVP PE=1 SV=1

CYFQNCPRGXXXAMSDLELRQCLPCGPGGKGRCFGPSICCGDELGCFMGTAEALRCQEEN

YLPSPCQSGQKPCGSGGRCAAAGICCNDESCVTEPECREGASFPRRA

>sp|P83636|MIH_FAXLI Molt-inhibiting hormone OS=Faxonius limosus OX=28379 PE=1 SV=2

MVNQVAQCFTVRRVWLVVVVGLLVHQTTARYVFEECPGVMGNRALHGKVTRVCEDCYNVF

RDTDVLAGCRKGCFSSEMFKLCLLAMERVEEFPDFKRWIGILNAGR

>sp|P63292|SLIB_BOVIN Somatoliberin OS=Bos taurus OX=9913 GN=GHRH PE=1 SV=1

MLLWVFFLVTLTLSSGSHGSLPSQPLRIPRYADAIFTNSYRKVLGQLSARKLLQDIMNRQ

QGERNQEQGAKVRLGRQVDGVWTDQQQMALESTLVSLLQERRNSQG

>sp|P55847|MIH_PENJP Molt-inhibiting hormone OS=Penaeus japonicus OX=27405 PE=1 SV=2

MYRLAMRTWLAIVIVVVGTSLLFDTASASFIDNTCRGVMGNRDIYKKVVRVCEDCTNIFR

LPGLDGMCRNRCFYNEWFLICLKAANREDEIEKFRVWISILNAGQ

>sp|O00230|CORT_HUMAN Cortistatin OS=Homo sapiens OX=9606 GN=CORT PE=1 SV=1

MPLSPGLLLLLLSGATATAALPLEGGPTGRDSEHMQEAAGIRKSSLLTFLAWWFEWTSQA

SAGPLIGEEAREVARRQEGAPPQQSARRDRMPCRNFFWKTFSSCK

>sp|O76534|MIH_METEN Probable molt-inhibiting hormone OS=Metapenaeus ensis OX=32278 PE=2 SV=1

MYRMPMRFWLTAVVMVVVGALLLDTASASYIENTCRGVMGNRDIYKKVVRVCEDCTNIFR

LPGLDGMCRDRCFNNEWFLVCLKAANRDDELDKFKVWISILNPGL

>sp|P01176|NEU1_HORSE Oxytocin-neurophysin 1 (Fragment) OS=Equus caballus OX=9796 GN=OXT PE=1 SV=2

CYIQNCPLGGKRAALDLDVRKCLPCGPGGKGRCFGPSICCGDELGCFVGTAEALRCQEEN

YLPSPCQSGQKPCGSGGRCAAAGICCSPDGCLADPSCHDEAAFSQ

>sp|P09916|SLIB_RAT Somatoliberin OS=Rattus norvegicus OX=10116 GN=Ghrh PE=1 SV=2

MPLWVFFVLLTLTSGSHCSLPPSPPFRVRRHADAIFTSSYRRILGQLYARKLLHEIMNRQ

QGERNQEQRSRFNRHLDRVWAEDKQMALESILQGFPRMKLSAEA

>sp|Q4R128|NTC1_CAEEL Nematocin OS=Caenorhabditis elegans OX=6239 GN=ntc-1 PE=1 SV=1

MGSSPILLVLAISIGLASACFLNSCPYRRYGRTIRCSSCGIENEGVCISEGRCCTNEECF

MSTECSYSAVCPELFCKIGHHPGYCMKKGYCCTQGGCQTSAMC

>sp|O09209|BXRA_AGRCO Bombyxin-related peptide A OS=Agrius convolvuli OX=55055 PE=2 SV=1

MKLLVVLCCFFAVYSLAAAQGGQEEFQIKVRICGRHLARTLADLCPNVEYEDVMKRSGAR

SPALYGTVGWPWARPGAARGKRAGVADDCCVNSCTMDVLLSYC

>sp|P16043|SLIB_MOUSE Somatoliberin OS=Mus musculus OX=10090 GN=Ghrh PE=3 SV=1

MLLWVLFVILILTSGSHCSLPPSPPFRMQRHVDAIFTTNYRKLLSQLYARKVIQDIMNKQ

GERIQEQRARLSRQEDSMWTEDKQMTLESILQGFPRMKPSADA

>sp|O96690|PDF_DROME Protein PDF OS=Drosophila melanogaster OX=7227 GN=Pdf PE=1 SV=2

MARYTYLVALVLLAICCQWGYCGAMAMPDEERYVRKEYNRDLLDWFNNVGVGQFSPGQVA

TLCRYPLILENSLGPSVPIRKRNSELINSLLSLPKNMNDAGK

>sp|P55322|MIH_PENVA Molt-inhibiting hormone-like (Fragment) OS=Penaeus vannamei OX=6689 PE=2 SV=1

LEKLLSSSSSSSGSSSPLDALGGDHSVNKRDTFDHSCKGIYDRELFRKLDRVCEDCYNVF

REPKVATECKSNCFVNKRFNVCVADLRHDVSRFLKMANSALS

>sp|O97385|CHH3_PENMO Crustacean hyperglycemic hormones 3 OS=Penaeus monodon OX=6687 GN=CHH3 PE=3 SV=1

MIALRLIAVTLVVAMAASTTWARSFNKRANFDPSCAGVYNRELLGRLSRLCDDCYNVFRE

PKVATECRNNCFYNPVFVQCLEYLIPADLHEEYQAHVQTVGK

>sp|I7C2V3|SPXN_CARAU Spexin prohormone 1 OS=Carassius auratus OX=7957 GN=spx PE=1 SV=2

MKDLRTLAAYALALLLLATFVSYSRSAPMGSFQRRNWTPQAMLYLKGTQGRRFVSEDRNE

GDLYDTIRLESQSQNTENLSISKAAAFLLNVLQQARDEGEPY

>sp|Q9VET0|NPF_DROME Neuropeptide F OS=Drosophila melanogaster OX=7227 GN=NPF PE=1 SV=1

MCQTMRCILVACVALALLAAGCRVEASNSRPPRKNDVNTMADAYKFLQDLDTYYGDRARV

RFGKRGSLMDILRNHEMDNINLGKNANNGGEFARGFNEEEIF

>sp|Q29B55|NPF_DROPS Neuropeptide F OS=Drosophila pseudoobscura pseudoobscura OX=46245 GN=npf PE=3 SV=3

MSNTMRCILIVCVALTLIAAGCNVEASNSRPPRKNDVNTMADAYKFLQDLDTYYGDRARV

RFGKRGGPLMEMLRNRELENNMAKSINSGGELIRALDEEEVF

>sp|F1QQI2|SPXN1_DANRE Spexin prohormone 1 OS=Danio rerio OX=7955 GN=spx PE=1 SV=1

MKDLRTLAAYALALLLLATFVSHSWSAPKGSFQRRNWTPQAMLYLKGTQGRRFVSEDRNE

GDLYDTIRLESRSQNTENLSISKAAAFLLNILQQARDEDEPY

>sp|Q86N75|CORZ_BOMMO Pro-corazonin OS=Bombyx mori OX=7091 GN=crz PE=3 SV=1

MVTNITLILTLMTLASVTAQTFQYSRGWTNGKRDGHKRDELRDEVLERILTPCQLDKLKY

VLEGKPLNDRLFVPCDYIEEEVNQPKRYKGERNHELFDVFQ

>sp|A0A4Y5X1A7|CESS3_CONMO Conopressin/conophysin, isoform 3 (Fragment) OS=Conus monile OX=351660 PE=1 SV=1

ACFIRNCPKGGKRNVDEGPTKPCMFCSFGQCVAPHTCCGEKGCEMGTVDANMCQEENESP

IPCHVFGKRCLLNHPGNSHGNCVTYGICCSHDTCTVHLACM

>sp|P10601|PAHO_MOUSE Pancreatic prohormone OS=Mus musculus OX=10090 GN=Ppy PE=3 SV=1

MAVAYCCLSLFLVSTWVALLLQPLQGTWGAPLEPMYPGDYATPEQMAQYETQLRRYINTL

TRPRYGKRAEEENTGGLPGVQLSPCTSPPVGLIPCSAPWS

>sp|P61849|NEMS_DROME Dromyosuppressin OS=Drosophila melanogaster OX=7227 GN=Ms PE=1 SV=1

MSFAQFFVACCLAIVLLAVSNTRAAVQGPPLCQSGIVEEMPPHIRKVCQALENSDQLTSA

LKSYINNEASALVANSDDLLKNYNKRTDVDHVFLRFGKRR

>sp|P07808|NPY_RAT Pro-neuropeptide Y OS=Rattus norvegicus OX=10116 GN=Npy PE=1 SV=1

MMLGNKRMGLCGLTLALSLLVCLGILAEGYPSKPDNPGEDAPAEDMARYYSALRHYINLI

TRQRYGKRSSPETLISDLLMRESTENAPRTRLEDPSMW

>sp|Q9EPS2|PYY_MOUSE Peptide YY OS=Mus musculus OX=10090 GN=Pyy PE=3 SV=2

MVAVRRPWPVTVAMLLILLACLGALVDAYPAKPEAPGEDASPEELSRYYASLRHYLNLVT

RQRYGKRDVPAALFSKLLFTDDSDSENLPFRPEGLDQW

>sp|P06303|PAHO_RAT Pancreatic prohormone OS=Rattus norvegicus OX=10116 GN=Ppy PE=3 SV=1

MAVAYYCLSLFLLSTWVALLLQPLQGAWGAPLEPMYPGDYATHEQRAQYETQLRRYINTL

TRPRYGKRDEDTAGLPGRQLPPCTSLLVGLMPCAAARS

>sp|P10631|PYY_RAT Peptide YY OS=Rattus norvegicus OX=10116 GN=Pyy PE=1 SV=1

MVAVRRPWPVMVAMLLVLLACLGALVDAYPAKPEAPGEDASPEELSRYYASLRHYLNLVT

RQRYGKREVPAALFSKLLFTDDSENLPFRSRPEGVDQW

>sp|P57774|NPY_MOUSE Pro-neuropeptide Y OS=Mus musculus OX=10090 GN=Npy PE=1 SV=2

MLGNKRMGLCGLTLALSLLVCLGILAEGYPSKPDNPGEDAPAEDMARYYSALRHYINLIT

RQRYGKRSSPETLISDLLMKESTENAPRTRLEDPSMW

>sp|Q9XSW6|NPY_MACMU Pro-neuropeptide Y OS=Macaca mulatta OX=9544 GN=NPY PE=1 SV=1

MLGSKRLGLSGLTLALSLLVCLGALAEAYPSKPDNPGEDAPAEDMARYYSALRHYINLIT

RQRYGKRSSPETLISDLLMRESTENVPRTRLEDPSMW

>sp|P01303|NPY_HUMAN Pro-neuropeptide Y OS=Homo sapiens OX=9606 GN=NPY PE=1 SV=1

MLGNKRLGLSGLTLALSLLVCLGALAEAYPSKPDNPGEDAPAEDMARYYSALRHYINLIT

RQRYGKRSSPETLISDLLMRESTENVPRTRLEDPAMW

>sp|P28673|NPY_CHICK Pro-neuropeptide Y OS=Gallus gallus OX=9031 GN=NPY PE=3 SV=1

MQGTMRLWVSVLTFALSLLICLGTLAEAYPSKPDSPGEDAPAEDMARYYSALRHYINLIT

RQRYGKRSSPETLISDLLLRESTENIPRSRFEDPSMW

>sp|Q07892|ECLH_DROME Eclosion hormone OS=Drosophila melanogaster OX=7227 GN=Eh PE=2 SV=1

MNCKPLILCTFVAVAMCLVHFGNALPAISHYTHKRFDSMGGIDFVQVCLNNCVQCKTMLG

DYFQGQTCALSCLKFKGKAIPDCEDIASIAPFLNALE

>sp|P51694|PYY_BOVIN Peptide YY OS=Bos taurus OX=9913 GN=PYY PE=3 SV=1

MMSGRRSWPAMATVLLTLLVCLGELVDAYPAKPQAPGEHASPDELNRYYTSLRHYLNLVT

RQRFGKRDFSEALLSILLFPDREDPPVKSRPEGAYLW

>sp|Q6RUW3|NPY_BOVIN Pro-neuropeptide Y OS=Bos taurus OX=9913 GN=NPY PE=3 SV=1

MLGSKRLGLSGLTLALSLLVCLGALAEAYPSKPDNPGEDAPAEDLARYYSALRHYINLIT

RQRYGKRSSPETLISDLLMRESTGNIPRTRLEDPSMW

>sp|V9QFH8|TXA2_STEGR Alpha-latrotoxin associated low molecular weight protein 2 OS=Steatoda grossa OX=256750 PE=3 SV=1

MFKLICIVFIATILSITSAADNEDELTIEDFLSYECNESMDIEELKEKDKVCSRCANLHK

TQSVIERCRLNCFTSEYFKNCEDNLQAKEEEPEEETL

>sp|P10082|PYY_HUMAN Peptide YY OS=Homo sapiens OX=9606 GN=PYY PE=1 SV=3

MVFVRRPWPALTTVLLALLVCLGALVDAYPIKPEAPREDASPEELNRYYASLRHYLNLVT

RQRYGKRDGPDTLLSKTFFPDGEDRPVRSRSEGPDLW

>sp|Q9I8P2|PYYA_DANRE Peptide YY-A OS=Danio rerio OX=7955 GN=pyya PE=2 SV=1

MAVMLKPWTVVATVLICVLLCLGTFVDAYPPKPENPGDDAAPEELAKYYTALRHYINLIT

RQRYGKRSTSEDVMAELLFGDDTEHKQRSRYDDSFMW

>sp|Q9I8P3|NPY_DANRE Pro-neuropeptide Y OS=Danio rerio OX=7955 GN=npy PE=3 SV=1

MNPNMKMWMSWAACAFLLFVCLGTLTEGYPTKPDNPGEDAPAEELAKYYSALRHYINLIT

RQRYGKRSSADTLISDLLIGETESRPQTRYEDHLAW

>sp|P01298|PAHO_HUMAN Pancreatic prohormone OS=Homo sapiens OX=9606 GN=PPY PE=1 SV=1

MAAARLCLSLLLLSTCVALLLQPLLGAQGAPLEPVYPGDNATPEQMAQYAADLRRYINML

TRPRYGKRHKEDTLAFSEWGSPHAAVPRELSPLDL

>sp|P35519|NEU1_ANSAN Neurophysin 1 OS=Anser anser anser OX=8844 PE=1 SV=1

AVLDGDVRKCLPCGPRNRGRCFGPRICCGEELGCYLGTPETLRCQEESFLPTPCESGRKP

CGGDGASCAAPGICCSSEGCVADPACEREALFA

>sp|P15444|NEU1_STRCA Neurophysin 1 OS=Struthio camelus OX=8801 PE=1 SV=1

AVLDMDIRKCMPCGPRNKGHCFGPNICCGEELGCYFGTSETLRCQEENFLPTPCESGRKP

CGNNEGSCAASGICCSNEGCMVDSSCDQEVMFP

>sp|O09210|BXRB_AGRCO Bombyxin-related peptide B OS=Agrius convolvuli OX=55055 PE=2 SV=1

MKFVLVLVSLALLVSLASVQGNNYCGRHLSETLAYMCPELEGASKRSGAMGAAAMYGTRG

WRWAAMGGNRGKRGVVEECCYQSCTLDELLTYC

>sp|P01299|PAHO_CANLF Pancreatic prohormone OS=Canis lupus familiaris OX=9615 GN=PPY PE=1 SV=3

MPAACRCLFLLLLSACVALLLQPPLGTRGAPLEPVYPGDDATPEQMAQYAAELRRYINML

TRPRYGKRDRGEMRDILEWGSPHAAAPRELMDE

>sp|P81768|NEU2_LOXAF Neurophysin 2 OS=Loxodonta africana OX=9785 GN=AVP PE=1 SV=1

AMSDMELRQCLPCGPGGKGRCFGPSICCGEELGCFVGTAEALRCQEENYLPSPCQSGQKP

CGSGGRCAAAGICCYEESCVTEPECREGAGIH

>sp|P01182|NEU2_HORSE Neurophysin 2 (Fragment) OS=Equus caballus OX=9796 GN=AVP PE=1 SV=1

DLELRQCLPCGPGGKGRCFGPSICCGDELGCFVGTAEALRCQEENYLPSPCQSGQKPCGS

GGRCAAAGICCNDESCVTEPECREGAGLPRRA

>sp|Q9VV28|NPLP3_DROME Neuropeptide-like 3 OS=Drosophila melanogaster OX=7227 GN=Nplp3 PE=1 SV=1

MFKLCVFVALLSLAAAAPAPAPAPAPAPGLIGPGIVAPGIWGPTVVGSPLLAPQVVSVVP

GAISHAAITQVHPSPLLIKSVHGLGPVVIG

>sp|Q7Q7R8|NPF_ANOGA Neuropeptide F OS=Anopheles gambiae OX=7165 GN=npf PE=1 SV=3

MASGTFTQRLLVALMIFALIADLSTLVAARPQDSDAASVAAAIRYLQELETKHAQHARPR

FGKRGGYLNPAIFGQDEQEVDWQDSTFSR

>sp|P25331|ECLH_BOMMO Eclosion hormone OS=Bombyx mori OX=7091 PE=1 SV=2

MANKLTAVIVVALAVAFMVNLDYANCSPAIASSYDAMEICIENCAQCKKMFGPWFEGSLC

AESCIKARGKDIPECESFASISPFLNKL

>sp|P11919|ECLH_MANSE Eclosion hormone OS=Manduca sexta OX=7130 PE=1 SV=2

MAGKVTVAFFMFAMIAFLANFGYVECNPAIATGYDPMEICIENCAQCKKMLGAWFEGPLC

AESCIKFKGKLIPECEDFASIAPFLNKL

>sp|Q4U4N3|TXA2_LATTR Alpha-latrotoxin-associated low molecular weight protein-2 OS=Latrodectus tredecimguttatus OX=6925 PE=1 SV=1

MLKLICIAFLVTVLTLVAGQDSLDPAEFGCADDVNQAELLKNNDICLQCEDLHKEGVVFS

LCKTNCFTTEYFQHCVKDLEEAKKEPPE

>sp|V9QEI7|TXA2_LATHE Alpha-latrotoxin associated low molecular weight protein 2 OS=Latrodectus hesperus OX=256737 PE=3 SV=1

MLKLICIAFLVTVLTLVAGEDSLDPAEYGCADDINQEDLLKKNDVCLQCEDLHKEGVVFS

LCKTNCFTTQYFTNCVKDLEEAEKEPPE

>sp|P81277|PRRP_HUMAN Prolactin-releasing peptide OS=Homo sapiens OX=9606 GN=PRLH PE=2 SV=1

MKVLRAWLLCLLMLGLALRGAASRTHRHSMEIRTPDINPAWYASRGIRPVGRFGRRRATL

GDVPKPGLRPRLTCFPLEGGAMSSQDG

>sp|Q9VU58|NPLP2_DROME Neuropeptide-like 2 OS=Drosophila melanogaster OX=7227 GN=Nplp2 PE=1 SV=1

MAKLAICILVFALFALALSARVPREESNPAQEFLTKAQGDFNEFIEKLKALDAKKVEGLF

KDGLNTVQEGLQKLNETFLQAPAAST

>sp|P32005|NEU1_PAPHA Oxytocin-neurophysin 1 (Fragment) OS=Papio hamadryas OX=9557 GN=OXT PE=2 SV=1

PLGGKRAAPDLDVRKCLPCGPGGKGRCFGPNICCAEELGCFVGTAEALRCQEENYLPSPC

QSGQKACGSGGRCAVFGLCCSPDGC

>sp|P58990|CXPH_CONRA Conophysin-R OS=Conus radiatus OX=61198 PE=1 SV=1

HPTKPCMYCSFGQCVGPHICCGPTGCEMGTAEANMCSEEDEDPIPCQVFGSDCALNNPDN

IHGHCVADGICCVDDTCTTHLGCL

>sp|P83627|VIH_ARMVU Vitellogenesis-inhibiting hormone OS=Armadillidium vulgare OX=13347 PE=1 SV=1

YNIPLGWGRRDMPGCLGVLGNRDLYDDVSRICSDCQNVFRDKNVESKCRSDCFSTSYFET

CIMALDLAEKISDYKLHASILKE

>sp|P81278|PRRP_RAT Prolactin-releasing peptide OS=Rattus norvegicus OX=10116 GN=Prlh PE=2 SV=1

MALKTWLLCLLLLSLVLPGASSRAHQHSMETRTPDINPAWYTGRGIRPVGRFGRRRATPR

DVTGLGQLSCLPLDGRTKFSQRG

>sp|Q26181|CHH_PENVA Crustacean hyperglycemic hormones (Fragment) OS=Penaeus vannamei OX=6689 PE=1 SV=1

AGLTKRSLFDPSCTGVFDRQLLRRLRRVCDDCFNVFREPNVSTECRSNCYNNEVFRQCME

YLLPPHLHEEHRLAVQMVGK

>sp|P06833|PYY2_BOVIN Caltrin OS=Bos taurus OX=9913 GN=PYY2 PE=1 SV=2

MMAGRRSWPAMATVLLALLVCLGELVDSKPQPSDEKASPDKHHRFSLSRYAKLANRLANP

KLLETFLSKWIGDRGNRSVK

>sp|P68248|PAHO_CHICK Pancreatic hormone OS=Gallus gallus OX=9031 GN=PPY PE=1 SV=1

MPPRWASLLLLACSLLLLAVPPGTAGPSQPTYPGDDAPVEDLIRFYNDLQQYLNVVTRHR

YGRRSSSRVLCEEPMGAAGC

>sp|P61855|AKH_DROME Adipokinetic hormone OS=Drosophila melanogaster OX=7227 GN=Akh PE=1 SV=1

MNPKSEVLIAAVLFMLLACVQCQLTFSPDWGKRSVGGAGPGTFFETQQGNCKTSNEMLLE

IFRFVQSQAQLFLDCKHRE

>sp|P81034|MOIH1_CANPG Mandibular organ-inhibiting hormone 1 OS=Cancer pagurus OX=6755 PE=1 SV=1

RRINNDCQNFIGNRAMYEKVDWICKDCANIFRKDGLLNNCRSNCFYNTEFLWCIDATENT

RNKEQLEQWAAILGAGWN

>sp|P55846|MIH_CANPG Molt-inhibiting hormone OS=Cancer pagurus OX=6755 PE=1 SV=1

RVINDDCPNLIGNRDLYKKVEWICEDCSNIFRNTGMATLCRKNCFFNEDFLWCVYATERT

EEMSQLRQWVGILGAGRE

>sp|P81035|MOIH2_CANPG Mandibular organ-inhibiting hormone 2 OS=Cancer pagurus OX=6755 PE=1 SV=1

RRINNDCQNFIGNRAMYEKVDWICKDCANIFRQDGLLNNCRSNCFYNTEFLWCIDATENT

RNKEQLEQWAAILGAGWN

>sp|P01304|NPY_PIG Pro-neuropeptide Y (Fragment) OS=Sus scrofa OX=9823 GN=NPY PE=1 SV=2

VCLCALAEAYPSKPDNPGEDAPAEDLARYYSALRHYINLITRQRYGKRSSPETLISDLLM

REGTENVPRTRLEDPS

>sp|P01284|VIP_PIG VIP peptides (Fragment) OS=Sus scrofa OX=9823 GN=VIP PE=1 SV=2

HADGVFTSDFSRLLGQLSAKKYLESLIXXXXXXXXXXXXXXXXXHSDAVFTDNYTRLRKQ

MAVKKYLNSILNGKR

>sp|P55848|MIH_PROCL Molt-inhibiting hormone OS=Procambarus clarkii OX=6728 PE=1 SV=1

RYVFEECPGVMGNRAVHGKVTRVCEDCYNVFRDTDVLAGCRKGCFSSEMFKLCLLAMERV

EEFPDFKRWIGILNA

>sp|P83220|MIH_JASLA Probable molt-inhibiting hormone OS=Jasus lalandii OX=99572 PE=1 SV=1

RFTFDCPGMMGQRYLYEQVEQVCDDCYNLYREEKIAVNCRENCFLNSWFTVCLQATMREH

ETPRFDIWRSILKA

>sp|Q10987|MIH_PROBO Molt-inhibiting hormone (Fragment) OS=Procambarus bouvieri OX=6729 PE=1 SV=2

QVFDQACKGIYDRAIFKKLELVCDDCYNLYRKPKVATTCRENCYANSVFRQCLDDLLLIN

VVDEYISGVQIVGK

>sp|P56687|CHH_JASLA Crustacean hyperglycemic hormone OS=Jasus lalandii OX=99572 PE=1 SV=1

AVFDQSCKGVYDRSLFSKLDRVCDDCYNLYRKHYVATGCRRNCYGNLVFRQCLDDLMLVD

VVDEYYVASVQMV

>sp|P30814|CHH_ARMVU Crustacean hyperglycemic hormone OS=Armadillidium vulgare OX=13347 PE=1 SV=1

RIFDTSCKGFYDRGLFAQLDRVCEDCYNLYRKPHVAAECRRDCYTTEVFESCLKDLMMHD

FINEYKEMALMVS

>sp|P83485|CHHA_CHEDE Crustacean hyperglycemic hormone A OS=Cherax destructor OX=6723 PE=1 SV=1

QVFDQACKGVYDRAIFKKLDRVCDDCYNLYRKPYVAVSCRGNCYNNLVFRQCLEELFLGN

GFNEYISGVQTV

>sp|P59685|CHH_PENSC Crustacean hyperglycemic hormone OS=Penaeus schmitti OX=122378 PE=1 SV=1

ANFDPSCTGVYDRELLGRLSRLCDDCYNVFREPKVATECRSNCFYNPVFVQCLEYLIPAD

LHEEYQAHVQTV

>sp|P81032|CHH_CANPG Crustacean hyperglycemic hormone OS=Cancer pagurus OX=6755 PE=1 SV=1

QIYDTSCKGVYDRGLFSDLEHVCDDCYNLYRNSYVASACRSNCYSNVVFRQCMEELLLMD

EFDKYARAVQIV

>sp|P81700|CHH6_PENJP Crustacean hyperglycemic hormone 6 OS=Penaeus japonicus OX=27405 PE=1 SV=1

LVFDPSCAGVYDRVLLGKLNRLCDDCYNVFREPNVATECRSNCFYNLAFVQCLEYLLPPS

LHEEYQANVQMV

>sp|P83486|CHHB_CHEDE Crustacean hyperglycemic hormone B OS=Cherax destructor OX=6723 PE=1 SV=1

QVFDQACKGVYDRAIFKKLDRVCDDCYNLYRKPYVATSCRQNCYSNLVFRQCLDDLLLVD

VVDEYVSGVQIV

>sp|P83800|CHH_ASTAS Crustacean hyperglycemic hormone OS=Astacus astacus OX=6715 PE=1 SV=1

QVFDQACKGIYDRAIFKKLDRVCDDCYNLYRKPYVATTCRQNCYANSVFRQCLDDLLLID

VVDEYISGVQIV

>sp|P55845|CHH_PROBO Crustacean hyperglycemic hormone OS=Procambarus bouvieri OX=6729 PE=1 SV=1

QVFDQACKGIYDRAIFKKLDRVCEDCYNLYRKPYVATTCRQNCYANSVFRQCLDDLLLID

VVDEYISGVQTV

>sp|Q6IGX9|SIFA_DROME Neuropeptide SIFamide OS=Drosophila melanogaster OX=7227 GN=SIFa PE=1 SV=1

MALRFTLTLLLVTILVAAILLGSSEAAYRKPPFNGSIFGKRNSLDYDSAKMSAVCEVAME

ACPMWFPQNDSK

>sp|P81206|CHH1_MACRS Crustacean hyperglycemic hormone isoform 1 OS=Macrobrachium rosenbergii OX=79674 PE=1 SV=1

AILDQSCKGIFDRELFKKLDRVCDDCYNLYRKPYVAIDCRRGCYQNLVFRQCIQDLQLMD

DLDEYANAVQV

>sp|A0A077DF94|SPXN2_DANRE Spexin prohormone 2 (Fragment) OS=Danio rerio OX=7955 GN=spx2 PE=2 SV=1

MISRVWILWTLVLFLLVTESHCIQKSSLSKNWGPQSMLYLKGKHGRRFVPDIDDHFISNS

GLKSWYAVFK

>sp|Q5JQD4|PYY3_HUMAN Putative peptide YY-3 OS=Homo sapiens OX=9606 GN=PYY3 PE=5 SV=1

MVSVCRPWPAVAIALLALLVCLGALVDTCPIKPEAPGEDESLEELSHYYASLCHYLNVVT

RQWWEGADMW

>sp|P06884|PAHO_FELCA Pancreatic prohormone (Fragment) OS=Felis catus OX=9685 GN=PPY PE=1 SV=1

APLEPVYPGDNATPEQMAQYAAELRRYINMLTRPRYGKRDRGETLDILEWGSPHAAAPRE

LSPMDV

>sp|Q9BDP9|CART_PIG Cocaine- and amphetamine-regulated transcript protein (Fragment) OS=Sus scrofa OX=9823 GN=CARTPT PE=2 SV=1

HEKELIEALQEVLKKLKSKRIPIYEKKYGQVPMCDAGEQCAVRKGARIGKLCDCPRGTSC

NSFLLK

>sp|P01300|PAHO_PIG Pancreatic prohormone precursor (Fragment) OS=Sus scrofa OX=9823 GN=PPY PE=1 SV=2

APLEPVYPGDDATPEQMAQYAAELRRYINMLTRPRYGKRDEEDLLDLKCSSLHAAAPREL

SPMGA

>sp|Q9VQ66|NPLP4_DROME Neuropeptide-like 4 OS=Drosophila melanogaster OX=7227 GN=Nplp4 PE=1 SV=1

MFKLLVVVFAALFAAALAVPAPVARANPAPIPIASPEPAPQYYYGASPYAYSGGYYDSPY

SYYG

>sp|Q9TS44|CCKN_CANLF Cholecystokinins OS=Canis lupus familiaris OX=9615 GN=CCK PE=1 SV=1

AVQKVDGEPRAHLGALLARYIQQARKAPSGRMSVIKNLQNLDPSHRISDRDYMGWMDF

>sp|Q9XZE6|ECLH_ROMMI Eclosion hormone (Fragment) OS=Romalea microptera OX=7007 PE=2 SV=1

CKKMVGAYFEGELCADACLKFKGKMCPTARTSPPSRPSSTSLSSRCCSIKSLCKA

>sp|P33710|GALA_CANLF Galanin peptides (Fragment) OS=Canis lupus familiaris OX=9615 GN=GAL PE=2 SV=1

LNSAGYLLGPHAIDNHRSFHEKPGLTGKRELPPEDEGRSGGFAGPLSLSENAAV

>sp|P01287|SLIB_PIG Somatoliberin OS=Sus scrofa OX=9823 GN=GHRH PE=1 SV=1

YADAIFTNSYRKVLGQLSARKLLQDIMSRQQGERNQEQGARVRL

>sp|P29203|PYY_CHICK Peptide YY-like OS=Gallus gallus OX=9031 GN=PYY PE=1 SV=1

AYPPKPESPGDAASPEEIAQYFSALRHYINLVTRQRY

>sp|P68004|PYY_CANLF Peptide YY OS=Canis lupus familiaris OX=9615 GN=PYY PE=1 SV=1

YPAKPEAPGEDASPEELSRYYASLRHYLNLVTRQRY

>sp|P68005|PYY_PIG Peptide YY OS=Sus scrofa OX=9823 GN=PYY PE=1 SV=1

YPAKPEAPGEDASPEELSRYYASLRHYLNLVTRQRY

>sp|P33684|PAHO_MACMU Pancreatic hormone OS=Macaca mulatta OX=9544 GN=PPY PE=1 SV=1

APLEPVYPGDNATPEQMAQYAADLRRYINMLTRPRY

>sp|P43511|PBAN_LYMDI Pheromone biosynthesis-activating neuropeptide OS=Lymantria dispar OX=13123 PE=1 SV=1

LADDMPATMADQEVYRPEPEQIDSRNKYFSPRL

>sp|P56717|OREX_BOVIN Orexin-A OS=Bos taurus OX=9913 GN=HCRT PE=1 SV=1

QPLPDCCRQKTCSCRLYELLHGAGNHAAGILTL

>sp|P01297|NMB_PIG Neuromedin-B OS=Sus scrofa OX=9823 GN=NMB PE=1 SV=1

APLSWDLPEPRSRAGKIRVHPRGNLWATGHFM

>sp|P42992|NEUV_SCLRE Vasotocin-neurophysin VT (Fragment) OS=Sclerophrys regularis OX=1978144 PE=1 SV=1

CYIQNCPRGGKRSYPDTAVPQCIPCGP

>sp|P83683|OX26_PELLE Orexigenic neuropeptide 26RFa OS=Pelophylax lessonae OX=45623 PE=1 SV=1

VGTALGSLAEELNGYNRKKGGFSFRF

>sp|P85741|PPK5_POLAY Pyrokinin-5 OS=Polyphaga aegyptiaca OX=7085 PE=1 SV=1

SASGGAGESSGMWFGPRL

>sp|P25271|PHPT_MYTSE Pheromonotropin OS=Mythimna separata OX=271217 PE=1 SV=1

KLSYDDKVFENVEFTPRL

>sp|P85755|PPK5_PSEFV Pyrokinin-5 OS=Pseudoderopeltis flavescens OX=303916 PE=1 SV=1

GGGGGSGETSGMWFGPRL

>sp|P85790|PPK5_THEPT Pyrokinin-5 OS=Therea petiveriana OX=45965 PE=1 SV=1

SASGSGESSGMWFGPRL

>sp|P85569|PPK5_CRYDW Pyrokinin-5 OS=Cryptocercus darwini OX=89835 PE=1 SV=1

GGGGSGETSGMWFGPRL

>sp|P85656|PPK5_LAXSS Pyrokinin-5 OS=Laxta sp. (strain SR-2005) OX=348757 PE=1 SV=1

GGETSGETKGMWFGPRL

>sp|P85773|PPK5_SHELA Pyrokinin-5 OS=Shelfordella lateralis OX=36981 PE=1 SV=1

GGGGSGETSGMWFGPRL

>sp|P85690|PPK5_PANSB Pyrokinin-5 OS=Panesthia sp. (strain BF-2008) OX=521518 PE=1 SV=1

GGETSGEGKGMWFGPRL

>sp|P85720|PPK5_PERRU Pyrokinin-5 OS=Perisphaeria ruficornis OX=521516 PE=1 SV=1

SGETSGEGNGMWFGPRL

>sp|P82696|PH3_PERAM Peptide hormone 3 OS=Periplaneta americana OX=6978 PE=1 SV=1

AFLTLTPGSHVDSYVEA

>sp|P85617|PPK5_EUBDI Pyrokinin-5 OS=Eublaberus distanti OX=424761 PE=1 SV=1

AGESSNEAKGMWFGPRL

>sp|P84664|PPK5_RHYMA Pyrokinin-5 OS=Rhyparobia maderae OX=36963 PE=1 SV=1

FGETSGETKGMWFGPRL

>sp|P84671|PPK5_DERVE Pyrokinin-5 OS=Derocalymma versicolor OX=344692 PE=1 SV=1

TGDMSGEGKGMWFGPRL

>sp|P85559|PPK5_BLAOR Pyrokinin-5 OS=Blatta orientalis OX=6976 PE=1 SV=1

GGGGSGETSGMWFGPRL

>sp|P85710|PPK5_PERBR Pyrokinin-5 OS=Periplaneta brunnea OX=36976 PE=1 SV=1

GGGGSGETSGMWFGPRL

>sp|P84667|PPK5A_PYCSU Pyrokinin-5a OS=Pycnoscelus surinamensis OX=36961 PE=1 SV=1

GGETSGEGKGMWFGPRL

>sp|P85646|PPK5_GYNCS Pyrokinin-5 OS=Gyna cf. cafforum (strain SR-2005) OX=348763 PE=1 SV=1

AGDTSSEAKGMWFGPRL

>sp|P85702|PPK5_PERAU Pyrokinin-5 OS=Periplaneta australasiae OX=36975 PE=1 SV=1

GGGGSGETSGMWFGPRL

>sp|P85590|PPK5_DERIN Pyrokinin-5 OS=Deropeltis integerrima OX=596121 PE=1 SV=1

GGGGSGETSGMWFGPRL

>sp|P85685|PPK5_NEORO Pyrokinin-5 OS=Neostylopyga rhombifolia OX=304879 PE=1 SV=1

GGGGSGETSGMWFGPRL

>sp|P84595|PPK5_ARCTE Pyrokinin-5 OS=Archimandrita tessellata OX=36945 PE=1 SV=1

EGANSNEAKGMWFGPRL

>sp|P84670|PPK5_PANVI Pyrokinin-5 OS=Panchlora viridis OX=344693 PE=1 SV=1

GGETGSDAKAMWFGPRL

>sp|P85751|PPK5_PSEBJ Pyrokinin-5 OS=Pseudoderopeltis cf. bimaculata JT-2004 OX=304880 PE=1 SV=1

GGGGSGETSGMWFGPRL

>sp|P85564|PPK5_BLEDI Pyrokinin-5 OS=Blepharodera discoidalis OX=521524 PE=1 SV=1

SGETSGEGNGMWFGPRL

>sp|P85708|PPK5_PERBB Pyrokinin-5 OS=Perisphaeria aff. bicolor (strain BF-2008) OX=521515 PE=1 SV=1

SGETSGEGNGMWFGPRL

>sp|P85579|PPK5_CYRPO Pyrokinin-5 OS=Cyrtotria poduriformis OX=344688 PE=1 SV=1

SGETSGEGNGMWFGPRL

>sp|P85641|PPK5_GROPO Pyrokinin-5 OS=Gromphadorhina portentosa OX=36953 PE=1 SV=1

FGETSGETKGMWFGPRL

>sp|P85726|PPK5_PERSS Pyrokinin-5 OS=Perisphaeria cf. scabrella (strain SR-2005) OX=348759 PE=1 SV=1

SGETSGEGNGMWFGPRL

>sp|P85781|PPK5_SUPLO Pyrokinin-5 OS=Supella longipalpa OX=83902 PE=1 SV=1

GGGSSGETNGMWFGPRL

>sp|P85777|PPK5_SUPDI Pyrokinin-5 OS=Supella dimidiata OX=521517 PE=1 SV=1

GGGSSGETNGMWFGPRL

>sp|P85650|PPK5_GYNLU Pyrokinin-5 OS=Gyna lurida OX=406578 PE=1 SV=1

AGDTSSEAKGMWFGPRL

>sp|P85545|PPK5_BLACR Pyrokinin-5 OS=Blaberus craniifer OX=6982 PE=1 SV=1

AGESSNEAKGMWFGPRL

>sp|P84668|PPK5B_PYCSU Pyrokinin-5b OS=Pycnoscelus surinamensis OX=36961 PE=1 SV=1

GGETGGEGKGMWFGPRL

>sp|P85747|PPK5_PRIVA Pyrokinin-5 OS=Princisia vanwaerebeki OX=1661849 PE=1 SV=1

FGETSGETKGMWFGPRL

>sp|P85759|PPK5_PSEFO Pyrokinin-5 OS=Pseudoderopeltis foveolata OX=303879 PE=1 SV=1

GGGGSGETSGMWFGPRL

>sp|P85669|PPK5_LUCSU Pyrokinin-5 OS=Lucihormetica subcincta OX=406666 PE=1 SV=1

GGESSNEAKGMWFGPRL

>sp|P85556|PPK5_BLAGI Pyrokinin-5 OS=Blaberus giganteus OX=36943 PE=1 SV=1

AGESSNEAKGMWFGPRL

>sp|P85629|PPK5_EUBSB Pyrokinin-5 OS=Eublaberus sp. (strain BF-2008) OX=521510 PE=1 SV=1

AGESSNEAKGMWFGPRL

>sp|P85596|PPK5_DERAT Pyrokinin-5 OS=Deropeltis atra OX=596120 PE=1 SV=1

GGGGSGETSGMWFGPRL

>sp|P85696|PPK5_PANSS Pyrokinin-5 OS=Panchlora sp. (strain SR-2005) OX=348758 PE=1 SV=1

GGETGNDAKAMWFGPRL

>sp|P85554|PPK5_BLAGE Pyrokinin-5 OS=Blattella germanica OX=6973 PE=1 SV=1

ESGGSGEANGMWFGPRL

>sp|P85730|PPK5_PERSR Pyrokinin-5 OS=Perisphaeria cf. substylifera (strain SR-2005) OX=348760 PE=1 SV=1

SGETSGEGNGMWFGPRL

>sp|P84666|PPK5_APTFU Pyrokinin-5 OS=Aptera fusca OX=344696 PE=1 SV=1

SGDTSSQAKGMWFGPRL

>sp|P85659|PPK5_LOBDE Pyrokinin-5 OS=Loboptera decipiens OX=242713 PE=1 SV=1

GSGGSGEANGMWFGPRL

>sp|P85638|PPK5_GROGR Pyrokinin-5 OS=Gromphadorhina grandidieri OX=521511 PE=1 SV=1

FGETSGETKGMWFGPRL

>sp|P85548|PPK5B_BLADU Pyrokinin-5b OS=Blaptica dubia OX=132935 PE=1 SV=1

GGESSNEAKGMWFGPRL

>sp|P85784|PPK5_SYMPA Pyrokinin-5 OS=Symploce pallens OX=36974 PE=1 SV=1

EGGSSGEASGMWFGPRL

>sp|P82617|PPK5_PERAM Pyrokinin-5 OS=Periplaneta americana OX=6978 PE=1 SV=1

GGGGSGETSGMWFGPRL

>sp|P85574|PPK5_CRYKY Pyrokinin-5 OS=Cryptocercus kyebangensis OX=161578 PE=1 SV=1

EGSGSGETSGMWFGPRL

>sp|P85796|PPK5_HOSCA Pyrokinin-5 OS=Hostilia carinata OX=645593 PE=1 SV=1

SGETSGEGNGMWFGPRL

>sp|P84669|PPK5_LAXSP Pyrokinin-5 OS=Laxta sp. (strain Australia) OX=344974 PE=1 SV=1

GGETSGETKGMWFGPRL

>sp|P85714|PPK5_PERFU Pyrokinin-5 OS=Periplaneta fuliginosa OX=36977 PE=1 SV=1

GGGGSGETSGMWFGPRL

>sp|P85733|PPK5_PERVR Pyrokinin-5 OS=Perisphaeria virescens OX=344690 PE=1 SV=1

SGETSGEGNGMWFGPRL

>sp|P84594|PPK5A_BLADU Pyrokinin-5a OS=Blaptica dubia OX=132935 PE=1 SV=1

AGESSNEAKGMWFGPRL

>sp|P85663|PPK5_LUCGR Pyrokinin-5 OS=Lucihormetica grossei OX=521513 PE=1 SV=1

GGESSNEAKGMWFGPRL

>sp|P85584|PPK5_DERER Pyrokinin-5 OS=Deropeltis erythrocephala OX=303918 PE=1 SV=1

GGGGSGETSGMWFGPRL

>sp|P84665|PPK5_DIPPU Pyrokinin-5 OS=Diploptera punctata OX=6984 PE=1 SV=1

SGETSGEGNGMWFGPRL

>sp|P85681|PPK5_MASDA Pyrokinin-5 OS=Mastotermes darwiniensis OX=13139 PE=1 SV=1

GGSGSGETSGMWFGPRL

>sp|P85675|PPK5_LUCVE Pyrokinin-5 OS=Lucihormetica verrucosa OX=521514 PE=1 SV=1

GGESSNEAKGMWFGPRL

>sp|P84672|PPK5_DERCR Pyrokinin-5 OS=Derocalymma cruralis OX=344972 PE=1 SV=1

DGDMSGEGKGMWFGPRL

>sp|P85623|PPK5_EUBPO Pyrokinin-5 OS=Eublaberus posticus OX=36951 PE=1 SV=1

AGESSNEAKGMWFGPRL

>sp|P85607|PPK5_ELLSS Pyrokinin-5 OS=Elliptorhina sp. (strain SR-2005) OX=348767 PE=1 SV=1

FGETSGETKGMWFGPRL

>sp|P85633|PPK5_EURFL Pyrokinin-5 OS=Eurycotis floridana OX=303877 PE=1 SV=1

GGGGSGETSGMWFGPRL

>sp|P85737|PPK5_PILDU Pyrokinin-5 OS=Pilema dubia OX=521525 PE=1 SV=1

SGETSGEGNGMWFGPRL

>sp|P85542|PPK5_BANRO Pyrokinin-5 OS=Bantua robusta OX=344686 PE=1 SV=1

SGETSGEGNGMWFGPRL

>sp|P85611|PPK5_ERGCA Pyrokinin-5 OS=Ergaula capucina OX=76901 PE=1 SV=1

SASSGESSGMWFGPRL

>sp|B3A079|PPK4_LOBRE CAPA-Pyrokinin OS=Lobatophasma redelinghuysense OX=253128 PE=1 SV=1

SSGGGDGSGMWFGPRL

>sp|B3A0J4|PPK5_PACBA CAPA-Pyrokinin OS=Pachyphasma brandbergense OX=1041430 PE=1 SV=1

NSGGGEGSGMWFGPRL

>sp|B3A0D7|PPK4_AUSGA CAPA-Pyrokinin OS=Austrophasma gansbaaiense OX=253136 PE=1 SV=1

SSGGGEGSGMWFGPRL

>sp|P20404|LPK1_LOCMI Locustapyrokinin-1 OS=Locusta migratoria OX=7004 PE=1 SV=3

QDSGDGWPQQPFVPRL

>sp|B0M3E5|PPK4_KARBO CAPA-Pyrokinin OS=Karoophasma botterkloofense OX=253132 PE=1 SV=1

SGGGDGSGMWFGPRL

>sp|P82695|PH2_PERAM Peptide hormone 2 OS=Periplaneta americana OX=6978 PE=1 SV=1

LDLTPGSHVDSYVEA

>sp|P42559|ALLS_MANSE Allatostatin OS=Manduca sexta OX=7130 PE=1 SV=1

QVRFRQCYFNPISCF

>sp|B3A098|PPK4_AUSRA CAPA-Pyrokinin OS=Austrophasma rawsonvillense OX=253137 PE=1 SV=1

SGGGEGSGMWFGPRL

>sp|P84357|PPK_MUSDO Pyrokinin OS=Musca domestica OX=7370 PE=1 SV=1

AGPSATTGVWFGPRL

>sp|B3EWL1|PK1_DELRA CAPA-Pyrokinin OS=Delia radicum OX=30064 PE=1 SV=1

AGPSATTGVWFGPRL

>sp|P84356|PPK_SARBU Pyrokinin OS=Sarcophaga bullata OX=7385 PE=1 SV=1

AGPSATTGVWFGPRL

>sp|B3A0H4|PPK5_TYRGL CAPA-Pyrokinin OS=Tyrannophasma gladiator OX=270861 PE=1 SV=1

SGGGEGSGMWFGPRL

>sp|B3A0F6|PPK5_PRAMA CAPA-Pyrokinin OS=Praedatophasma maraisi OX=409170 PE=1 SV=1

SGGGEGSGMWFGPRL

>sp|B0M3C4|PPK4_MANKU CAPA-Pyrokinin OS=Mantophasma kudubergense OX=1037657 PE=1 SV=1

SGGGEGSGMWFGPRL

>sp|B0M398|PPK4_STRNA CAPA-Pyrokinin OS=Striatophasma naukluftense OX=1041429 PE=1 SV=1

SGGGEGSGMWFGPRL

>sp|B3A0B8|PPK4_HEMMO CAPA-Pyrokinin OS=Hemilobophasma montaguense OX=253130 PE=1 SV=1

SGGGDGSGMWFGPRL

>sp|P86996|PPK4_NAMOO CAPA-Pyrokinin OS=Namaquaphasma ookiepense OX=409167 PE=1 SV=1

SGGGEGSGMWFGPRL

>sp|B3A060|PPK4_KARBI CAPA-Pyrokinin OS=Karoophasma biedouwense OX=253133 PE=1 SV=1

SGGGDGSGMWFGPRL

>sp|P46980|MY14_AMYVI Myoactive tetradecapeptide OS=Amynthas vittatus OX=506674 PE=1 SV=1

GFRDGSADRISHGF

>sp|P84361|PPK6_NEORO Pyrokinin-6 OS=Neostylopyga rhombifolia OX=304879 PE=1 SV=1

SDPEVPGMWFGPRL

>sp|P84362|PPK6_CELBM Pyrokinin-6 OS=Celatoblatta sp. (strain Blue Mountains) OX=303880 PE=1 SV=1

SDPEVPGMWFGPRL

>sp|P84370|PPK6_EURFL Pyrokinin-6 OS=Eurycotis floridana OX=303877 PE=1 SV=1

GDSEVPGMWFGPRL

>sp|P82965|ADFA_TENMO Antidiuretic factor A OS=Tenebrio molitor OX=7067 PE=1 SV=1

VVNTPGHAVSYHVY

>sp|P84366|PPK6_PSEFV Pyrokinin-6 OS=Pseudoderopeltis flavescens OX=303916 PE=1 SV=1

SDPEVPGMWFGPRL

>sp|P84365|PPK6_DERKE Pyrokinin-6 OS=Deropeltis sp. (strain Kenya) OX=303920 PE=1 SV=1

SDPEVPGMWFGPRL

>sp|P84371|PPK6_PSEFO Pyrokinin-6 OS=Pseudoderopeltis foveolata OX=303879 PE=1 SV=1

SDPEAPGIWFGPRL

>sp|P84367|PPK6_PSEBJ Pyrokinin-6 OS=Pseudoderopeltis cf. bimaculata JT-2004 OX=304880 PE=1 SV=1

SDPEVPGMWFGPRL

>sp|P84364|PPK6_DEREJ Pyrokinin-6 OS=Deropeltis cf. erythrocephala JT-2004 OX=303919 PE=1 SV=1

SDPEVPGMWFGPRL

>sp|P46979|MY14_EISFE Myoactive tetradecapeptide OS=Eisenia fetida OX=6396 PE=1 SV=1

GFKDGAADRISHGF

>sp|P84359|PPK6_PERBR Pyrokinin-6 OS=Periplaneta brunnea OX=36976 PE=1 SV=1

SDPEVPGMWFGPRL

>sp|P84363|PPK6_DERER Pyrokinin-6 OS=Deropeltis erythrocephala OX=303918 PE=1 SV=1

SDPEVPGMWFGPRL

>sp|P82693|PPK6_PERAM Pyrokinin-6 OS=Periplaneta americana OX=6978 PE=1 SV=1

SESEVPGMWFGPRL

>sp|P84368|PPK6_PERAU Pyrokinin-6 OS=Periplaneta australasiae OX=36975 PE=1 SV=1

NDPEVPGMWFGPRL

>sp|P84421|PPK6_SHELA Pyrokinin-6 OS=Shelfordella lateralis OX=36981 PE=1 SV=1

SESEVPGMWFGPRL

>sp|P84360|PPK6_PERFU Pyrokinin-6 OS=Periplaneta fuliginosa OX=36977 PE=1 SV=1

SDPEVPGMWFGPRL

>sp|P84420|PPK6_BLAOR Pyrokinin-6 OS=Blatta orientalis OX=6976 PE=1 SV=1

SESEVPGMWFGPRL

>sp|P41490|LMT4_LOCMI Locustamyotropin-4 OS=Locusta migratoria OX=7004 PE=1 SV=1

RLHQNGMPFSPRL

>sp|P11496|CORZ_PERAM Corazonin OS=Periplaneta americana OX=6978 PE=1 SV=2

QTFQYSRGWTN

>sp|P83382|PVK1_LOCMI Periviscerokinin-1 OS=Locusta migratoria OX=7004 PE=1 SV=1

AAGLFQFPRV

>sp|P69044|NEU1_HIPAM Oxytocin OS=Hippopotamus amphibius OX=9833 GN=OXT PE=1 SV=1

CYIQNCPLG

>sp|P69056|NEU1_BALPH Oxytocin OS=Balaenoptera physalus OX=9770 GN=OXT PE=1 SV=1

CYIQNCPLG

>sp|P69043|NEU1_RABIT Oxytocin OS=Oryctolagus cuniculus OX=9986 GN=OXT PE=1 SV=1

CYIQNCPLG

>sp|P69058|OXYT_HYDCO Oxytocin OS=Hydrolagus colliei OX=7873 PE=1 SV=1

CYIQNCPLG

>sp|P69128|OXYT_CYPCA Vasotocin OS=Cyprinus carpio OX=7962 PE=1 SV=1

CYIQNCPRG

>sp|P05487|CONO_CONST Conopressin-S OS=Conus striatus OX=6493 PE=1 SV=1

CIIRNCPRG

>sp|P16339|DNF1_LOCMI Locupressin OS=Locusta migratoria OX=7004 PE=1 SV=1

CLITNCPRG

>sp|P42993|ISOT_CYPCA Isotocin OS=Cyprinus carpio OX=7962 PE=1 SV=1

CYISNCPIG

>sp|P69057|NEU1_TACAC Oxytocin OS=Tachyglossus aculeatus aculeatus OX=49271 GN=OXT PE=1 SV=1

CYIQNCPLG

>sp|P42994|OXYT_RAJCL Glumitocin OS=Raja clavata OX=7781 PE=1 SV=1

CYISNCPQG

>sp|P80027|OXYT_OCTVU Cephalotocin OS=Octopus vulgaris OX=6645 PE=1 SV=1

CYFRNCPIG

>sp|P43000|OXYV_SQUAC Valitocin OS=Squalus acanthias OX=7797 PE=1 SV=1

CYIQNCPVG

>sp|P41489|LMT3_LOCMI Locustamyotropin-3 OS=Locusta migratoria OX=7004 PE=1 SV=1

RQQPFVPRL

>sp|P42997|OXYF_SCYCA Phasvatocin OS=Scyliorhinus canicula OX=7830 PE=1 SV=1

CYFNNCPVG

>sp|P42996|OXYA_SCYCA Asvatocin OS=Scyliorhinus canicula OX=7830 PE=1 SV=1

CYINNCPVG

>sp|P42999|OXYA_SQUAC Aspartocin OS=Squalus acanthias OX=7797 PE=1 SV=1

CYINNCPLG

>sp|P69129|OXYT_PETMA Vasotocin OS=Petromyzon marinus OX=7757 PE=1 SV=1

CYIQNCPRG

>sp|P42998|OXYT_EISFE Annetocin OS=Eisenia fetida OX=6396 PE=1 SV=1

CFVRNCPTG

>AalVWamide

MQLSSVRVGFVVLLCHLVVGAFCNTETQIKGASPPASQKREAAKSNVVSDREDALMKAIEKGIFARRSVSSQPPGV
WGRSLESSMKAREAKRDFHMLRPQSPAERRALQDSIHHLLQAKEALDDLTKRISQPPGVWGKRMASQPPGVWG
KRNVRTQPPGVWGKRSMKTQPPGVWGKRSSELFDRGYNVREAENTEEEDEIDTRSAVVRQPPGVWGRRDIELA
YRDEQ

>AalRFamide

MELKIKMLAIALFSILLIHSICAEDPKQQRSLSQGETDSQPFQADDDNAVDDRSIEKAKRIIEQWLRGRFGKREQWL
RGRFGRGVSKKETPPENKAREALEQWLRGRFGREASDQWLRGRFGRELMEQWLRGRFGREDDSQWLRGRFGK
EVSEQGRFAREAITQWLRGRFGREIQSQEEEERISRELEQWLRGRFGREDSQWLRGRFGKKEMNEQWLRGRFGR
ELEQWLRGRFGRETEQWLRGRFGRETEQWLRGRFGRELEQWLRGRFGRDAEQWLRGRFGRESGKEEELAEQW
LRGRFGRELEQWLRGRFGRDTSQWLRGRFGREHKTGGNENSNGAKEARTAVKSSEISAKDQRRDSNQVEAELSS
QVSLENENKL

>AalLWamide

MQFVATRIYFLLAGTFLLSTLVLSVPHQRENVAKRSLETSMNKDDSSSNHRGVQTDEYREDTRNKRAPRKPFILWG
RDVSAKDALKGNDKVEELQPGMWGKRAMVRPKLNLLWGRALKSKDPKMGLWGREKGKMGNEPQAGLWGRT
SEPGKVGLWGRQLNDEWRGGPGKDADAVDWLWGRGKESTRSTEVKEELTNTQELERELENKIIDLFRQLKMTEQ
KREFLEERKPKDAIGIWG

>AalRAamide

MARISFVLIFFLGTTYVPRSLAVGGCYDEGDTTTENFKRSGCGNLKTDKTFDDLDTMSEQPRGGKRDEVYEGYRPR
AGREQNQFQIDRPRAGREGVDRPRAGREFMARPRAGREYTIKILRSEFEGYGRPRAGRESLGRPRAGRESEERPRA
GRESNERPRAGRESVDRPRAGREYMGRPRAGREYVDRPRAGREFLARPRAGRESEWRPRAGRDIVLVLNDKDDE
AKGKRGFYLRKHEGGEENRERNSLNGGNVGTGLIDEREMSEARIIARGSTIDDENSKSVFKSNQPRGGKRSISGDN
SVFSLLDEDKRDNYRKSSNAEKRYVNGLTSKDGGSKDDMNNQPRGGKRSIQNDNIKGARPRAGREDASKSKETS
MKRGFDFPIDEDGQKLVFLMANKRESKRNDITSELEKLAGSLRRKRDTN

>AalFRamide_

MQRTKFAYLRFLCFLLTALYTTINGAVIDPAKKESGTEDSWSSIDSPQPSLPSSEQVSCQGQLCVYGNRQVTRRKLLK
RKTTCGPDGCFMAPKSNNFDQELDSAELDSYGIQYNDLKRRAPCEGQMCWFRGKRGKLTTKEVRERLQAQLSFL
QNLNKRQEGSKSRRTTKDSGCTGQMCWFRGKKEFDQSRNDAQNDSPCVGQMCWFRGKRTSIMKQRKPCVGQ
MCWFRGKKQILSGEERSRVEKRCTGQMCWFRGKRQLNSKDACKGQMCWFRGKRQMEENVNMDLE

>TcyVWamide

MKVPSNKIGFVVLLMQLIVSLHVTCATDMNEDSAISPVKRARSEEELQEDAVVKALERGIFARRDVISQPPGVWGR
SSERSFRARAFNRDRRLFRPQSPAERRALQDSIEHLLQAKEALDVLNKRASQPPGVWGKRAAAQPPGVWGKRTV
RTQPPGVWGKRTVRTQPPGVWGKRALEFMEREYALGQVESKDEDTIAVRSASVSQPPGVWGRRSIERSHSQEQ

>TcyRFamide

MELNAKAFIVLAFLAITFVCVTAAENNLAQQSRRQEEDDSQPFLAEEDDLVDERSVNKAKRIIEQWLRGRFGKRGQ
FLRGRFGREINSEDSFKENKARETMVQWLRGRFGREASDQWLRGRFGREATAQWLRGRFGREAMDQWLRGRF
GRDVADQWLRGRFGREVDGQWLRGRFGREVATQWLRGRFGREAAEQWLRGRFGRQLEEDIEDELFDRELDQ
WLRGRFGRETEQWLRGRFGKRESNEQWLRGRFGREMDQWLRGRFGREAEQWLRGRFGRETEQWLRGRFGRE
ADQWLRGRFGRELEQWLRGRFGREAEQWLRGRFGRESDKSEDAVEQWLRGRFGRELEQGGFEGENSLGSAMD
SNVARAVKTEGSSAAVGVKDQRQDSSKVEAELSAQGSISEKRNL

>TcyLWamide

MWTSYYLKLFLGLILMLAIVRAAPREDTEEEEVSDNKRDAFAKGQMGHKANTFRETMIRGNPKGPSILWGRNVG
AKDNLKNMEITEELQPGMWGKRSLVQPRLNMLWGRAMKEESPRLGLWGREKEREMLERPKVGLWGRSSKPGK
VGLWGRQMQGHEYQLPDRPIEGLWGKEIRARGVEDNKGRSDELERELENKIISLFHQLKSAKQRREATDKGKPGT
VGLWGK

>TcyRAamide

MDGFYVFLLLVIGTCFFCPSFTKGECNTDSTDYAFKRSDCLEELLGLEPSEFADFLDEQPRSGKRELSAQDYRPRAGR
EGDQDYRPRAGRQVLTRPRGGREYSERPRAGREYTIKIISSESQSYGRPRAGRESAARPRAGRENLERPRAGRESLV
RPRAGREDIERPRAGREDLERPRAGREDLERPRAGRQMVGRPRAGREFFERPRAGRNVVLILNDDDNKDKNKRSF
YIRSKLHAESQHDVSGSHEEEKGGELLDERESNHFVDAMDEKAATFINENQPRSGKRDLTGKTALALRLKREGEAIR
SAMGRISGYNYGFSGYDDEMNNQPRSGKRSIRMGKHSIGRPRAGREDSSEMKRGLNFPVEEDGQKLVFLMANK
RENRKNDFSKELEKLAGNLSKKRREAA

>TcyRY-precursor

MTKLLLCSVFLAIFICSVEAKAWKREGGYHSAKSHEDRQWPRYKKSELIYEDQWPRYKKSQERNSPQWPRYKKSET
YKSLIWPRYKKSEMFISDQWPRYKKSEVKHSPQWPRYKKSESSDPSQWPRYKKSELRKSPQWPRYKKEASKSAQW
PRYKKSEASKLAQWPRYKKSEASKSVQWPRYKKSEAESETQWPRYKK

>TcyFRamide

MSKEFLILQRILWLTLIALAFTWKVAAVSAKTNTKSEIYENWATNQETENQDPVSQDVSCEGELCLYGNRQLARRK
VLKGKTVCGQGGCVFDPQAKAIDQELDAAEMDTYGVQYNHLPRDSSCKGQMCWFRGRREKLSASDIQERLLAQL
SFLRELNRREMRVSNEKKAEDEEGCTGQMCWFRGKKEYEGKEGKSSCVGQMCWFRGKRGMNKKDSKSCTGQ
MCWFRGKRNLHTKKSGLKKSLGKKKRCTGQMCWFRGGRSSDPKEVDELKERQRGKRTQKENMCTGQMCWFR
GRKELQAQQMGCJGQMCWFRGKRNAKKESKN

>Tcy_Isoform_33490

MASHKMVSLVFLVVLGLVLLGSQSEAHLRYGKELTAREVIDRLTTDELDALIARLQEAKRKREVAQKREQEMAEVEL
LKQLLQKARKQK

>Alatina_alata_comp58775_c0_seq1_transcribed_RNA_sequence

MASQKMASLVVLVMLGLILLGSQSEAHLRYGKEISTRDILDKLTSDELDALIARLQEVKRKREMAKKREMEMSEVEL
LTQLLQKARKQK

>gi|1529711544|gb|PEDN01001281.1|_selection_translation_frame_+3

MAKIINVALCALLISQVLCEVIETKKTKDPDEEEDPEQLEDAKRSIKQWLRGRFGKREDNQWLRGRFGKREDEQWL
RGRFGKREDEQWLRGRFGKREDEQWLRGRFGKREDGQWLRGRFGKREDEQWLRGRFGKREDGQWLRGRFGK
REDGQWLRGRFGKREDEQWLRGRFGREGSQWLRGRFGKEAEQWLRGRFGREAGQWLRGRFGREAGQWLRG
RFGKEAGQWLRGRFGKEAEDEEEGLEDEFEQWLRGRFGKEVDEQWLRGRFGREALQWLRGRFGRDLAEQWLR
GRFGKEAEEQWLRGRFGKETDEEDQWLRGRFGKEAAEQWLRGRFGKEAEDQWLRGRFGKEAEEADQWLRGRF
GKEAEEQWLRGRFGREAFEQWLRGRFGRDADEQWLRGRFGKEAEQWLRGRFGREGEQWLRGRFGRDNTSSS
DEKDAKKDEAKANEKKGIEKAEKSKK

>Fgenesh_Nemopilema_nomurai_isolate_NNO-Tongyong01_scaffold18_contig1

MAFRTTLVLLIAMLLFTAEVASVATYLQGESSGGQQQHRPHSPGPSESKYPIMSSFFDKPQTQEGVGRQGSDKLN
GLTNPNGMGGFTSHEKPSSDYFLGKASSDYVQGNFYDGWARDIKTMNDGNEIQRVQLTNLDADASARPKRRLQ
PLRYNDDLSWSSRYGLDTESQSSNPTIARVVTANNEQETSMDESGAKMEDIETNQLYRRNAKVDLNPPFWSGRY
GRAALDSNAEYSDSPPLWIGRFGKEAGATDDRQLVGKEAQKGNSQPLWTGRYGREVKDVNSQPLWSARFGREV
KDVNSQPLWSARFGREAVEGNDQPLWSARFGREVKDVNSQPLWSARFGREVKDVNSQPLWSARFGREVKDVN
SQPLWSARFGREAVEEVKDVNSQPLWSARFGREVKDVNSQPLWSARFGREVKDVNSQPLWAHGLEERSKMLTV
NLCGAHGLEERSKMSTVNPCGAHGLEEVKDVNSQPLWSARFGREAVEGNDQPLWSARFGREVKDVNSQPLWS
ARFGRRSKMLTVNLCGAHGLEERSKMLTVSLCGAHGLEERSKMSTVNPCGAHGLEERSKMLTVNLCGAHGLEERS
KMSTVNPCGAHGLEERLWKVMTSPCGAHGLEERWKVTVNPCGAHGLEERSKMLTVNLVERTVGREVKDVNSQP
LWSARFGREVKDVNSQPLWSARFGREAVEGNDQPLWSARFGREAVEGNSQPLWSARFGREVKDVNSQPLWSA
RFGREVKDVNSQPLWSARFGREVKDVNSQPLWSARFGREVKDVNSQPLWSARFGREVKDVNSQPLWSARFGRE
VKDVNSQPLWSARFGREVKDVNSQPLWSARFGREVKDVNSQPLWSARFGREAVEGNDQPLWKARFGREVTNV
NSQPLWTGRYGREAYINADQKRVKKEAEEVNSQPLWSARFGKETNAIDEEGDPGKLVMHKRKKLTNGLEAEDTV
QRPLWASRYGRDTTQKRKVRAGRRGTRVKDVTGRLIHRSSKGDTEKQEFQEDTDEDGGRGVGSEEEETPQHLSG
ERFADDATGTRLLSSIERLRIALHRVSGSNEMKLGPLIKLLRGKFEVAEQKSKRTSARRRGKKLTWRKATSDELKRFD
DILHSKIGLRESDTRQIQSKRRNAEAKTRAIKTDF

>Nemopilema_nomurai_isolate_NNO-
Tongyong01_scaffold11_contig2,_whole_genome_shotgun_sequence

MMSKTFPTTLILLMVGQFVGFCACKAVVNPGIFLLEADVPQTGQMPEGIGNKASYSELWEASVGSQDEPEEEGIP
HLRMGKETAPHMRYGKDFVDFLLHGAKNDAAPHLRYGKDVDENVRYGREVEVNIGYRGEMPPQLRYGKEVKRQ
LRYGKEADQLRYGKEATQHVRYGKEAQHLRYGKEAQNLRYGKEAENVRYGKEAQHLRYGKEASNVRYGKEAANV
RYGKEVDNVRYGKEAANVRYGKEVDNVRYGKEAANVRYGKEAANVRYGREAANVRYGKEVDNVRYGKEASNVR

YGKEAANVRYGKEAQHLRYGKEAQHLRYGKETQHLRYGKEESIQHLRYGKEVGENSEQGKQGPVDAKLLIGGHYE
AARETDENGADVENAIWGTVIHSKTGEDILNVLKNLKALKQKPVSDCLTHLTTSIR

>Nemopilema_nomurai_isolate_NNO-
Tongyong01_scaffold106_contig1,_whole_genome_shotgun_sequence

MELLWVLQLFIACYAQYVCCGRTATVAEDLALLSDQVTSQQEESTSELLLDLVKQLEKYCGTSSNLDQNVCRNALH
YLLSNFENFDSLMEEEKKGLDGAVSEDSLPRSGKREVEEEEMLEGMLPRSGKREMDLPRSGKRMLEMEKELPRHG
KRDFVLPRSGKREMDLPRSGKRMLEMEEELPRHGKRNFRLPRSENREVDMLEVDEIMGRQEEERDVYLPRSGKR
GHMVRLLNQQDRSRFEDLAMPRYGKKELPRAGRRVAARPGRREMSLPRSGKREINLPRSGKRSLKRSEAREENES
EKDLAEMSESTKRSIALPRSGREFSMLPRSGKRASATQGELVARRESYFPRSGREQTGQAEESFELSREMGMLRPG
KRNTARMLRPGKRNSVEALRPGKRNSVEALRPGKRNTVEALRPGKRNTVEALRPGKRDSFEILRPGKRNTVEALRP
GKRNSVVILRPGKRNSVEILRPGKREEVIETETEDELRSKAFATS

>Nemopilema_nomurai_isolate_NNO-Tongyong01_scaffold33_contig28

MKLITLGVVLTFSISTLLVAKAEDVDDASLEPAPPGVHGLLTREGDDFEEDENDALEHERRENQPPGVWGKRDSQP
PGVWGKRSKQPPGVWGKRTSKEENRLHRKKENQPPGIWRRGENQPPGIWRRGENQPPGIWRREANQPPGIW
RRGENQPPGIWRRGENQPPGIWRREANQPPGIWRREANQPPGIWRKRENQPPGVWGKRSKGGHHATIGSEIK
GRAQIKVAKKGLNEREAALIRTVKRLRDALAKE_

>GHAR01037764.1_TSA:_Nemopilema_nomurai_nem_comp53040_c0_seq1,_transcribed_RNA_seq
uence

MLLLLASIVVFDWTVHAAVHKSREVTKGNQMAERDYRRRESEYLETEMQRDEGYTKVKRGEFGKRSNADLREEQ
PGIWGKRSVPRVVGFNTGPGIWGKRAQHGPFGNPRIKEAWGKKNEPGMWGRRAALTSDRQQMLSMGRYLQK
QLGITRRKQWDVAALLNILKLLQSMQGERQAIGEVEQDEEEKRSIGPWLGMDRRIAKGHHVPELLIHLIGGSDD

>Nemopilema_nomurai_nem_comp60006_c0_seq2,_transcribed_RNA_sequence

MKLQLIAICCFMLAFRASSSLEEQRSSVDDAEDYDQEHLEERQARDVNAEDMHVPEKRNIRDQLLKGPHLLLGRM
DAGADDTAVWESTRNAADGAEKRTVADMQRRSALDDVKRWAREVAQKISREAEQHGTAGLWGKREAKHVLSE
KDLVVLLEALNNEEGQTNARRGIASTQGEYRKQPDDLGLWGKRATRRSSQYSQEKRRRSQTKRQGQPNNIGLWK
GKRGFHGNENSKKPDGDSKIGLWGREQYGKAGMWGRKRSHQIDSA

>GHAR01038137.1_TSA:_Nemopilema_nomurai_nem_comp53321_c0_seq1,_transcribed_RNA_seq
uence

MDVVWLMLFFITVTSTTYGRSTGKRATIDAEANELTIDERRQPFENDLDIDIYKAAFRSGKRECTSPMCWFRPGKR
NSRFNVETEKSKKRASSRVEESCNSPMCWFRGGKKRAVDFAEIAKRLLHRKPALKRFLQNVMHRQRKTQRQVSED
NCDSPMCWFRPGREVIENAKKGKAKMSRGNWKSFFNKKIQRRIDTSMRGKSVMDENHLTNRKWKATRDIGAG
DSSSLLWEKDNAAY

>Rhopilema_esculentum_Unigene0032526_transcribed_RNA_sequence

MAKILSTVLCALLIYQVLCEKIEKKETKDNEKVGDKEQLDDAKRSIKQWLRGRFGKREDEQWLRGRFGKREDEQWL
RGRFGKREDEQWLRGRFGREDEQWLRGRFGKREDEQWLRGRFGKREDEQWLRGRFGKREDEQWLRGRFGRE
DEQWLRGRFGKREDEQWLRGRFGKREDEQWLRGRFGKREDEQWLRGRFGKREDEQWLRGRFGKREGEQWLR
GRFGKREDEQWLRGRFGRGAEQWLRGRFGREAGQWLRGRFGREAEQWLRGRFGKEAEDEDDGLEDEFEQWL
RGRFGKEVDEQWLRGRFGREALQWLRGRFGRELAEQWLRGRFGKEAEEQWLRGRFGKETDEADQWLRGRFGK
EAEEQWLRGRFGKEAAEQWLRGRFGKEAEEQWLRGRFGKEAEEQWLRGRFGRDALEQWLRGRFGRDSEEQWL
RGRFGKEAEQWLRGRFGRDFADQWLRGRFGRGSDSSNDEKDAKTDVAKESERKGGEKDLGSADKSK

>Rhopilema_esculentum_c12255_g1_i1_transcribed_RNA_sequence

MVRLCAMFLPLLTEILFTVNTASKLTRTGVYSYEVPQKDHPHKANSRHLSVLEYPQQLTSSNQHDDVEDLSSQNGK
NLKDENGFRNFRIRSFEHLQRKRSLSKNRSGKEKIIKRREGTRGVQIKNFFIDPYGELDQKKGMTYQKKNMLWNLR
YGRETFSKMYQPLWNGRYGRKTYPTDEQWRQGRDTEEVNGQLRQRRETGE

>Rhopilema_esculentum_Unigene0033256_transcribed_RNA_sequence

QPLWSSRFGREANAVNEQLRPGREAEEANSQPLWSSRFGREANAVDEQLRPGREAEEANSQPLWNSRFGREAN
VVNGQLRQRRETGEVSSQPLWSSRFGREANAVNEQLRPGREAEEANSQPLWSSRFAGREANVLNGQLRQGRE

>GEMS01049336.1_TSA:_Rhopilema_esculentum_c56832_g1_i1_transcribed_RNA_

MTKSFPACLVFLIATEYIGLCCCKALVNPGVFLLEADMPFKEQISQEIETKPDPSNMWKPSEESDEEGYENGIPHLRY
GKEAAPHLRYGKELENFFHYGEESDDAQHVRYGKEADQHVRYGKEADDHVRYGKELQHLRYGKEAQHVRYGKEA
QHVRYGREAVQHVRYGKEVKKQLRYGKEAKHVRYGREVDQHVRYGKEAQHK

>GEMS01057357.1_TSA:_Rhopilema_esculentum_c62341_g1_i1_transcribed_RNA_sequence

MKLILVSLLSFSLYAQFVSCRRKISVAEDLALLNDQIISQQQKSTSELLLELVKQLEKYCGTSSDLDRNVCRNALHYLLS
NFDNFDSSMQEEKKNARFSAVASEDALPRSGKREEVSLPRSGKRENIDLPRSGKRELALPRSGKRLVELRRKIQRYG
KRESRLARSGKREVDMFEVGEKKRNVHLPRSGKREMETRASGRQEISRYRKRNFAMPRYGRRELELPRAGKRKTE
MLWFEKRERRLPRSGKREFDLPRSGKRESAMARSVTEGERSAEDVTERSSEKTVASFVKVDDESMKRSMTLPRSG
REYIMLPRSGKRAFVASERELPRSGKRRAVLAAGEENYLPRSGREEAMHAKRNLELSGELELPRSGKRQLVEGKQV
DGADPEEELEDNAFMAS

>GEMS01058479.1_TSA:_Rhopilema_esculentum_c63002_g1_i1_transcribed_RNA_sequence

MRLITLGIVVLLSMSNCLLTTAEDSNDASLEPVPPGPRSLFTKEGDDFEEDENDFLEHERRENQPPGVWGKRENQP
PGVWGKRSNQPPGVWGKRVSKEENQPPGVWRKKENQPPGVWRKKENQPPGVWRRDNNQPPGVWRKKESQ
PPGVWRKRENQPPGVWGKRSNKKHEENFSGVIDGRAMKEIEKDARGLNERKIALIRTVKRLREALAKEYK

>GEMS01050632.1_TSA:_Rhopilema_esculentum_c57817_g1_i1_transcribed_RNA_sequence

MMMQLQLVAICCFALIICKSLSIEVQRSLNDLDEDARAMHREKRLVQELNAEDLSDNEKRNLQDQKVKRPHLLLGR
MDAGIDESDLWNNNHDAGYDSFKKTAIDTQRRSALDDVKLWAREVAEKISREAEQHGTAGLWGKRGAKPTLSEK
DLALFLNALNNEERKANARRTIADTEIENRQQPEDLGLWGKRSTKSKRTSRHSDEENRGNHLKRQGQPGNIGLWK
GKRGFHGNGEASKPNGDSNVGLWGREQGMWERKRSNRLATT

>GEMS01035705.1_TSA:_Rhopilema_esculentum_c44618_g1_i1_transcribed_RNA_sequence

MGVIKLALVFVTLVSFANGRSTEKRTEIDAEANELTIDQRREPFENDLDIDIYKAAIRSGKRDCTSPMCWFRPGKRG
MLPRFTLGSSKRKSNDRNGESCNSPMCWFRAGKKRAFDFGEIAKRLLLRKPALKRFLHNVMHRHKKLQRQVSED
NCDSPMCWFRPGRQVNDEKKKKEKRVINMNSGERYFKTQTQKRAGLTATGKKETSTKLSVGRGRNVDTHGAAE
DSDYQTWKKEYVVD

>AauRFamide

MNLTLQIVLCAVFMTYALCESTEKKETPGAQSENEKLGPAKRALEQWLRGRFGRESGKEPRELDQWLRGRFGKRE
DSQWLRGRFGREAKQWLRGRFGKEADENEEALESEFEQWLRGRFGKEVDEQWLRGRFGREALQWLRGRFGREL
AEQWLRGRFGKEAEEQWLRGRFGKESEDEAEESQWLRGRFGREAEQWLRGRFGKEAADQWLRGRFGKEAADQ
WLRGRFGREADKQWLRGRFGKEVNGQWLRGRFGREANGQWLRGRFGREADKQWLRGRFGKEVDEQWLRGR
FGREAEQWLRGRFGREIEQWLRGRFGREESHEKMTRELEQWLRGRFGRDAADQWLRGRFGRGEKTSEALPKRT
DAKDSKTNEKKSDDRFESELNSAVKAAKSS

>AauRYamideI

MHWNARSCLLVCILNLARTDLLNVLASNHVHQGGVGNDEHIHRQGAGNFEPVYSDVGTRDTREDLNDKQEVRH SADHSDTDSDEGGGPVIEPEEPVEEHRHRRSFINPGLLFSRYGRVAKAQGVEALPLHRKRMEYNARQRRELYADEV STAGYEDVRDRMEHKRSTSNKFGKEAHEMKMPSSATRKRRNVVYSNNQHRTGARYDSEAENEKPFWKVRFGRE ETIGPLWSARFGRELRDKGPLWKSRYGREGREVAPPWASRYGRDAQEKSLIMSSREAHDKSPFWNGRYGREEGH ETPFWKGRYGREEGHETPFWNGRYGRESANEKPPWAYRIGRDVEESKSLVREAAPHMWKSRFGRDSKDRREQV AKQKRMEAAINMELMAEPEGGHEQYLARHGKNGEEQELENEDTQPSVRFGRAVVSADAVDVDRDGSRMKRG WKGRYGRVNPRIVNHIVVPQRSHGSGKEFMNYRPIMYAEQNVDGEVNTQRYDSKMDNWPARSKSQGVADLER QGGAGSERADAERDKTLEENDLAGTGFDGVDGVASTNDRQAEDRYGNRFLFQMERLRQKIRDTEMKEGLAATG EGTGFSRGKSDRGTSAGRPRSRNLNWEMENAND

>GBRG01040571.1|_translation_frame_+3

MTQNALFLAIAVLCSNYLYQCGCRSIQDTDVFLLDADNPLRDDEQDSFDDIDLRELAPHLRYGKEVAPHVRYGKEV APHVRYGKEVAPHVRYGKEATQIDDIDRHQRYPARMPGKDFAVHGKLLSDLASGTQVDDKAEHGRRMSALMKIL RDVASESTKLHRKRAVKTNA

>AauRSamide

MRALKIMLAVVALVFLSVQYAYSLNAELLADELAMVDAEETGQAYKSTSQLLLKLVEQLEKYCGTSSESDRDLCQNA LRYLLGNFNDFDELMNEANAGGKDQARNIINKEGRVRSESGDDEAASMVLVSEREHMGMPRSGKRESEYPRSGK RDTEMPRSGKRGIEMSRSGKRDFEMPRSGKRGSDKMAENRELGTSILAEDRSGYPRSGKRELELATARDRIGMPR SGKREADDYPRSGKREADVTQSEMQMNDRLRAGRNMQRSGKREADYPRSSKREADYPRSGKREADYPRSGKRE ADYPRSGKRDAEMPRSGKRNIEMPRSGKRGMEMPRSGKRGIDMPRYGKRGIE

>AauRAamide

MEAMKIMLAVVVLACLSVQCADSLNAELLADELEVVDAEETNQGYKSTSELLLTLVEQLEKYCGTSSESERNLCQNA LRYLLGNFNDFDELMKEANAGGKDRASAIISEGGQGERGDEELASMLLVSERERMGMPRSGKRENKYPRSGKRDI ALPRSGKRESDEMEEKRESDDLLFAEDISGYPRSGKKELKLAAAMDRMSMPRSGRRAADDYPRSGRGEVDMMNS AMQMNDRPRAGKRVVERPRAGKREFERPRAGKRELDRPRAGKREIDRPRAGKRDLGRPRAGKRELDRPRAGKRE INRPRAGKRELDLERPRAGKRELDRPRAGKRELDRPRAGKRELDIERPRAGKREFEILNTGSEENDFPRSGKRDIDER DIETPRSGKRELDYPRSGTGRK

>AauTW-precursor

MQVLILVFISTLCLSSSVRGDDDKEAQWRPMPPGALGDDRLTSNHEKIKNDALGDGRKKSKGKSDSLIEDSLNEGA FDDEELSAQGKDEGISARENQPPGTWRRREIQPPGVWGKRSNQPPGTWRKRGSQPPGTWRKKENQPPGTWRK KENQPPGTWRKRENQPPGTWGKRSN

>AauLWamide

MERLTVLYLMAFTFICCSAKDMPQATMTKYDVDDSLVPDFEGGLLPEDDETELENLEQKNAAQHAKSRLKGLDEI HHIIREIVEAPKNEQPGNAGLWGKEDRLSSSQEDKQKRLLEEYTKLAMGAAKTKAREVAEKAIRAALSKREQRGSA GLWGKRSVAVHNDVPEKVWQELLHDLTDEELSAVARRVAAEGGHRLVAKASSMREPKSKKQAVRPVLSARSAEQ PDDPGLWGKRSARHRAMKGTGVVNKITHWEAESERESSKHRIDQPTNIGLWKGKRASSGKGQLKKPNGGSEAG LWGKREVRQQHGNNAGMWGRKRENPFVEE

>AauMCWFR

MHLRRTIFIVVFAFSVIKAAPLENRKKRCQSRNCNSETEEIHLDHIRNLSEDAIDIDLYEAFTTAVKRGCSSPMCWFRP DGKRGMMLKRGLTRREKRKNHKRSSQSLKRVERKREEMVKQKKNCASPMCWFRATGKRREMMSQGQKELFVK RMALKRILKGMRDKRKREQEGETKTGCSSPMCWFRAGREMDNNSDKILQPDNEETKKQGYSEKHRRRDPGEAA AKQIRMMKDAAERMQKKFVEEGKMFGPRDASMFGARGGRVLGAGEANVFRDEDDNDHLELSAA

>Aau_RPamide

MIKTSFIFLRLICLVELNSVTAEDSNVNIIKNELKRTNLKTNGKLRLILATKFSEEPIEHRGVKSKHHRRSDEKRHNDDG
RRAFWWSPGTRPGREVKSKHPRRKDEKRHIDGRRAFWWSTGTRPGREVKSKHHRRSDEKRHIDGRRAFWWSP
GTRPGREVKSKHPRRKDEKRHIDGRRAFWWSTGTRPGREVKSKHPRRKDEKRHIDGRRASWWSPGTKPGREVR
PKDQRRNDKKRHLERPGREEKSKHHRRSDEKRHIDGRRAFWWSPGTRPGREVKSKHPRRKDEKRHIDGRRAFW
WSTGTRPGREVKSKHPRRKDEKRHIDGRRASWWSPGTKPGREVRPKDQRRNDKKRHLEVGKGAFWWPIGTKP
GREIDMKNQKRCDDEDLATSKEIHLLAHTEENH

>HAHC01044519.1_TSA:_Calvadosia_cruxmelitensis,_contig_Ccrux.17646.c0_g1_i1,_transcribed_RN
A_sequence

MNLLQYSAAILLLVSLAHCMPGHSKRDHVIEEIDEIDEVDDREMHSTHDENERRENHHDDEEKRGAHEPRATIEHT
RGLDERREIHEHDTEHEKRNLGAREAKEQFLRGRFSREMEEQFLRGRFGREALSQFLKGRFGRESEDQFLRGRFGR
ESMEQFLKGRFGRESESQFLRGRFGREAMEQFLRGRFSRELEEQFLRGRFGREMEEQFLKGRFGREDMEQFLRGR
FGRSSDEGSSNELREYFAASRYHNGDADSQAYEDKRSLQMKK

>HAHC01040962.1_TSA:_Calvadosia_cruxmelitensis,_contig_Ccrux.16828.c0_g4_i2,_transcribed_RN
A_sequence

MVSAFTTGCKVYGLLLLLSINAACSPMRDQESTDEDDQSVWEWIQSYLNGDSYEGEAEESLRSIVEDSNQFKRMK
KASNEEIRPSADERDARNMLLASLLYDRYYEDLTENRDYSRSQENDHVTAAEYMNSLRERLVSNGLKAGKRDDDM
WLLTNRLHGAFRPRSGKRSEEEDRPRSGKRAEKDRPRSGKREDEDRPRSGKREDEVRPRSGKREYEDRPRSGKREN
EDRPRSGKREDEDRPRSGKREDEVRPRSGKREYEIRPRSGKREDEARPRSGKREDEDRPR

>Ccr_AWamide

MHVILSLLLVFIVNHMTEAGSHHHDNLGPMPPAIRKEIDEIYEDMISKELNEDLPDIYDKRENQPPGAWGKRENQP
PGVWGKRENQPPGAWGKRENQPPGAWGKRENQPPGAWGKRENHQPGAWGKRENQPGAWGKRENQPPG
VWGKRENQPPGVWGR

>Ccr_GFamide

MHGIIVIATLAIFLVSCNGAARAKPAKNHGNCDADCQRIKKLKELMISRGFGKRSIVEDGRMKASQCDADCQRIKKL
KELMISRGFGKRSIVEDGRMKTSQCDADCQRIKKLKELMISRGFGKRSIVEDGRMKTSQCDADCQRIKKLKELMISR
GFGKRSLLLDDVSNESAQDWSSSMLNELLLSHGCSLHQVIRDFNKQPTAR

>HAHC01097818.1_TSA:_Calvadosia_cruxmelitensis,_contig_Ccrux.25030.c0_g1_i1,_transcribed_RN
A_sequence

MLCERSFTLILLVVLAIISSTQSVSDCVNTMSVMVPQERCVTGQVYENHMLSGGLWGKNFIYIGKTANIEQCVRLV
CKDISSGFAFLLNGTFCYSVKCLESCKVDVGRRSGNVNTMTVELQRKVFFSNKSECVSQSRRHANDVEKSGEYRILR
DVIRKANNIDVRPAFSSGD

>OFHS01000588.1_Calvadosia_cruxmelitensis_genome_assembly,_contig:_Ccrux.03.000588,_whole
_genome_shotgun_sequence

MFKIIVLSLMALMCVLPRGVTSLGLRNSEFVTTSQQSDEIGAEEDSTTRGSSEDHVIETRQINHNDPGLWGREVWY
QGNTGHRNNDRAAYLRELMRSRVLDMPLRKLIDYTSKAMQSAKGKAKKVHGHTTASGAEENVKRQVQTGMW
G

>HAHC01118832.1_TSA:_Calvadosia_cruxmelitensis,_contig_Ccrux.45647.c0_g1_i1,_transcribed_RN
A_sequence

MATMSVSLITLSAMLLLAVVVPNIMSAHLRYGKEVNKLKMIKAVERNLAERLLDVMVKEQALKDEMKLAKNDLDAEIEAEKDLKTKKFSDSRDSLYDTTDLDV

>Calvadosia_cruxmelitensis,_contig_Ccrux.03956.c0_g2_i1,_transcribed_RNA_sequence

MKGGGEKVGPGNDNAPGWSSRNAFVVVTCFAIAPSTECQGLRWRSAVYGSWCARDSIAAGL

>HAHB01030546.1_TSA:_Haliclystus_sanjuanensis,_contig_Hsan.30546,_transcribed_RNA_sequence

RENQPPGVWGKRDNQPPGVWGKRENQPPGVWGKRDNQPPGVWGKRENQPPGVWGKRDNQPPGVWGKRENQPPGVWGKRENKKENQPPGVWGKRENQPPGVWGKRENQPPGVWGKRENQPPGVWGKRENKRENQPPGVWGKRENQPPGVWGKRENKRENQPPGVWGKRENQPPGVWGR

>HAHB01040451.1_TSA:_Haliclystus_sanjuanensis,_contig_Hsan.40451,_transcribed_RNA_sequence

MKATCLLVLLCAIISIQCLPHNKRALEDHVVEEIDEIDEQDKRDAKRTSHESNTKREDDHHNEDRREQFLRGRFGREMEEQFLRGRFGREDMEQFLRGRFGRELEEQFLRGRFGREKEEQFLRGRFGREQFLRGRFGREMEGQFLRGRFGREDMAQFLRGRFGREMEEQFLRGRFGREDMEQFLRGRFGRENHGKESREFYAARIDDHDGYDADEKRSTQLK

>HAHB01061752.1_TSA:_Haliclystus_sanjuanensis,_contig_Hsan.61752,_transcribed_RNA_sequence

MKLFHVNAVFLCCTLTFTHFAFSQPIDDWLSTYDSVTDSANEEDFKRALDRPRSGKRDEIEDLNELLAAMTKNEELYNELEEEPSEEGDEFRQRSGRSKDDRPRSGKRAADVRPRSGKRSEMERPRSGKREMSRPRSGKREFSRPRSGKREALRPRSGKRSETERPRSGKRSEIERPRSGKRSETARPRSGKRETRPRSGKRSELERPRSGKRSEIERPRSGKRAESSRPRSGKRAELLNLIAEELYAMDRPRSGKRAFDSRPRSGKRDLDDNTTYNDKFNELDTAADNDMWSSMKRSTSDIFDSTTSSDTTNYDNEANNIEM

>TSA:_Haliclystus_sanjuanensis,_contig_Hsan.45894,_transcribed_RNA_sequence

MYATRWIFVAICLAVTMQLTLTFRVIGQSNRLEEISSLEEDESAMLQSRQLDDSEPGLWGRETTQKHHNGVVENENYEKTMMQLLKERATNLSIKQLVNYIAKVILDSNQSLNRRKPSKVDSTPPPKRQVRTGMWGKRGV

>TSA:_Haliclystus_sanjuanensis,_contig_Hsan.125202,_transcribed_RNA_sequence

VFAIVCCKDVQESCSDVTKVLVPRERCVAGRTHSNRLLKGGLWGKNFVYIERTDSVQKCVELVCDRIASGFAFFLNGTYCYAVKCLERCEVDYSQRSTNVNTVTIELARKVVFANETQCLRPSKQMDVKSNEMSGEYRILRDIIRQSKDVDVRPAISNRDRINMSISLRLVQILDVVSIPLPFNQLNDRSPLLTFYPAGCAP

>HAGZ01008087.1_TSA:_Craterolophus_convolvulus,_contig_Convo_TRINITY_DN5429_c0_g1_i1,_transcribed_RNA_sequence

EIDDLYEEIFGKEEDKKVSDVLDRRENQPPGVWGKRENQPPGVWGKRENQPPGVWGKRENQPPGVWGKRENQPPGVWGKRENQPPGVWGKR

>HAGZ01051122.1_TSA:_Craterolophus_convolvulus,_contig_Convo_TRINITY_DN6267_c0_g2_i1,_transcribed_RNA_sequence

MNFVVLWMAIGLMSTAAHGLPAQDKRSAAEHVIEEIDEIDETDERESHPGGEEKRHSHEPRGMVEHTRSENERREHAHHDESHEERSSHPDREAREQFLRGRFGREGMSQFLRGRFGREDEEQFLRGRFGREDMEQFLRGRFGREAMHQFLRGRFGREDEEQFLRGRFGRELEAQFLRGRFGREAMHELLRETYGHNHDEEGKELREFVSARYDSGEADSRHYDDKRSLHGERK

>HAGZ01021818.1_TSA:_Craterolophus_convolvulus,_contig_Convo_TRINITY_DN2329_c0_g1_i1,_transcribed_RNA_sequence

MIIVLFVLPTLLTCAVIRDANGLDASLSSDNDHWDGIKTCLENALDDESTEEGPDAYNGEETWEGEDSEKKAAFTWLMSKIMGEDGDESFEGEDEDEEIRFSENEGIFRAGDDRPRSGKRYTEEDRPRSGKREAENRPRSGKREAEDRPRSGKREDRPRSGKREAENRPRSGKREAEDRPRSGKREDRPRSGKRE

>HAGZ01012697.1_TSA:_Craterolophus_convolvulus,_contig_Convo_TRINITY_DN2619_c0_g1_i1,_transcribed_RNA_sequence

MRNGVVIVFTLAAVSTALPAPEPGRCRAGLWCHGKRNELQDEGTQLTETQNKRDQMETALDSEEKRELGRCRAGLWCHGKKKREYLSKRLAHFATYDEMGRCRAGLWCHGRKREEIEAPKAVRSPGRCRAGLWCHGKRDEGTDVGGEIEVEGSSTDNIGATNIKF

>Craterolophus_convolvulus,_contig_Convo_TRINITY_DN26583_c0_g1_i1,_transcribed_RNA_sequence_

MASAKILVALLVVALFASVFVSETEAFLRNGRDSIPFKRRQTQRFYYPEDLEEFQRPQENKLDTEYLKQK

>HAHD01030305.1_TSA:_Lucernaria_quadricornis,_contig_TRINITY_DN14230_c0_g4_i1,_transcribed_RNA_sequence

MSNCLLASLALVFLSHYIEAYEHAEDLSRPLPPSIRKEIDDLYDEVMRRTEESHYSAGEKRESQPGRSGKRENQLPTGTWGKREKIPTGVWGKRENQPPKGTWGKRQDIPTGVWGKRENQPPKGTWGKRQDIP

>HAHD01021334.1_TSA:_Lucernaria_quadricornis,_contig_TRINITY_DN15109_c1_g3_i1,_transcribed_RNA_sequence

MSDHVIEVDEIDEDIDRETERKEATDEPRAATEDERSMHEKRHTSDSERTTREAAEQFLRGRFGREAVAQFLRGRFGRETEEQFLRGRFGREAVAQFLRGRFGRETEEQ

>HAHD01049480.1_TSA:_Lucernaria_quadricornis,_contig_TRINITY_DN9917_c0_g1_i1,_transcribed_RNA_sequence

MHRSVVAVVALVLLASSNGLPTRDADQGEGVGAMLKWERLYNYINSDTYSREDNSFTDYLRKLFPGNDDDEETDEAGNEERRDAKKTSLSYRDYVAELSRSKTMQDNRDAAEELRECTDEGYSTQDDAEKRNINAVELSYRPRSGKRKAEEERPRSGKRSEDDRPRSGKRSEDEKPRSGKRSEDDKPRSGKRSEDEKPRSGKRSEDDKPRSGKRSEDDKPRSGKRSEDDKPRSGKRSED

>TSA:_Lucernaria_quadricornis,_contig_TRINITY_DN3242_c0_g2_i1,_transcribed_RNA_sequence

MDGHIACILCVLLAAVQSVAGAQDCDKSAVIMMPQERCLTGKIHTNHMLKGGLWKKNFVYLGQTANIAQCVQMVCSDITTGFAFLLNGSFCYSITCLEKCEVDTRPRGSGKVST

>TSA:_Lucernaria_quadricornis,_contig_TRINITY_DN1885_c0_g3_i1,_transcribed_RNA_sequence

MNNLTSSVVLACLIVIAVQATTAYRIAPLRAEDSTASKREVASASLDMDDTSSDTEIDARQIDVNEAGLWGRETASSLRPSQQHTRQSLNYLMAKTRVEHMITRIADAIVRLAGEQSQLQTREANEEPATESQKRQVKTGMWG

>HAHA01038762.1_TSA:_Haliclystus_auricula,_contig_TRINITY_DN7857_c0_g2_i1,_transcribed_RNA_sequence

QPPGVWGKMENQPPGVWGKRENQPPGVWGKRENQPPGVWGKRENQPPGVWGKRENQPPGVWGKRENKRENQPPGVWGKRENQPPGVWGKRENQPPGVWGR

>HAHA01071884.1_TSA:_Haliclystus_auricula,_contig_TRINITY_DN13894_c0_g1_i2,_transcribed_RNA_sequence

RGRFGREMEEQFLRGRFGREDMEQFLRGRFGRELEGQFLRGRFGRESEDQFLRGRFGREQFLRGRFGRETEDQFLRGRFGREDMAQFLRGRFGREVEEQFLRGRFGREDMEQFLRGRFGRENHGKESREFYAARIDDHDDYDTNEKRSTP

>HAHA01075613.1_TSA:_Haliclystus_auricula,_contig_TRINITY_DN11232_c0_g1_i3,_transcribed_RNA_sequence

MKSFYVNAVCLCCILTFSHYAFSQPIDDWLSNYDSATDSVDEEEFKRTVARPRSGKRDEIEDLNDLLAAMNKNEELYKELEEESVTEREAFRPRSGKRSKEGRPRSGKRDVEDRPRSGKRSELERPRSGKRETLRPRSGKRETLRPRSGKRSEAERPRSGKRSEIERPRSGKRTEMDRPRSGKRETRPRSGKRSSFERPRSGKRSETERPRSGKRVEHSRPRSGKRADLLNLIAEEINSMDRPRSGKRDVTDDINTNDDVNEVYTVDNDMWKSMKR

>TSA:_Haliclystus_auricula,_contig_TRINITY_DN6089_c0_g1_i1,_transcribed_RNA_sequence

MYATQWIFVAVFLAATTQLALTFRVKGQFNHPEEISSLQEDESPMIQTRQLDDSEPGLWGRETSPENHNRAVENENYEKDMMNIVKERASNLSVKQLLHYIAKVILDSNRGSDNRNPKLVSTPPPKRQVRTGMWGKRVV

>TSA:_Haliclystus_auricula,_contig_TRINITY_DN36533_c0_g1_i1,_transcribed_RNA_sequence

CQEAQESCSDVTKVLVPRERCAAGRTHSNRLLKGGLWGKNFVYIERTDSVQSCVELVCDRIASGFAFFLNGTYCYAVKCLERCEVDYSQRSIN

>ALY05321.1 peptide precursor 2 [Clytia hemisphaerica]

MKIYFGCLFVILSVNQIGCYPSSNQNSERELVRRIYKTVHPNPHYQVNEIQRVKEALKRRVLENVHRVDS

LKASLKRVLGQDAGNGFHLSSSKIFQKKRSKARLPHSYMFRKRQNSPGALGLWGREVEAPGDIGPPGIWG

DVVPDETRKDKPGAVQGLWGKDERVIRALLKTLKR

>ALY05320.1 peptide precursor 5, partial [Clytia hemisphaerica]

MELKYFLASSIFVIIAIQLASCSSKAEEYKQMKKEVDGLLKEIVSQENAKQHTSEKKSSQWLNGRFGKRQ

LVSGRFGRELKQWLNGRFGREATEQWLNGRFGKREADQWLNGRFGREVEQWLNGRFGRDAAEQWLNGRFG

KRSANQWLNGRFGKRSADQWLNGRFGKRSADQWLNGRFGREASADQWLNGRFGREAADQWLNGRFGREAK

EQWLNGRFGREVEQWLNGRFGREAGQWLNGRFGREMGQWLNGRFGREA

>ARN59429.1 pp11 precursor [Clytia hemisphaerica]

MDQSLSSILLLLCCWVALTTCMSVQRKEAGDALSALDKENAKKSANSITEELARNLMEHLYDEIRKRSNS

NEETISNFRRASSDTHRQQQAPKGLWGRELQPGNPPGLWGREASEAENTDSNDGPIPGMWGRREADDKNA

HEKFQ

>ARN59428.1 pp9 precursor [Clytia hemisphaerica]

MYLSVGFLLFLCHQFQDTHGLSIRGPNDAQQLINSHGENDLPSGGMWETAKSQAMETYRKDSRRGIPKRG

RSSFLAIGKKDDSLSLGSKMKKSLESPSLSVWRRGDSLHSILRVNPSLGIWKRGDSLMRRFGRKNFGKDN

SRRSLVREIENDAFGMNRKDEFPSPGMWEKAKSQYFGKREFRNSILGKHGKDEFPSPAMWEKAKNQYFGK

RDIRNSILGKHGKDEFPSPAMWEKAKNQYFGKGR

>ARN59427.1 pp8 precursor [Clytia hemisphaerica]

MLSSETTIRIFCFFIAVGFAVGSSSPEEEGQLLHVKRETWLNPGFDSMLHRRESQELLNRPRPGRRELFD

LMNQDSILKKRALLHRPRPGRRELFRNQGLDSMLHKRAMLNRPRPGRRELFRNPGLDSMLHKRGQEFLSG

PRPGREIRPRPGRRERHPDSMLHRRSSDTDLDYQHLLRNPRPGRRELFRPRPGHRELQHLDSLLHRRSEE

MWGRPRPGKREVYYEDDGRSEDEKLLRVLDELKRDIIDELWDRFQN

>ARN59426.1 pp7 precursor [Clytia hemisphaerica]

MRFCSWTNLFLLGITCLCLTNGMPNKQHVRNKKNLIDNTVKMADHGKTLVKKSAHPMKIKDVSKKSTGGG

SDIANSDDTFDRAADGTDNSLYGRQEKEQGTENSGVGKFEGPPCRWGCGKREAGIDGPPGRWGGRKRGMR

RVGPPGRWGGRKRGELPGRWGGKKRGELPGRWGGKKRSELPGHWGGKKRSELPGHWGGKKRSEIPGRWGG

KKRSEIPGRWGGKNRSELPLGWSQKEGNQRPPSKET

>ARN59425.1 pp6 precursor [Clytia hemisphaerica]

MARESLLFFLLALHCCEAFYNGDVPRRRSEASRLLMAKDASQKHASSDTSRLLFGKDAPQRHVSSDSRLL

FGKDAPQRHVLSDTSRLLFGKDAPQRHVSSDSRLLFGKDAPQRHISSDSRLLFGKDAPQRHVSSDSRLLF

GKDAPQRHVSSDTSRLLFGKDAPQRHVSSDTSRLLFGKDVPQRPAGGSDSRTSYTAHLMPTLGKDEYVNA

LKERLLNDYRMKLLQQQRQQQHQQQEDDDELYFDHSFRRGNNPSASAFRRRYSDQTGQPLSGSRDQKVNQ

DENARDTLEKKQQASLDQTKEELKRSLLKDFYKKMITEKREKQEFAKKSDAPVSNDFDDEILRHLVEFKL

KKEDPMKRMRR

>ARN59424.1 pp5 precursor [Clytia hemisphaerica]

MELKYFLASFIFVIIAIQLASCSSKAEEYKQMKKEVDGLLKEIVSQENAKQHTSEKKSSQWLNGRFGKRQ

LVSGRFGRELKQWLNGRFGREATEQWLNGRFGKREDDQWLNGRFGREVEQWLNGRFGRDAAEQWLNGRFG

KRSANQWLNGRFGKRSADQWLNGRFGKRSADQWLNGRFGREASADQWLNGRFGREAADQWLNGRFGREAK

EQWLNGRFGREVEQWLNGRFGREAGQWLNGRFGREMGQWLNGRFGREADQWLNGRFGREADQWLNGRF
GR

DAAPLAARYGDEPAHVESQTIAAPEEAKPKIVAAVKVVKKPVAVSE

>ARN59423.1 pp4 precursor [Clytia hemisphaerica]

MNLLVSIPVICAIVLKLTESAPISNVRKIGSNELLLKLTVSDLAKLLSRLQNVHEDGHKEDLNKVSVEGM

IADYLDEKQYKDRPRYGKDLKEASRPRYGKEMSEGGNHNVIEQLIEKLVNQSSESDTKDDGNIKSDGKVD

NLVSLLHGLDEEKEWPRPGKDWPRAGKDWPRAGKDWPRAGKDWPRAGKDWPRAGKDWPRPGKDQPNGIAR

GGKRSLGEILSDLMSKRPRYGKDESSTNGPSYQYTLEDMMTEIISGDRPRYGRKSEASRPRYGKESADKT

LEEALSNLGTKSTKKDTSSRSSLARGGKKRGVEDLILEYIENHLDDKKDYESARKDTPK

>ARN59422.1 pp3 precursor [Clytia hemisphaerica]

MNCVLIFLVLFLANNVYSASLTREEDALVTKLLDTIEKRDAVPRLGKREVPRLGREIEDVPRLGREIELV

PRLGREAEEVPRLGREAEEVPRLGREVELVPRLGREVELVPRLGREVEVPRLGREVPRLGRGIDEVPRLG

REVPRLGRELEVPRLGREVPRLGKREIPRLGKREVPRLGRREVPRLGREASTYDLKQLYNQLKREVSNDM

IEAEIKEEKRVLKAFDLGTRGLILRRIGDKLNKDGFSDQKRDMNEKESSRPFLHVKRTNLLSLIEKLTSE

E

>ARN59421.1 pp2 precursor [Clytia hemisphaerica]

MKIYFGCLFVILSVNQIGCYPSSNQNSERELVRRIYKTVHPNPHYQVNEIQRVKEALKRRVLENVHRVDS

LKASLKRVLGQDAGNGFHMSSSKIFQKKRSKARLPHSYMFRKRQNSPGALGLWGREVEAPGDIGPPGIWG

DVVPDETRKDKPGAVQGLWGKDERVIRALLKTLKR

>ARN59420.1 pp1 precursor [Clytia hemisphaerica]

MERKILACLFLLIVLLNLNDGKNIAILIEPDDNLASELEWLGSDMTDSHSLNAGAWPRPGDARSSHDAWP

RPGKREFYGNEMFEKRPFPGQQMQFSWPRPGKKETKEDTWPRPGKRESYSEGDMDSRSGALRRSEEKETN

EDEKLENAWPRPGKREFYASRKMDVRPRGGRDSKSHKISKRNSEAISNDEIDMMIREEAWPRPGKRDYHM

LSATRPRGGKDARPRGGKDSSRPRGGKNAKPRGGKDSVRPRGGKKDSWPRPGKDAFVKEINGSRPRGGKD

ASKWPRPGKKDLK

>tr|O76948|O76948_HYDVU Neuropeptide OS=Hydra vulgaris OX=6087 PE=2 SV=1

MLSNKKVELLFALVFVVVAVVRSEDKKLSLEDNKDVKRIVNDYLETKNGEQLMSGRFGKR

ETDEADSDDEDSSEYENEYDDELENQGLANARYERQLMRGRFGREKNAASNEDQWLGGRF

GREAATQWFNGRFGRDIEGRFLPRFAKESNKPHLRGRFGRAVKL

>tr|O76947|O76947_HYDVU Neuropeptide OS=Hydra vulgaris OX=6087 PE=2 SV=1

MATNMALLTFILFATSIFMLAKADSQNEDNQKYAGIARSLKVLLQNYYQKQEEKSDIQNI

IEKFSEYQNTDHKRNDKTNPMFEKKDSDTENRFNREAIEQWFSGRFGLPNQKRNNEVNPM

IEKKDSDIENRFNRESLEQWLSGRFGLTNQKRHNEANPMIEKKDSDTENRFNKETIEQWL

SGRFGLTNHKRNNEVNPMIEKKDSDTENRFNRESLEQWLSGRFGLTNHKRNNEVNPMIEK

KDSDTENRFNRESLEQWLSGRFGLTNHKRNDEANPMIEKKDSDTENRFNRESLEQWLSGR

FGLTNHKRNNEVNPMIEKKDSDTENRFNRESLEQWLSGRFGLTNHKRNDEVNPMIEKKDS

ENENRFNRESIEQWLGGRFGRTVYEFLLSETSEKRKK

>tr|A8C983|A8C983_HYDVU FRamide OS=Hydra vulgaris OX=6087 GN=FRa PE=2 SV=1

MYLRLLVVFFVLQISLQESNVRELDLGKLIEDYLAKENVRREEFLNKINTEILRYIYELE

NENKGKRRIEASADKNVLEKVLTEVPSIRESAMSKESNVNKVHNSLDSKSSIRSIPTGTL

IFRGKKESNSNNENTSEQGAPGSLLFRGKKEPNLKENSKNETEASHGERLQQTERNFLVK

TKEYIEKLLNSGEEIV

>tr|O76949|O76949_HYDVU Neuropeptide OS=Hydra vulgaris OX=6087 PE=2 SV=1

MLSYKKFELLFALVLIVVEVVKSDDKNFSLEVNKDVKRFIKDILDAKSEEQLMSGRFGKS

LPDEEDIDNEVENEYDNEYDDETESQGIINGRYGRQLLRGRFGRQNDNNAASKENQWLGG

RFGKEVATQWFNGRFGREIGGRFLPRFAREFNKPHYRGRFGRIAKL

>tr|Q9U8U7|Q9U8U7_HYDVU Hym-355 preprohormone OS=Hydra vulgaris OX=6087 GN=Hym-355 PE=2 SV=1

MLSLTVATLLLITSIVMAMPNRDATDSNESDILNILDEYIVKVAEMTANEAKILNDVRNY

YNDRSSKSLGEFPQSFLPRGGKRDARPRAGK

>tr|O77060|O77060_HYDVU Hym-176 preprohormone OS=Hydra vulgaris OX=6087 GN=Hym-176 PE=2 SV=1

MSKINKLTMYVFYALLVLNIYVVLSVNSLPFRDDEDTDNEIDGDISELENEYQTNQVYDY

NKFKNQADLKIKARNHYAPFIFPGPKVGRDVNFHSVLSPSDESRKSFNNYHENGYRHDKP

AFLFKGYKPGDQTQKNL

>tr|Q71SB0|Q71SB0_HYDVU KVamide preprohormone type 2 OS=Hydra vulgaris OX=6087 PE=2 SV=1

MEKTNKLIRLVLNAFLALNIFMVMSVNSMPFHDNEDTDDKISSDINILKNESQSSQINDY

NKYQKISTIKGRLQYYPFYNQNPKVGRDVSFHSAQDASDKGRMKKLTYIYNKNEYRKDKP

LYLFKGYKPGDQTQMHF

>tr|P91736|P91736_HYDVU LW-amid and MW-amid-containing preprohormone OS=Hydra vulgaris OX=6087 PE=2 SV=1

MGMFERKKIVLLVSLICVSQQATNVQDANSKSTSTELKVVKPQKRVTPVKDAEKLSILRT

QDNSLDLNTNGEEVWDELTHNIPLEYIEKIYNELNQLAQNENRPKRLWGATAAINTDNLN

PEVENELENKKNAPVIEKFERPIGLWHKDIETKNPENRLPLGLWGKDSEPLPIGLWGKDA

DVNDDLKKEPLPIGLWGKDTDSTRGDNKPNAYKGKLPIGLWGKDNALTNDLGKKNNGKDS

GPPPGLWGKDSKPIPGLWGKDNGPMTGLWGKKDVGPPPGLWGKKDQPPIGMWGRAGKRDS

NPYPGLWGKKEEELENVDKEIEEDSLEEFPACLLENPPCEIQEKRYNIDKSGPPPGLWGK

RSEKYQMNKPPWRGGMWGRSEILENSVHDSKKTNTIDMEHAEN
